# Systematic review of prognostic models for predicting recurrence and survival in patients with treated oropharyngeal cancer

**DOI:** 10.1136/bmjopen-2024-090393

**Published:** 2024-12-05

**Authors:** Janine Dretzke, Ahmad K Abou-Foul, Esther Albon, Bethany Hillier, Katie Scandrett, Malcolm J Price, David J Moore, Hisham Mehanna, Paul Nankivell

**Affiliations:** 1 Department of Applied Health Sciences, College of Medicine and Health, University of Birmingham, Birmingham, UK; 2 Institute for Head and Neck Studies and Education, Department of Cancer and Genomic Sciences, College of Medicine and Health, University of Birmingham, Birmingham, UK; 3 Department of Public Health, Canadian University Dubai, Dubai, UAE

**Keywords:** systematic review, prognosis, head & neck tumours

## Abstract

**Abstract:**

**Objectives:**

This systematic review aims to evaluate externally validated models for individualised prediction of recurrence or survival in adults treated with curative intent for oropharyngeal cancer.

**Design:**

Systematic review.

**Setting:**

Hospital care.

**Methods:**

Systematic searches were conducted up to September 2023 and records were screened independently by at least two reviewers. The Prediction model Risk Of Bias ASsessment Tool was used to assess risk of bias (RoB). Model discrimination measures (c-indices) were presented in forest plots. Clinical and methodological heterogeneity precluded meta-analysis.

**Results:**

Fifteen studies developing and/or evaluating 25 individualised risk prediction models were included. The majority (77%) of c-indices for model developments and validations were ≥0.7 indicating ‘good’ discriminatory ability for models predicting overall survival. For disease-specific measures, most (73%) c-indices for model development were also ≥0.7, but fewer (40%) were ≥0.7 for external validations. Comparisons across models and outcome measures were hampered by heterogeneity. Only two studies directly compared models in the same cohort. Since all models were subject to a high RoB, primarily due to concerns with the analysis, the trustworthiness of the findings remains uncertain. Concerns included a lack of accounting for potentially missing data, model overfitting or competing risks as well as small event numbers. There were fewer concerns related to the participant, predictor and outcome domains, although reporting was not always detailed enough to make an informed decision. Where human papilloma virus (HPV) status and/or a radiomics score were included as a variable, models had better discriminative ability.

**Conclusions:**

There were no models assessed as being at low RoB. Given that HPV status or a radiomics score appeared to improve model discriminative performance, further external validation of existing models to assess generalisability should focus on models that include HPV status as a variable. Development and validation of future models should be considered in HPV+ or HPV− cohorts separately to ensure representativeness.

**PROSPERO registration number:**

CRD42021248762.

**STRENGTHS AND LIMITATIONS OF THIS STUDY**

- Sensitive search strategies were used to ensure as many relevant studies as possible were included in the review.
- Thorough risk of bias assessment of included studies was undertaken using the Prediction model Risk Of Bias ASsessment Tool.
- Only models with at least one external validation were included in order to focus on those that may be generalisable and suitable for implementation in practice.
- Clinical and methodological heterogeneity precluded meta-analysis of model performance measures.
- Poor reporting of details on model development and validation in included studies hampered risk of bias assessment and thus meant that trustworthiness of results was uncertain.

## Introduction

 Head and neck cancer is the seventh most common cancer worldwide, with a rising incidence driven largely by increasing cases of oropharyngeal cancer (OPC).^1 2^ Major risk factors for OPC are smoking, alcohol consumption and infection with human papilloma virus (HPV).^2^ Specific treatment approaches depend on cancer stage, patient comorbidities and risk of recurrence, while taking into account preservation of function.^2^

Prognostic information may be useful both for planning treatment and patient counselling. Patients at low risk of recurrence, for example, may be candidates for treatment de-escalation trials, while patients with high risk of recurrence may benefit from more intensive treatment.^3 4^ Intervention decisions may be contingent on a model being able to account for sequential interventions and the associated risks.^5^ The American Joint Committee on Cancer (AJCC)/International Union Against Cancer staging system based on tumour characteristics (T), nodal spread (N) and distant metastasis (M) is used for classifying patients into risk groups for prognosis, and often to plan treatment options.^6^ The most recent version (eighth) incorporates HPV status in order to improve prognostic accuracy in OPC. Nonetheless, there are limits to how useful the TNM system is on an individual patient level.^7^

Several prognostic models have been developed with the aim of predicting survival and recurrence of OPC. Two systematic reviews of such models currently exist (with searches up to 2018); however, there are also models developed and evaluated more recently and both reviews have limitations.^8 9^ One review excluded studies which focused on recurrence^9^ and the other included models that had not been externally validated, and excluded studies undertaking an external validation only.^8^ This systematic review aims to include, appraise and summarise all the existing evidence from externally validated models used for predicting recurrence or survival in adults who have been treated with curative intent for OPC.

## Methods

The protocol was registered with PROSPERO (CRD42021248762) for a systematic review of prognostic models in all subtypes of head and neck cancer.^10^ Findings related to OPC are reported here. Reporting is in accordance with the Preferred Reporting Items for Systematic reviews and Meta-Analyses (PRISMA) guidelines (online supplemental material 1).

### Searches

Searches were undertaken in MEDLINE and MEDLINE In Process (OVID), Embase (OVID) and the IEEE database from 2005 to September 2023, with no restriction by language or publication type. Searches combined text and index terms related to head and neck cancer, prognostic models and recurrence and survival (online supplemental material 2). This search strategy was performed as part of a systematic review of prognostic models in all types of head and neck cancer, and specific terms related to OPC were included. Terms for prognostic models were based on the filter defined by Geersing *et al*.^11^ Reference lists of included articles and relevant reviews were also checked, and subject experts were consulted.

### Selection criteria

Models were included if they predicted any recurrence or survival-related outcomes after treatment of OPC with curative intent, included at least one clinical variable and had at least one reported external validation (online supplemental material 3).

### Study selection

Titles and abstracts were independently screened by at least two reviewers (EA, JD, AKA-F, DM) using Rayyan software (http://rayyan.qcri.org, Qatar Foundation, Qatar). Full texts were obtained where needed to determine eligibility. Due to a large number of records, full texts were not sought if there was no mention of any form of validation in the abstract. Disagreements on inclusion/exclusion were resolved through discussion or referral to the wider steering committee. Risk of bias (RoB) assessment was performed after study selection and level of RoB was not an eligibility criterion. The screening process was documented in a PRISMA flow diagram.

### Data extraction

Data were extracted by one reviewer using a predesigned and piloted data extraction form and checked by a second reviewer (JD, AKA-F, EA). Disagreements were resolved through discussion. Information was extracted on patient characteristics for each development and external validation cohort, study design, model variables, outcomes (overall survival (OS) and any disease-specific measure such as progression-free survival (PFS) or recurrence-free survival (RFS)) and model performance measures (for each time point reported, eg, 2-year and 5-year OS).

### Risk of bias assessment

The Prediction model Risk Of Bias ASsessment Tool (PROBAST) was used to assess RoB and applicability.^12^ Each model development and each external validation of models was assessed separately. Assessment was conducted by one reviewer (JD, AKA-F, BH, KS, EA, MP) and independently checked by one of the two lead reviewers (JD or AKA-F), with referral to the other in case of ambiguity or disagreement with the first reviewer. A list of criteria was developed with the wider steering group to help facilitate RoB decisions (online supplemental material 4). PROBAST assesses RoB across four domains (participants, predictors, analysis and outcomes). An overall rating of ‘high’, ‘unclear’ or ‘low’ RoB was given to each model; an overall judgement of high RoB was made where at least one domain had high RoB. Applicability refers to the extent to which included models match the systematic review question in terms of participants, predictors and outcomes. Formal ratings for applicability were not generated, but judgment were informed by PROBAST guidance.

### Synthesis

Model discrimination measures (c-indices) were presented in forest plots where possible, grouped by outcome (OS, PFS or other disease-specific measures) and by model. Thresholds for the c-index of <0.5, <0.7, >0.7 and >0.8 were used to indicate poor, weak, good and very good discriminatory ability, respectively.^13^ We acknowledge these cut-offs are to an extent arbitrary and were chosen for pragmatic presentation purposes. Quantitative pooling was not undertaken due to differences in population, length of follow-up, metric used (c-statistic or area under the curve (AUC)) and a lack of uncertainty measures (CIs). There were also differences in model parameters and outcome ascertainment (for PFS), although this was not well reported. C-indices were reported for all follow-up times where available, and both the c-index and AUC were presented where they differed. Model calibration statistics, along with other performance metrics, were described narratively. A formal exploration of small study effects using funnel plots was not possible.

### Patient and public involvement

Patients or the public were not involved in this systematic review.

## Results

**Figure 1:**
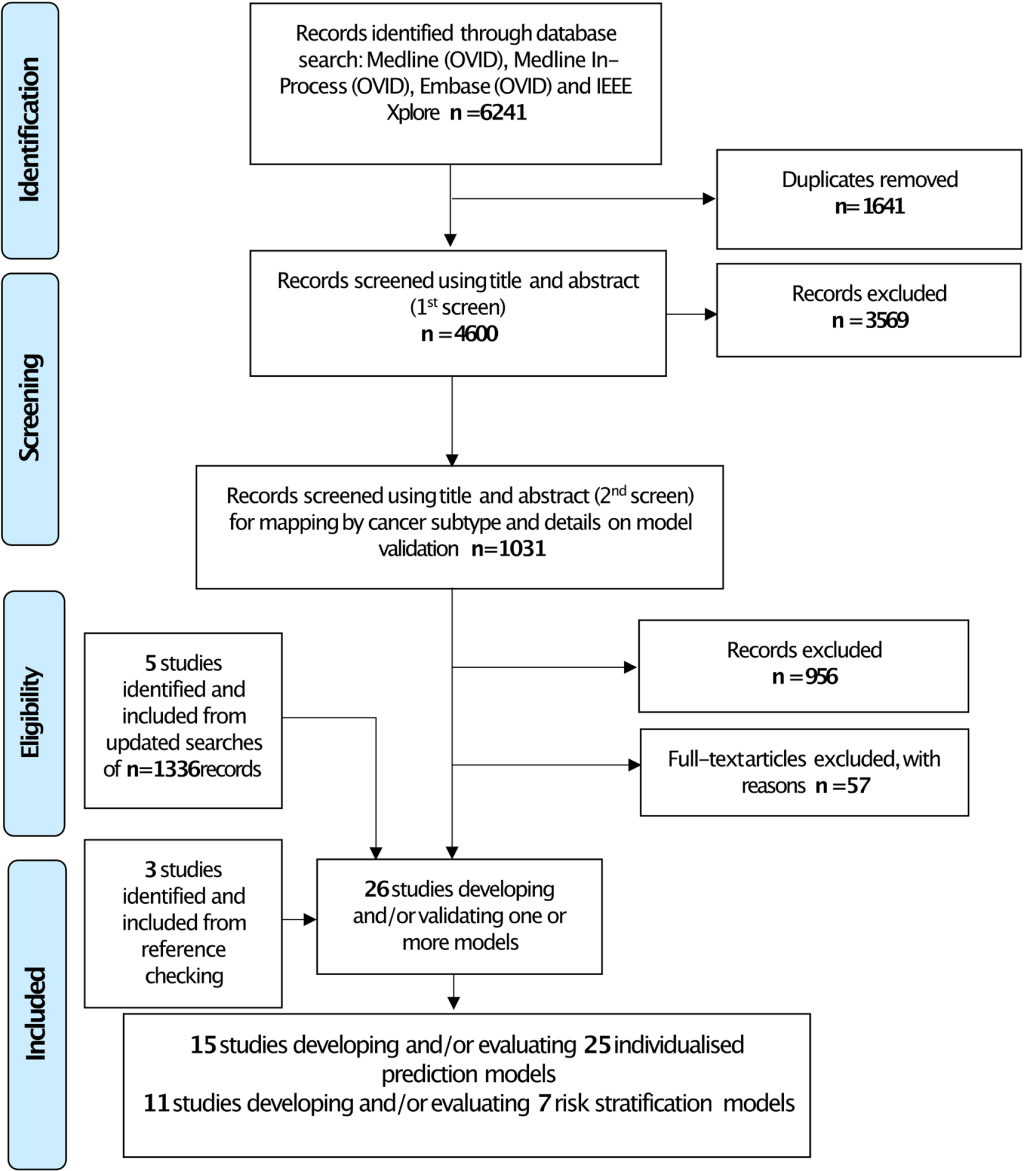
Preferred Reporting Items for Systematic reviews and Meta-Analyses flow diagram.

From 5936 records screened, 15 studies were included. Using the Transparent Reporting of a multivariable prediction model for Individual Prognosis Or Diagnosis (TRIPOD) classification,^14^ there was one type 1b study^15^ (development and validation using resampling), 10 type 3 studies^316^,^24^ (development and validation using separate data) and four type 4 studies^25^,^28^ (validation only). The 15 studies reported a total of 25 models to predict individualised outcomes (see figure 1 for full details on study selection). The 25 models were externally validated 43 times, reported across 14 studies^316^,^28^ (the remaining study^15^ reported the development of a model that was evaluated in other studies). Most models were externally validated once or twice; the OS model by Fakhry *et al*^18^ was externally validated in five independent cohorts, and the OroGrams OS and PFS models^19^ were externally validated in four independent cohorts. All model development studies and their associated external validation studies are shown in online supplemental material 5.

An additional 11 studies developing and/or evaluating seven ‘risk stratification models’ were identified.^29^,^39^ One study was reported as an abstract only and was not taken forward for analysis as full RoB assessment was not possible.^40^ The main reasons for exclusion were: a lack of external validation; a model for head and neck cancer with no subgroup analysis for OPC; model parameters based on radiomics or genetics only or conference abstracts of an included full text (online supplemental material 6). No model impact studies were identified.

### Risk stratification models

The seven ‘risk stratification’ models did not generate individualised predictions as the model outcome, but instead classified patients into broader risk categories.^29^,^39^ The RTOG-0129 RPA model by Ang *et al*^29^ was externally validated in eight separate cohorts reported in seven studies.^1823 30 31 35^,^37^ Other ‘risk stratification’ models were those by Rietbergen *et al*^35^ (validated in two studies), Huang *et al*^32^ (validated in two studies) and O’Sullivan *et al*^34^ (externally validated within the same study). The latter two models undertook restaging of TNM groupings using different methods, while the models by Ang *et al*^29^ and Rietbergen *et al*^35^ stratified patients into risk groups based on HPV status, T-stage and N-stage and either smoking^29^ or comorbidity (adult comorbidity evaluation (ACE)).^35^ A ‘risk stratification’ model based on machine learning (ProgTOOL) was developed and evaluated by Alabi *et al*, and stratified patients based on age, sex, ethnicity, marital status, tumour grade, T-stage, N-stage and M-stage, type of treatment and length of disease-free survival.^38 39^ Model performance assessment was mostly limited to the c-index. This ranged from weak to good (c-indices between 0.58 and 0.76), and discriminative ability was mostly lower than that of the individualised risk prediction models (IPMs). The overall PROBAST RoB rating was high for all ‘risk stratification’ models, mainly due to concerns about RoB in the analysis domain (online supplemental material 7).

### Individualised prediction models

The main study and population characteristics for the IPMs are shown in online supplemental material 8. All model development studies and evaluations were based on retrospective analyses of data. Patients were typically drawn from a single institution (66% of cohorts), and less often from multiple institutions or registries. Median population ages were between 53 and 64 years; no studies including people aged <18 years were identified. Fakhry *et al* used patients enrolled in trials for both development and validation of their model.^18^ All but one study cohort (97%) included both HPV+ (18%–78%) and HPV− (10%–82%) patients. Mes *et al* included only HPV− patients.^21^ The majority of patients were treated with curative intent (89%, where clearly reported), although not all studies had an explicit statement on this. Two study cohorts included up to 6.7% of patients treated with palliative care.^15 25^ There was variability across cohorts in terms of proportions receiving different treatments (chemoradiotherapy (CRT) and/or radiotherapy (RT) alone, surgery±CRT or RT). Smoking was reported in different ways; where the proportion of current smokers was provided, it varied between 32% and 83%. Alcohol consumption was rarely reported.

The variables included in each of the individualised prediction models are shown in online supplemental material 9. All models included T-stage and/or N-stage and all but two (92%) included age and/or sex. Other commonly included variables were HPV status (75% of models), smoking (48%), performance status (44%), overall cancer stage (28%) and ACE comorbidity score (24%). Nine models included CT-based,^17 20^ MRI-based^21^ or FDG-PET-derived^16^ radiomic features, and none included genetic variables. Six models^20 21^ directly employed a curated set of these features in the modelling process, and three models^16 17^ used a calculated radiomic score in their final models. Notably, only one model clearly reported the final radiomic features used in the predictive model.^17^ Online calculators are available for seven models (online supplemental material 5).

#### Risk of bias and applicability

**Figure 2:**
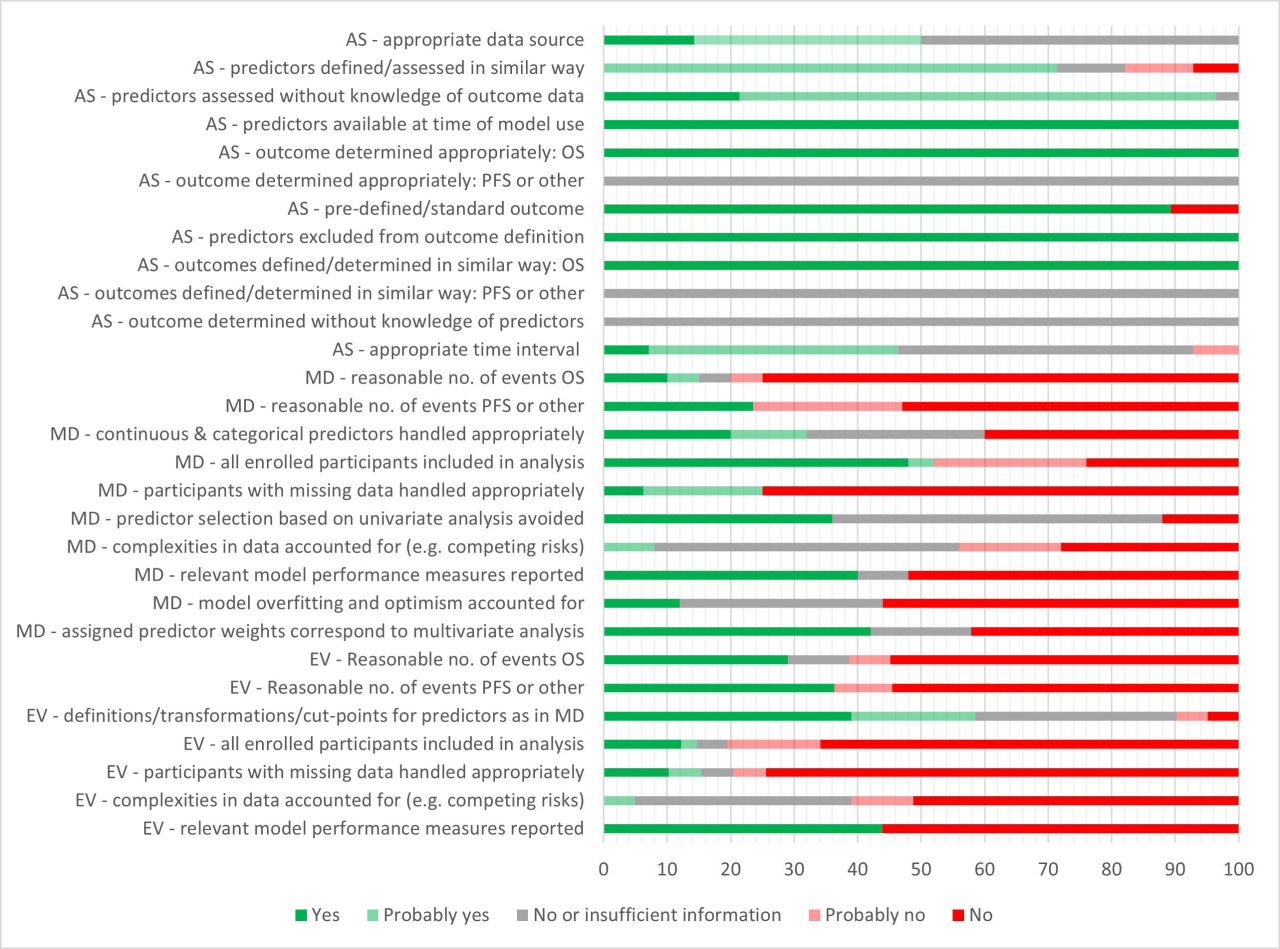
Prediction model Risk Of Bias ASsessment Tool summary chart shows percentage of study cohorts meeting/not meeting criteria: AS, all study cohorts; EV, external validation cohorts; MD, model development cohorts. Number of cohorts contributing to the different criteria varies (eg, as not all evaluations report both overall survival (OS) and progression-free survival (PFS); the criterion ‘participants with missing data handled appropriately’ is only applicable where there was missing data). Every evaluation counted for the analysis domain; some cohorts were used for evaluating more than one model. The criterion ‘all enrolled participants included in analysis’ was answered with ‘no’ if participants were excluded on the basis of missing variable data. Where there were several disease-related outcomes (such as PFS, disease-free survival (DFS)), the question (‘was there a reasonable number of events?’) was answered with NO if the number of events was considered to be too low for at least some of these.

A total of 68 RoB assessments were undertaken: 25 for model developments and 43 for external validations of models (PROBAST domain ratings for each assessment are presented in online supplemental material 10). The overall PROBAST RoB rating was high for all but one of the IPM assessments, mainly due to concerns about bias in the analysis domain (figure 2). One assessment of an external validation was rated as having insufficient information to make a judgement on overall bias.^3^ Main areas of concern included the enrolment of participants based on available variable data, with no attempt to account for potentially missing data; small numbers of events (deaths or recurrence), which may lead to bias in outcome prediction (small number of events were considered to be ≤10 events per candidate predictor for development and <100 events for validation cohorts); a lack of accounting for model overfitting and optimism (in development studies) and a lack of accounting for complexities of the data (such as competing risks). Around half of both the model development studies and model validation studies did not report relevant model performance measures. There were fewer concerns related to the participant, predictor and outcome domains, although reporting was not always detailed enough to make an informed decision. It was unclear whether outcomes were determined without knowledge of predictor information and whether recurrence was determined in a similar way for all participants. Some poor PROBAST ratings may in part be due to poor reporting rather than a true high RoB. Nonetheless, based on the information reported, there were no models that stood out as being of markedly lower RoB than others. Regarding applicability, all studies matched the review question in terms of population, predictors and outcome, although there were two studies where a minority (up to 6.7%) of patients were not treated with curative intent.^15 25^

Five models reported in three studies (Rasmussen *et al*,^22^ Beesley *et al*^3^ and Grønhøj *et al*^19^) met 50% or more of the analysis domain items for model development. The development and validation cohorts for these models appeared to be reasonably representative of OPC populations to whom the models might be applied. However, the development cohort by Grønhøj *et al*, which had a high proportion of HPV+/p16+ patients (approximately 60%), unexpectedly included a larger than usual proportion of smokers (around 80%). This is higher than what is typically seen in clinical practice and reported in the literature for this group of patients.^19 41 42^ One of the four external validation cohorts for this model also had a high proportion (>50%) of stage IV disease compared with the other cohorts.^19^ The development cohort by Rasmussen *et al*^22^ was almost identical to that of Grønhøj *et al*^19^ in terms of the included patients. The study by Beesley *et al* included a development cohort from the USA that was predominantly HPV+ or p16+, while the external validation cohort from the Netherlands was primarily p16−. This variation aligns with the known geographical differences in the prevalence of HPV+ oropharyngeal squamous cell carcinoma (OPSCC) and is still considered representative of unselected OPC patient populations.^3 43^ Further applicability issues are noted in the ‘Discussion’ section.

#### Model performance: overall survival

**Figure 3:**
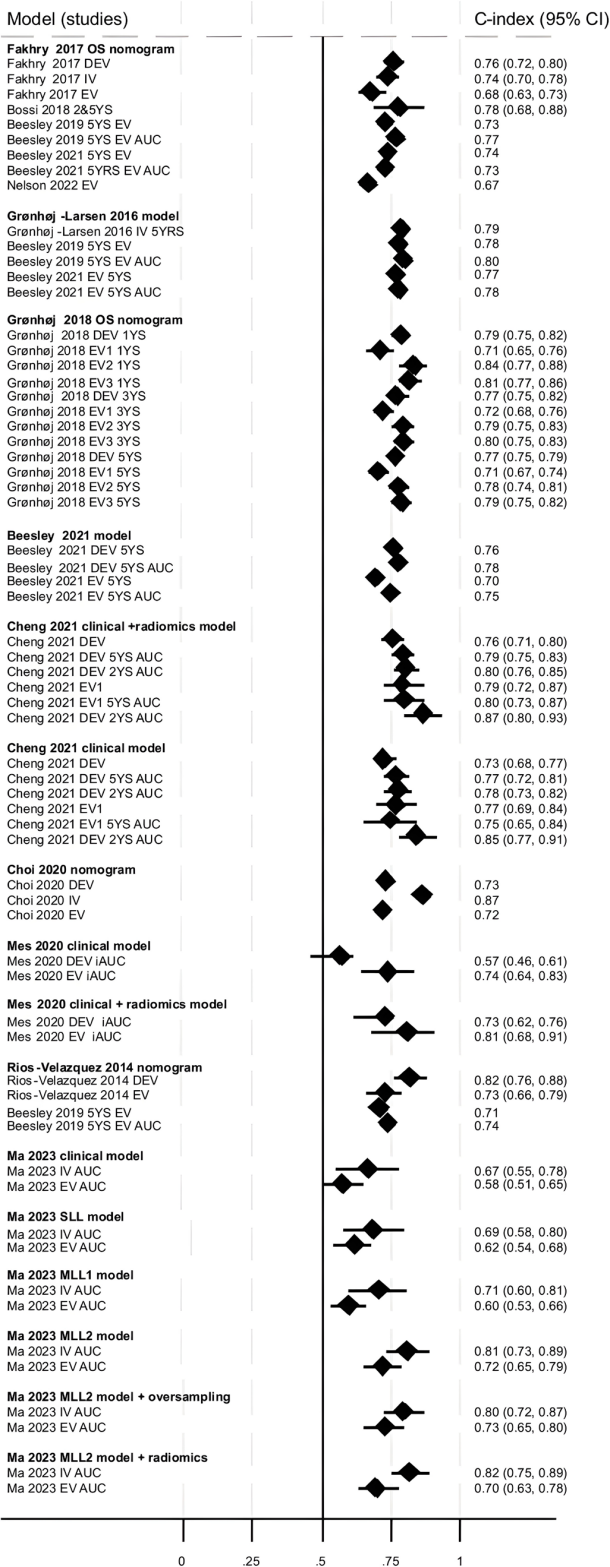
Discriminatory ability of models to predict overall survival. All c-indices, area under the curve (AUC) values and time points presented (where reported); some studies did not present CIs. DEV=development; EV=external validation; iAUC=integrated AUC; IV=internal validation; MLL=multi-label learning; OS=overall survival; SLL=single-label learning; YS=year survival. Data from Cheng *et al*^16^ clinical model (±radiomics score) are presented here. Data for the remaining Cheng *et al*^16^ models are available in .

Discriminatory ability for OS was assessed by 20 models reported across nine studies and all reported c-indices. The model developed by Fakhry *et al*^18^ was externally validated five times,^3 18 25 26 28^ the model by Gronhoj *et al* three times,^19^ the model by Gronhoj-Larsen *et al*^15^ twice,^3 25^ the six models by Cheng *et al* twice,^16^ the model by Rios-Velazquez *et al*^23^ twice,^23 25^ the model by Beesley *et al* once,^3^ the two models by Mes *et al* once,^21^ the model by Choi *et al* once^17^ and the six models by Ma *et al* once.^20^ The c-index (or AUC where c-index not reported) was ≥0.7 (‘good’) for the majority of development studies (17/22 (77%)), but only a few (4/22 (18%)) had a c-index ≥0.8 (‘very good’). This was similar for external validations across all models, with the majority (27/34 (79%)) reporting a c-index ≥0.7, with few external validations (4/34 (12%)) resulting in a c-index of ≥0.8 (‘very good’) (figure 3). This was also the case for those models with lower RoB for model development RoB assessment (Beesley *et al*^3^ and Grønhøj *et al*^19^; OS not predicted in Rasmussen *et al)*^22^, although we acknowledge that they were still rated as ‘high’ RoB using PROBAST. Two studies reported c-indices for different times points: 2 and 5 years (Cheng *et al)*^16^ and 1, 3 and 5 years (Grønhøj *et al)*^19^. C-indices were similar or slightly lower at later time points.

The Mes *et al* clinical model (which includes N-stage, age and sex) had a markedly lower c-index for the development cohort (0.57 (95% CI 0.46, 0.61)) compared with the same model including radiomics features (0.73 (95% CI 0.62, 0.76)); this study was in HPV− patients only.^21^ Adding a radiomics score also appeared to improve the Cheng *et al*^16^ clinical model slightly; the clinical model included HPV status, T-stage and N-stage, TNM stage, age and sex. Excluding HPV status from these models appeared to slightly reduce the discriminatory ability of both the clinical and clinical+radiomics models, respectively (data not shown in plot). All other Cheng *et al*^16^ models included HPV (or p16) status. The Ma *et al* clinical model was also slightly improved with the addition of CT-derived radiomic features.^20^ Four studies^3 16 23 25^ also reported a c-index for TNM staging; these were consistently lower than those reported for the IPMs, although discriminatory ability was improved with TNM8 compared with TNM7 (based on one study).^25^

Model calibration was reported for the external validation cohort in Beesley *et al* model and the observed OS was similar to predicted OS.^3^ Calibration of the Grønhøj *et al* model^19^ was slightly variable depending on the cohort; Brier score for the development and three external validation cohorts suggested reasonably good model performance (values <0.2), with model performance decreasing with follow-up time for predictions (online supplemental material 5).

#### Model performance: disease-specific measures

Discriminatory ability was presented for various disease-specific measures: PFS, RFS, event-free survival (EFS), disease-specific recurrence (DSR), disease-specific survival (DSS), T-site, N-site and M-site recurrence, local control (LC), regional control (RC), locoregional control (LRC), distant metastasis-free survival (DMFS), disease-free survival (DFS) and death with no evidence of disease. Fifteen models across 10 studies reported c-indices or AUC.^318^,^24 26 28^ There were three models for PFS (Fakhry *et al*,^18^ Gronhoj *et al*^19^ and Rios-Velazquez *et al)*^23^, two of which were externally validated three times,^18 19^ and one that was externally validated once.^23^ Two models developed by Mes *et al* for RFS were externally evaluated once,^21^ one model for EFS (by Beesley *et al*) was evaluated once,^3^ seven models for DSS (one by Ward *et al*^24^ and six by Ma *et al)*^20^ were evaluated once,^20 24^ six models all developed by Ma *et al* were evaluated once, for each of LC, RC, LRC, DMFS and DFS,^20^ and one model (by Rasmussen *et al*) was evaluated once for T-site, N-site or M-site recurrence.^22^

**Figure 4:**
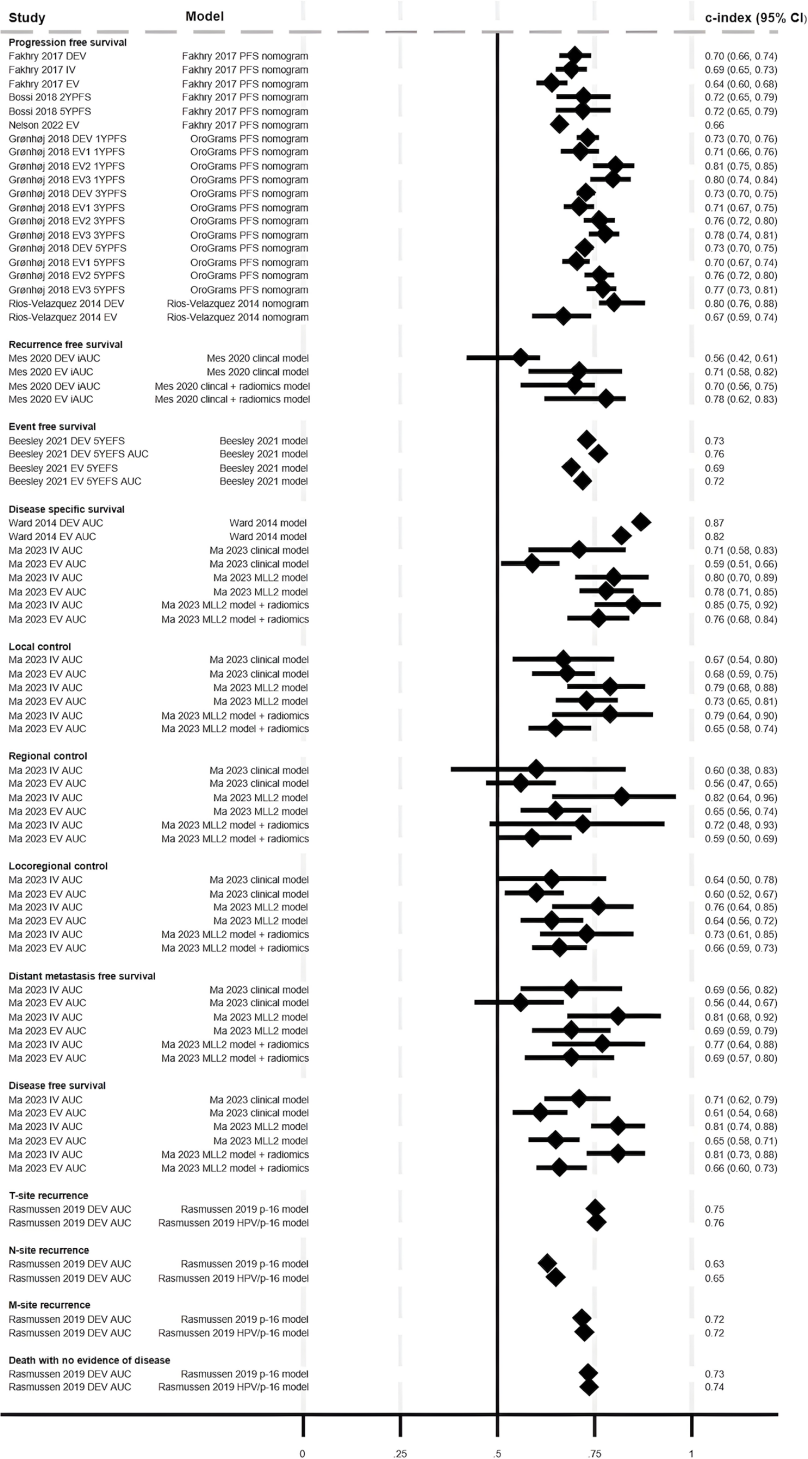
Discriminatory ability of models to predict disease-specific outcomes. All c-indices, area under the curve (AUC) values and time points presented (where reported); some studies did not present CIs. DEV=development; EV=external validation; IV=internal validation; YS=year survival. Data from Ma *et al*^20^ clinical model and MLL2 model (±radiomics score) are presented here. Data for remaining Ma *et al*^20^ models are available in online supplemental material 5.

The c-index (or AUC where c-index not reported) was ≥0.7 (‘good’) for 73% (36/49) of development studies and for 40% (23/58) of external validations across all models. Only 22% (11/49) of development and 5% (3/58) of external validation studies found a c-index of ≥0.8 (‘very good’) (figure 4). Given the variability in models and disease-specific measures, comparison of model performance across studies and outcome measures is difficult. The Mes *et al*^21^ clinical model (which includes N-stage, age and sex) had a markedly lower c-index for RFS for the development cohort (0.56 (95% CI 0.42, 0.61)) compared with the same model with an added radiomics features (0.70 (95% CI 0.56, 0.75)). Rasmussen *et al* (development cohort) reported slightly lower AUCs for N-site recurrence compared with T-site recurrence, M-site recurrence and death with no evidence of disease.^22^ High AUCs were reported in Ward *et al* for DSR (AUC=0.87, 95% CI not reported, for development; AUC=0.82, 95% CI not reported, for external validation). This model included T-stage, smoking and tumour-infiltrating lymphocytes.^24^ Ma *et al* reported higher AUCs for some disease-specific outcomes with the multilabel learning models (incorporating CT-derived radiomics) compared with the clinical or single-label learning models, the latter also incorporating CT-derived radiomic features.^20^

Model calibration was reported for the external validation cohort in Beesley *et al* and observed EFS was similar to predicted EFS.^3^ Brier score for the Grønhøj *et al* model development and external validations suggested reasonably good model performance (values <0.2), with model performance decreasing over time.^19^ Brier score suggested that there was no statistical evidence of a difference in model performance between the p16 model and the HPV/p16 model for PFS (Rasmussen *et al*, online supplemental material 5).^22^

## Discussion

### Principal findings

Our systematic review has identified a large number of OPC prediction models in the literature, with all of the currently available IPMs introduced after 2014. The IPMs for OS mostly scored >0.7 for discrimination when externally validated, although no models consistently produced c-indices above 0.8. Given the high RoB ratings, it is uncertain how trustworthy these scores are. There were no pronounced differences in model performance between models scoring slightly higher or lower on RoB assessment. This lack of difference in performance could be due to the fact that (i) RoB was universally high according to PROBAST even where there were some individual differences, (ii) the cut-off for lower/higher RoB was arbitrary (50% of analysis domain items met/not met) and (iii) RoB ratings were dependent on the information reported, with poor ROB ratings potentially due to poor reporting rather than true RoB. C-indices for OS and disease-specific measures were also similar where the same model reported both outcomes. The comparison of the c-indices across models is hampered by the fact that most have been evaluated in different cohorts, so overall conclusions about which model performs best are not possible. Furthermore, reliance on c-index alone in the absence of calibration measures is insufficient for assessing overall model performance.

Most models in this review were only validated in one or two cohorts. The OS and PFS models by Grønhøj *et al*^19^ were validated in four cohorts with reasonably consistent model performance suggesting that it may be widely applicable. Model performance was slightly lower (based on c-index) in one external validation cohort, which comprised a higher proportion of HPV− patients and smokers than the other cohorts. The OS and PFS models by Fakhry *et al*^18^ were validated in five cohorts, also with reasonably consistent model performance, although with slightly lower c-indices for some validations. The Fakhry *et al*^18^ models were developed in a trial population, which may not be as representative as a more general population, and one external validation (Nelson *et al*)^28^ used surrogates for some model variables, which could potentially explain the slightly poorer discriminative ability achieved with this cohort. The Beesley *et al* model was developed in a cohort with mostly p16+ patients and externally validated in a cohort with mostly p16− patients, which could potentially suggest wider applicability of the model; c-indices for OS and EFS were however slightly lower in the validation cohort.^3^

### Previous systematic reviews

A systematic review by Tham *et al* included 44 published HNC nomograms, and judged their quality against the AJCC Precision Core Medicine (PMC) criteria.^9^ The authors concluded that a significant proportion of the nomograms had serious design flaws, such as small numbers of deaths (events) in their validation cohorts. Small event numbers can increase the risk of model overfitting and reduce stability of the subsequent individual risk predictions.^44^ Moreover, none of the nomograms reviewed in that study fulfilled all of the AJCC-PMC’s criteria, as they lacked satisfactory description of the inclusion/exclusion criteria and treatments that patients received. Additionally, calibration was often poorly reported.^9^ These findings concur with our RoB findings. All included IPMs had a high RoB, based on the PROBAST assessment. Since this likely reflects poor reporting to an extent, it was difficult to gauge whether some models were developed using better methods than others. Our assessments are also in line with those of Palazón-Bru *et al*,^8^ whose systematic review included some of the same studies. Poor reporting of sufficient criteria to allow full assessment of model development and validation is a known problem in prognostic research.^45^

### Comparison with traditional risk stratification using the TNM system

Risk stratification for patients with OPSCC has traditionally relied on the AJCC TNM staging system, which uses a rigid ‘bin model’ to stratify patients into different staging groups.^46^ However, the TNM system was primarily intended to describe the anatomical extent of the disease, and its pretreatment risk estimates can only be applied to the whole stage grouping, rather than providing individualised risk predictions.^7 47^ Moreover, the TNM system only uses anatomical and histological pretreatment variables, and does not consider the impact of treatment on disease outcomes. The AJCC responded to the rapidly emerging HPV-associated OPSCC by updating the TNM system in 2016 (eighth edition) to include a biomarker in HNC for the first time, p16 or HPV status, in patients with OPSCC.^48^ Models included in this review used either the seventh or eighth edition for defining the TNM status model variable. While we would not expect this to substantially affect model performance of the individual models (median c-indices were similar between TNM7 and TNM8 groups, online supplemental material 5), there are external applicability concerns, for example, where a model developed in a population staged by TNM7 is applied in a new population staged by TNM8. Four studies included in our systematic review evaluated the performance of the TNM system alone.^3 16 23 25^ In all cases, the performance (based on c-index) was inferior compared with any IPMs evaluated in the same cohorts.

### Model parameters in included models

HPV status is considered to be an important prognostic factor in OPC.^49^ As survival differs between HPV+ and HPV− patients, a model is likely to be most useful if it incorporates HPV status. The only models that did not include HPV (or p16) status were those developed by Cheng *et al*,^16^ Mes *et al*^21^ and Ward *et al*.^24^ The Cheng *et al*^16^ models suggested that exclusion of HPV may result in poorer performance of the discrimination, although this appeared to be mitigated by inclusion of a radiomics score. The performance of the Mes *et al*^21^ model also suggested better discriminative ability when radiomic features were included (in the absence of HPV as a variable). Given a possible association between radiomics features and HPV status, inclusion of a radiomics score may effectively incorporate HPV status information.^50^ However, an incremental benefit to incorporating a radiomics score in addition to HPV status has also been suggested.^17^ The majority of patients in the development cohort in Cheng *et al*^16^ were HPV−, while more patients in the evaluation cohort were HPV+; there was however also a large proportion of participants with missing HPV status information in the evaluation cohort. The population included in Mes *et al*^21^ model was limited to HPV− patients and it is unclear how well the models would discriminate in a mixed HPV+/− population. Ward *et al* included neither HPV status nor a radiomics score, but AUCs for prediction of disease-specific survival were >0.8 in the development and external validation cohorts.^24^ This model included tumour-infiltrating lymphocytes. Models included in this review used different HPV diagnostics, which can affect the proportions of patients defined as HPV+. While median c-indices were similar between groups using either HPV, p16 or combined status (online supplemental material 5), there may be external validity issues when applying a model developed using one method of diagnosis to a population where another method of diagnosis has been used.

Most models included combinations of age, sex, T-stage and N-stage as model parameters. Beyond that there was variation in additional factors included. It is not possible to draw any conclusions on which combination of model parameters would produce the ‘best’ performing model as there are other factors that can influence model performance. These include population characteristics, event numbers, methods used to address missing data and modelling methods (eg, Cox regression vs machine learning). Reporting of these factors was variable, and sometimes poor, which also hampered a comprehensive assessment. Multicollinearity was poorly addressed in the included studies, with only one accounting for this in model development methods.^21^ Multicollinearity can be a problem in regression modelling leading to overfitting and poor model performance on external validation.^51^ This could be the case in those models including either T-stage, N-stage, M-stage or tumour volume as well as overall stage. Modelling techniques such as deep learning include techniques for feature selection and thus offer potential to mitigate multicollinearity and overfitting concerns.^52^

Four models included radiomics features^20 21^ or radiomics scores.^16 17^ However, the shortlisted radiomic features used in the modelling process were poorly documented, potentially impacting their wider usability. Additionally, radiomic features can display substantial heterogeneity and limited generalisability, depending on their derivation and processing methods, rendering direct comparisons of radiomics scores between studies a challenging task.

### Strengths and limitations

We believe this is the most comprehensive systematic review of models that include at least one clinical variable for predicting recurrence and survival in patients with treated OPC to date. Compared with previous systematic reviews,^8 9^ the review included a greater number of studies in patients with OPC; included only models that have been externally validated at least once; additionally included studies which were external validations of included models and included both recurrence and survival outcomes. Strengths of this review include a sensitive search strategy and including searches in the IEEE database, which may capture studies not reported in the more general medical databases. However, no additional relevant studies were found from searching IEEE. It is possible that studies may have been missed as full texts were only sought where an abstract mentioned a form of validation. However, large volumes of abstracts precluded further full-text checking and given the importance of validation, it is unlikely this aspect would have been omitted in an abstract. Reference checking would also have mitigated the risk of missing relevant studies. However, given the pragmatic decisions made during the study selection process and a small possibility of missing relevant models, additional searches could be performed before further work such as a head-to-head validation of all candidate models is conducted.

Inclusion of models was limited to those with at least one external validation. This decision was made because model performance is often overestimated with internal validation, hampering any conclusions that can be drawn. From a clinical point of view, models that are generalisable and suitable for implementation in practice are of most interest, but models should not be recommended before establishing external validity.^53^

A lack of external validation is a common problem in the predictive modelling landscape and many more models are developed than are externally validated.^53^ For the purposes of this systematic review, we have provided a list of excluded studies (online supplemental material 6) indicating where there was only internal validation. This list could be checked in the future to identify models that have had further external validation.

Overall review conclusions were hampered by poor reporting of details on model development and validation, which led to uncertainty around robustness of models. Contacting authors to obtain additional details could potentially have improved PROBAST scores, but may also have introduced further bias depending on completeness of responses. A lack of external validations also means there is uncertainty surrounding the generalisability of most models. Furthermore, the models developed by Cheng *et al*^16^ and Ma *et al*^20^ included in this review were based on machine learning and PROBAST may not be fully suitable for appraisal of this type of model. An artificial intelligence version, PROBAST-AI, is currently under development.^54^ Publication bias could not be formally assessed as no meta-analyses were undertaken.

### Unanswered questions and future research

Compared with other cancers, such as breast and prostate cancer, predictive modelling for less common cancers—including OPSCC, oral cavity, laryngeal, nasopharyngeal and hypopharyngeal cancer—is relatively underdeveloped and still some way from routine clinical implementation.^55^ For example, breast cancer has numerous well-established predictive models that have been developed and validated in large cohorts,^56^ including the PREDICT model,^57 58^ which is endorsed by the National Institute for Health and Care Excellence guidelines,^59^ and prostate cancer uses the European Association of Urology (EAU) risk group classification based on the D’Amico classification system,^60^ which is endorsed by EAU guidelines.^61^ In contrast, OPSCC modelling has lagged behind due to several factors. The rising incidence of HPV+ OPSCC over the past two to three decades has resulted in changing risk profiles and disease behaviour, making it challenging to develop comprehensive predictive models. Additionally, there are significant gaps in understanding the genomic profile of OPSCC, particularly within HPV+ cohorts, which show considerable heterogeneity in patient characteristics and outcomes. As a result, the field needs further research to develop and validate robust predictive models that can be widely implemented in clinical practice.

Models that have not been externally validated were not included in this review, and it is possible that there are existing models that have the potential to perform well. Such models, as well as the ones included in this review, could be further validated in independent, structurally different cohorts to increase confidence in their generalisability. Evaluating multiple models in the same patient cohort would also be useful in terms of enabling direct comparisons of model performance. We considered, but ruled out, a multivariate meta-analysis approach for comparing model performance as undertaken in the study by Usher-Smith *et al* as evaluation of different models in the same cohort was only undertaken in two studies, and transferability assumptions were unlikely to be met.^62^

Future research in outcome predictive modelling for patients with OPSCC should primarily focus on building methodologically robust models. Future studies should be large enough to ensure sufficient numbers of events (eg, ≥20 events per model variable for development studies)

^63^; should attempt to account for missing variable data rather than enrolling and analysing only those participants with complete data; should account for model overfitting and complexities of the data (such as competing risks) in the analysis and should report calibration as well as discrimination measures, as well as sufficient information on the method of outcome assessment (eg, for recurrence). The PROBAST tool^12 63^ can be used to identify common areas where model development or validation is likely to be flawed, while the TRIPOD statement should be used to improve reporting.^45^

The intended target population should be clearly described. HPV-associated and HPV− tumours are considered by many as two very distinct diseases on multiple levels: molecular, epidemiological, behavioural and clinical outcomes. Clinical prediction models trained on patients with OPSCC without factoring HPV status are therefore considered methodologically flawed, and their use in routine clinical practice should not be recommended. Moreover, there is no evidence in the literature to support the use of clinical prediction models trained on HPV-associated patients, for HPV− ones, or vice versa. Arguably, efforts for modelling outcomes for patients with OPSCC should try to create two distinct models/modelling processes for HPV-associated and HPV− patients to ensure model representativeness and generalisability. Such models are more likely to capture the impact of factors like patients’ age or smoking status for example, on disease outcomes and survival. This is particularly relevant as some factors may differ in their prognostic impact on HPV-associated HNC compared with HPV− HNC. Smoking, sex and overall cancer stage are known to be prognostic factors in HPV-associated HNC.^64^ Pathological extranodal extension has been shown to be a significant poor prognosticator in HPV− patients, while its impact on HPV-associated tumours remains controversial.^65^ Further research is still required on how HPV might modify other risk factors. Moreover, as HPV-associated disease has a very heterogenous geographic prevalence, separate HPV+ and HPV− models may be more practical for wider implementation. We acknowledge that including HPV status in a single model may be less of an issue with more advanced machine learning techniques (eg, ensemble methods or neural networks) as these have been reported to be able to factor in more complex relationships and dependencies in the data compared with regression methods.^66^ However, these have not been widely used in OPSCC modelling yet.

OS is the traditional choice of end point in cancer prognostication and has the advantage of not being a surrogate end point as well as being simple to measure, but is influenced by the competing risk of non-cancer deaths.^67^ Disease-specific measures such as PFS or EFS may be a more sensitive measure of treatment benefit compared with OS, particularly in younger and healthier HPV+ patients with expected long-term survival as well as providing more information on disease control and prevention of disease-related outcomes.

Finally, a plethora of novel variables are being explored, which may have a role in predicting outcomes in patients with OPSCC, such as molecular biomarker signatures, pathological variables such as circulating DNA as well as radiomics scores.^50 68 69^ It remains to be seen if these will retain their prognostic value when modelled with more routinely used clinical variables. Furthermore, their value in predicting outcomes when included in a model needs to be balanced against the resources needed to determine the variables as many require relatively advanced techniques and significant resource allocation, which may not be feasible in routine practice.

## Conclusion

Models mostly performed well in terms of discriminative ability (c-index >0.7), although none consistently showed a very good discriminative ability (c-index >0.8). Given the high RoB based on PROBAST assessment, it is uncertain how trustworthy these discriminative abilities are. Further external validation of existing models to assess generalisability should be limited to those models including HPV status as a variable. Development and validation of future models should be considered in HPV+ or HPV− cohorts separately to ensure model representativeness.

## supplementary material

10.1136/bmjopen-2024-090393 online supplemental file 1

10.1136/bmjopen-2024-090393 online supplemental file 2

10.1136/bmjopen-2024-090393 online supplemental file 3

10.1136/bmjopen-2024-090393 online supplemental file 4

10.1136/bmjopen-2024-090393 online supplemental file 5

10.1136/bmjopen-2024-090393 online supplemental file 6

10.1136/bmjopen-2024-090393 online supplemental file 7

10.1136/bmjopen-2024-090393 online supplemental file 8

10.1136/bmjopen-2024-090393 online supplemental file 9

10.1136/bmjopen-2024-090393 online supplemental file 10

## Data Availability

All data relevant to the study are included in the article or uploaded as supplementary information.
