# Supplementary material for: Systematic review of prognostic models for predicting recurrence and survival in patients with treated oropharyngeal cancer
Source: BMJ Open. 2024 Dec 5;14(12):e090393. doi: 10.1136/bmjopen-2024-090393 (PMC11624838; doi:10.1136/bmjopen-2024-090393)
Supplement: online supplemental file 2 [file bmjopen-14-12-s002.pdf]

Supplemental material 2

Search strategies

Embase

1 (head adj2 neck adj (cancer$ or carcinoma$ or neoplasm$ or mass$ or tumo?r$ or adenoma$ or lesion$ or metasta$ or malignan$ or nodule$)).ti,ab.

2 exp "head and neck tumor"/

3 ((oral cavity or pharyn$ or nasopharyn$ or oropharyn$ or hypopharyn$ or larynx or laryngeal or paranasal sinus$ or salivary gland or nasal cavity or oral cavity or tongue or lip or gingival or facial or mouth or tracheal or otorhinolaryngological) adj2 (cancer$ or carcinoma$ or neoplasm$ or mass$ or tumo?r$ or adenoma$ or lesion$ or metasta$ or malignan$ or nodule$)).ti,ab

4 (c-index or concordance index or c-statistic or concordance statistic or Nomogram$ or (predict$ adj3 model$) or (prognos$ adj3 model$) or (calibrat$ adj3 model$) or (discriminat$ adj3 model$) or Area under the curve or AUC or (Validat$ and (cohort$ or set$ or internal or external))).ti,ab.

5 1 or 2 or 3

6 exp cancer survival/

7 exp progression free survival/

8 exp overall survival/

9 exp cancer prognosis/

10 exp cancer recurrence/

11 exp disease free survival/

12 ((cancer$ or carcinoma$ or neoplasm$ or mass$ or tumo?r$ or adenoma$ or lesion$ or metasta$ or malignan$ or nodule$) adj5 (pfs or dfs or os or "progression free survival" or "disease free survival" or "overall survival")).ti,ab.

13 ((cancer$ or carcinoma$ or neoplasm$ or mass$ or tumo?r$ or adenoma$ or lesion$ or metasta$ or malignan$ or nodule$) adj5 (recur$ or relaps$ or regre$ or metastas$ or progres*)).ti,ab.

14 6 or 7 or 8 or 9 or 10 or 11 or 12 or 13

15 4 and 5 and 14

16 limit 15 to yr="2005 -Current"

MEDLINE and In-Process

1 (head adj2 neck adj (cancer$ or carcinoma$ or neoplasm$ or mass$ or tumo?r$ or adenoma$ or lesion$ or metasta$ or malignan$ or nodule$)).ti,ab.

2 exp "head and neck neoplasms"/

3 ((oral cavity or pharyn$ or nasopharyn$ or oropharyn$ or hypopharyn$ or larynx or laryngeal or paranasal sinus$ or salivary gland or nasal cavity or oral cavity or tongue or lip or gingival or facial or mouth or tracheal or otorhinolaryngological) adj2 (cancer$ or carcinoma$ or neoplasm$ or mass$ or tumo?r$ or adenoma$ or lesion$ or metasta$ or malignan$ or nodule$)).ti,ab.

4 (c-index or concordance index or c-statistic or concordance statistic or Nomogram? or nomogram, or (predict$ adj3 model$) or (prognos$ adj3 model$) or (calibrat$ adj3 model$) or (discriminat$ adj3 model$) or Area under the curve or AUC or (Validat$ and (cohort$ or set$ or internal or external))).ti,ab.

5 1 or 2 or 3

6 exp cancer survivors/

7 exp progression-free survival/

8 exp survival/

9 exp prognosis/

10 exp neoplasm recurrence, local/

11 exp disease-free survival/

12 ((cancer$ or carcinoma$ or neoplasm$ or mass$ or tumo?r$ or adenoma$ or lesion$ or metasta$ or malignan$ or nodule$) adj5 (pfs or dfs or os or "progression free survival" or "disease free survival" or "overall survival")).ti,ab.

13 ((cancer$ or carcinoma$ or neoplasm$ or mass$ or tumo?r$ or adenoma$ or lesion$ or metasta$ or malignan$ or nodule$) adj5 (recur$ or relaps$ or regre$ or metastas$ or progres*)).ti,ab.

14 6 or 7 or 8 or 9 or 10 or 11 or 12 or 13

15 4 and 5 and 14

16 limit 15 to yr="2005 -Current" (2856)

IEEE database

Search 1

"head and neck" OR "oral cavity" OR pharyn* OR nasopharyn* OR hypopharyng*

AND

cancer OR tumour* OR tumour* OR carcinoma* OR metastas* OR malignancies OR malignancy

AND

model OR "c-index" OR "c-statistic" OR nomogram*

Search 2

larynx OR laryngeal OR "paranasal sinus*" OR "salivary gland" OR "nasal cavity" OR "oral cavity" OR tongue OR lip OR gingiva OR facial OR mouth OR tracheal OR otorhinolaryngological

AND

cancer OR tumour* OR tumour* OR carcinoma* OR metastas* OR malignancies OR malignancy

AND

model OR "c-index" OR "c-statistic" OR nomogram*

Search 3

larynx OR laryngeal OR "paranasal sinus*" OR "salivary gland" OR "nasal cavity" OR "oral cavity" OR tongue OR lip OR gingiva OR facial OR mouth OR tracheal OR otorhinolaryngological

AND

neoplasm OR mass OR nodule OR adenoma OR lesion

AND

"concordance index" OR "area under the curve" OR calibration OR validation

Search 4

"head and neck" OR "oral cavity" OR pharyn* OR nasopharyn* OR hypopharyng*

AND

neoplasm OR mass OR nodule OR adenoma OR lesion

AND

"concordance index" OR "area under the curve" OR calibration OR validation
