# Supplementary material for: Systematic review of prognostic models for predicting recurrence and survival in patients with treated oropharyngeal cancer
Source: BMJ Open. 2024 Dec 5;14(12):e090393. doi: 10.1136/bmjopen-2024-090393 (PMC11624838; doi:10.1136/bmjopen-2024-090393)
Supplement: online supplemental file 3 [file bmjopen-14-12-s003.pdf]

Supplementary Material 3

Study selection criteria

**POPULATION**
• Adults (≥18 years) with OPC who have completed treatment with curative intent.
**INDEX AND COMPARATOR MODELS**
• Models that combined two or more prognostic factors in a statistical model to predict an individual's risk of the outcome.<br>• Models that included one or more clinical variables (e.g. age, sex, tumour staging parameters, smoking/alcohol consumption, HPV status) with or without additional genetic or radiomics variables.<br><br>*Models with only genetic or radiomics variables were excluded.*
**OUTCOME**
• Any recurrence or survival related outcomes (e.g. recurrence-free survival, overall survival).
**TIMING**
• Models to be applied after treatment with curative intent; any time horizon for outcome prediction.
**SETTING/ROLE**
• Risk stratification in secondary care.
**STUDY DESIGN**
• Studies of models which were externally validated at least once regardless of whether they were internally validated.<br>• External validation was defined as validation in a separate patient cohort from a different institution/database than the development cohort.<br>• Development (only) studies where the model was externally validated in a separate study.<br>• Studies reporting impact assessment of a prognostic model.<br><br>*Models that were internally validated only were excluded.*
