# Supplementary material for: Systematic review of prognostic models for predicting recurrence and survival in patients with treated oropharyngeal cancer
Source: BMJ Open. 2024 Dec 5;14(12):e090393. doi: 10.1136/bmjopen-2024-090393 (PMC11624838; doi:10.1136/bmjopen-2024-090393)
Supplement: online supplemental file 4 [file bmjopen-14-12-s004.pdf]

Supplemental material 4

Criteria for risk of bias decisions (PROBAST)

**Risk of bias (PROBAST criteria)**	
Y=yes, PY=probably yes, N=no, PN=probably no, NI=no information, NEI=not enough information	
**Appropriate data sources?**	Y if prospective cohort with consecutive patients or ALL patients, or RCT.<br>PY if prospective cohort (no further details) or retrospective analysis with consecutive patients (or all patient admitted during specified time<br>period).<br>NEI retrospective analysis with no further details or if states single centre with no further information.
**Were all predictors defined and assessed in similar way for all participants?**	Y if reference to standard criteria used in all patients.<br>PY if states that assessments performed in accordance with relevant guidelines/regulations, or if RCT.<br>PY if single centre.<br><br>PY if multicentre but standardised protocol.<br>N- if details stating this was done in different ways across participants or cohorts.<br><br>NB rated as PN/N if one or more predictors not assessed in same way.
**Were predictor assessments made without knowledge of outcome data?**	Y if clear that all score components measured before outcome (e.g. in prospective study).<br>Y if retrospective, with statement that parameters assessed blindly with no knowledge of outcome, and/or clear that all details taken from existing notes/records.<br>PY if retrospective and parameters likely to have been measured before outcomes occurred.
**Are all predictors available at the time the model is intended to be used?**	All likely to be available if model for use after treatment, so always scored as Y.
**Was the outcome determined appropriately?**	For OS always Y.<br><br>Likely to be NI, NEI for anything recurrence related as mostly no information given and may be differences in FU/ascertainment.

**Risk of bias (PROBAST criteria)**	
Y=yes, PY=probably yes, N=no, PN=probably no, NI=no information, NEI=not enough information	
**Was a pre-specified or standard outcome definition used?**	Y- if a definition given, NI if none
**Were predictors excluded from the outcome definition?**	Always scored as Y.
**Was the outcome defined and determined in a similar way for all participants?**	For OS always Y.<br><br>Likely to be NI, NEI for anything recurrence related as may be differences in FU/ascertainment (e.g. all biopsy or all radiology + MDT etc.).<br><br>N if statement that there was a difference in determining outcome.
**Was the outcome determined without knowledge of predictor information?**	Y-if statement on blinding; likely to be NI.
**Was the time interval between predictor assessment and outcome determination appropriate?**	Y-if 2+ years for all patients; PY if likely 2+ years for most patients based on mean/median; NI-if no details on length of FU; PN -if mean/median <2 years; PN if median and lower range below 2 yrs; N if <2 years for all patients.<br><br>NB consider if follow-up is consistent with outcome measure, e.g. 5-year survival or 2-year survival. Rate as PN/N if mean/median substantially less than survival outcome presented.
**Was there a reasonable number of participants with the outcome?**	Development: >20 (or at least >10) events per variable for candidate predictors (if number of candidate predictors not known assume at least as many as final number of predictors included).<br>Validation: at least 100 participants with outcome.

Risk of bias (PROBAST criteria)	
Y=yes, PY=probably yes, N=no, PN=probably no, NI=no information, NEI=not enough information	
**Were continuous and categorical predictors handled appropriately?**	Development:<br>N: High risk of bias if dichotomised continuous predictors included. For model development studies that have dichotomized continuous predictors after data analysis and did not adjust for this by applying internal validation and shrinkage techniques, this signalling question should be answered as N.<br><br>PY: If cut-off predefined (widely accepted) rather than based on the data. If 4 or more categories rather than dichotomisation (especially if based on widely accepted cut-offs for the categories). Cut-points should ideally be established based on larger cohort/population, not data driven, i.e. based on data analysis (ROC curve analysis) of development cohort.<br><br>Validation:<br>Should be using model as originally fitted-same dichotomisation and cut-offs. Using equation/model as created.
**Were all enrolled participants included in the analysis?**	N if not including all eligible participants, e.g. if including on basis of available parameter data or outcome information. Mostly NI.
**Were participants with missing data handled appropriately?**	N: if participants with missing data excluded.<br>Y: Should use multiple imputation. (Not missing indicator method). Look for model performance with and without missing participants -if similar then less likely to be biased.<br>N/A -if no missing data<br>If stated that included and excluded participants similar, then still N for this Q.
**Was selection of predictors based on univariate analysis avoided? (DEV only)**	When predictors are selected on the basis of univariable analysis before multivariable modelling, the signalling question for these studies should be answered as N.<br>More appropriate to choose candidate predictors (for inclusion into multivariable modelling) based on existing knowledge/previously established predictors/clinical credibility. Include all candidate variables in model not just those that were significant on univariate analysis.
**Were complexities in the data (e.g. censoring, competing risks, sampling of control**	Likely to be NI for this. Should account for competing risk, as up to 50% of deaths not due to H&NC.

**Risk of bias (PROBAST criteria)**	
Y=yes, PY=probably yes, N=no, PN=probably no, NI=no information, NEI=not enough information	
**participants) accounted for appropriately?**	
**Were relevant model performance measures evaluated appropriately?**	Should be both discrimination (C-statistic) and calibration statistics. Calibration plot or table with O/E. Statistical test for calibration may not be enough on its own (Hosmer-Lemeshow test). If classification (sens/spec) and reclassification measures presented without model calibration also insufficient.
**Were model overfitting and optimism in model performance accounted for? (DEV only)**	Studies developing prediction models should always include some form of internal validation, such as bootstrapping and cross-validation. Internal validation is important to quantify overfitting of the developed model and optimism in its predictive performance, except when sample size and EPV are extremely large. If optimism is present, an important further step is to adjust or shrink the model predictive performance estimates (such as c-index) and predictor effects in the final model.<br><br>Researchers often randomly split a data set at the participant level into 2 groups (1 for model development and 1 for internal validation), which has been shown to be an inadequate way to measure optimism. (Though better if have high number of events per candidate variable) Also, researchers often apply bootstrapping and cross-validation techniques to examine optimism but fail to replicate the exact model development procedure (for example, predictor selection procedures, in both univariable and multivariable analysis) and thus may underestimate the actual optimism for their model. Such inappropriate methods would lead to an N for this signalling question.
**Do predictors and their assigned weights in the final model correspond to the results from the reported multivariable analysis? (DEV only)**	Predictors and coefficients of the final developed model, including intercept or baseline components, should be fully reported to allow others to correctly apply the model to other individuals. Ideally want to see formula.<br>Even if have formula with coefficients, are looking for how translated into score/mapped to score (i.e. how do allocate certain number of points for presence/absence of a risk factor). Predictors scaled to magnitude of coefficients?
