# Supplementary material for: Systematic review of prognostic models for predicting recurrence and survival in patients with treated oropharyngeal cancer
Source: BMJ Open. 2024 Dec 5;14(12):e090393. doi: 10.1136/bmjopen-2024-090393 (PMC11624838; doi:10.1136/bmjopen-2024-090393)
Supplement: online supplemental file 5 [file bmjopen-14-12-s005.pdf]

Supplemental material 5

Model performance individualised prediction models

<table>
<tr><th>Study

(DEV or EV)</th><th>Model parameters and cut-offs; algorithm, formula, nomogram or online risk calculator available?</th><th>Model performance measures</th></tr>
<tr><td colspan="3">Individualised prediction models</td></tr>
<tr><td>Fakhry 2017, US

Development</td><td rowspan="3">Fakhry 2017 nomogram for OS

Age: ≤50yrs vs > 50 yrs

Zubrod performance status: 0 vs 1

Pack-years: ≤ 10 PYs vs > 10 PYs

Education: high school or less vs others

p16 status: p16+ vs p16-

T stage: T4 vs T2-3

N stage: TNM7 N2c-3 vs N0-2b, TNM8 N2-N3 Vs N0-N1

Anaemia: yes vs no (defined as haemoglobin level ≤13.5 g/dL</td><td>Model discrimination (c-index): 0.76 (95% CI 0.72, 0.80); after 20-fold cross-validation: 0.74 (95% CI 0.70, 0.78); after 10-fold cross-validation: 0.75 (95% CI 0.71, 0.79) NB unclear if 2 or 5 yr OS

Model calibration: Observed event rate for OS was similar to that predicted in the model, with slightly higher observed (compared with predicted) survival in patients with poorer predicted probability of survival. Other model performance measures: NR</td></tr>
<tr><td>Fakhry 2017, US

External validation</td><td>Model discrimination (c-index): uncorrected 0.68 (95% CI 0.63, 0.73); bootstrap corrected: 0.64 (95% CI 0.59, 0.69) NB unclear if 2 or 5 yr OS

Model calibration: NR

Other model performance measures: NR</td></tr>
<tr><td>Bossi 2018

External validation</td><td>Model discrimination (c-index): OS: c-index for 2 and 5-yr OS 0.779 (95% CI 0.683-0.875)

Model calibration: Agreement between predicted and observed outcomes was poor, with observed survival consistently higher than predicted at both 2 and 5 years.

Other model performance measures: NR</td></tr>
</table>

**Study**<br><br>**(DEV or EV)**	**Model parameters and cut-offs; algorithm, formula, nomogram or online risk calculator available?**	**Model performance measures**
Beesley 2019, US<br><br>*External validation*	for men and ≤12.5 g/dL for women)<br><br><u>Age x pack-years interaction</u><br><br>https://www.nrgoncology.org/Meetings-Resources/Nomograms-For-Prediction-of-Survival/Oropharynx-Cancer-Overall-Survival-Calculator	*Model discrimination (c-index)*: 5yr OS c-index 0.73 (no CI), 5yr OS AUC 0.77 (no CI)<br><br>*Model calibration*: Observed 5-year OS was lower compared with predicted in the model for intermediate predicted probabilities, but was higher than predicted for some of the lower predicted probabilities.<br><br>*Other model performance measures*: NR
Beesley 2021, US<br><br>*External validation*		*Model discrimination (c-index)*: 5yr OS c-index 0.74 (no CI), 5yr OS AUC 0.73 (no CI)<br><br>*Model calibration*: NR<br><br>*Other model performance measures*: NR
Nelson 2022<br><br>*External validation*		*Model discrimination (c-index)*: OS: whole cohort c-index 0.67 (SE 0.008), complete data c-index 0.72 (SE 0.027). (NB *these figures for 'multivariable' model from table 2 are different from figures quoted in the manuscript (which are the univariable model's)).*<br><br>*Model calibration*: In the whole cohort, observed overall survival was lower at 2 and 5 years compared with predicted, except for where 2-year predicted probability OS was 0.9 , which showed similar observed OS. In complete cases only, observed OS and predicted OS were similar for higher predicted probabilities of OS at both 2 and 5 years. However, the observed 5-year OS was higher than predicted for patients with lower predicted probability of OS.<br><br>*Other model performance measures*: NR<br><br>NB some model variables defined differently: smoking (**previous/current VS never**), PS (0 vs **≥1**), T-stage (T4 vs **T1-3**), education level (**percent with high school diploma by zip code <sample median [87.7%] vs ≥sample median**).

<table>
<tr><th>Study

(DEV or EV)</th><th>Model parameters and cut-offs; algorithm, formula, nomogram or online risk calculator available?</th><th>Model performance measures</th></tr>
<tr><td>Fakhry 2017, US

Development</td><td rowspan="3">Fakhry 2017 nomogram for PFS

<u>Age</u>: ≤50yrs vs > 50 yrs

<u>Zubrod performance status</u>: 0 vs 1

<u>Pack-years</u>: ≤ 10 PYs vs > 10 PYs

<u>Education</u>: high school or less vs others

<u>p16 status</u>: p16+ vs p16-

<u>T stage</u>: T4 vs T2-3

<u>N stage</u>: TNM7 N2c-3 vs N0-2b, TNM8 N2-N3 Vs N0-N1

<u>Marital status</u>: married or with live-in partner vs</td><td>Model discrimination (c-index): 0.70 (95% CI 0.66, 0.74); after 20-fold cross-validation: 0.69 (95% CI 0.65, 0.73); after 10-fold cross-validation: 0.68 (95% CI 0.64, 0.72) NB unclear if 2 or 5 yr OS

Model calibration: Observed event rate for PFS was similar to that predicted in the model.

Other model performance measures: NR</td></tr>
<tr><td>Fakhry 2017, US

External validation</td><td>Model discrimination (c-index): uncorrected 0.68 (95% CI 0.64, 0.72); bootstrap corrected: 0.64 (95% CI 0.60, 0.68) NB unclear if 2 or 5 yr OS

Model calibration: NR

Other model performance measures: NR</td></tr>
<tr><td>Bossi 2018

External validation</td><td>Model discrimination (c-index): PFS: c-Index 0.721 (95% CI 0.651-0.791) and 0.720 (95% CI 0.650-0.790) for 2- and 5-year PFS respectively

Model calibration: Agreement between predicted and observed outcome was poor, with progression-free survival consistently higher than predicted at both 2 and 5 years.

Other model performance measures: NR</td></tr>
</table>

**Study**<br><br>**(DEV or EV)**	**Model parameters and cut-offs; algorithm, formula, nomogram or online risk calculator available?**	**Model performance measures**
Nelson 2022<br><br>*External validation*	single/divorced/separated/widowed<br><br><u>Weight loss in last 6 months</u>: ≥ 5% vs < 5%<br><br><u>p16 x Zubrod interaction</u><br><br>https://www.nrgoncology.org/Meetings-Resources/Nomograms-For-Prediction-of-Survival/Oropharynx-Cancer-Progression-Free-Survival-Calculator	*Model discrimination (c-index)*: PFS: whole cohort c-index 0.66 (SE 0.0074), complete data c-index 0.68 (SE 0.025). (NB *these figures for 'multivariable' model from table 2 are different from figures quoted in the manuscript (which are the univariable model's)).*<br><br>*Model calibration*: In the whole cohort, observed progression free survival was lower at 2 and 5-years compared with predicted. In complete cases only, observed PFS at 2 and 5 years was slightly lower, but with observed PFS at 5 years higher than predicted for patients with lower predicted probability of PFS (around 0.35).<br><br>*Other model performance measures*: NR<br><br>NB some model variables defined differently: smoking (**previous/current VS never**), PS (0 vs **≥1**), , T-stage (T4 vs **T1-3**), education level (**percent with high school diploma by zip code <sample median [87.7%] vs ≥sample median**), **body mass index (BMI) (<20 vs ≥20**).
Grønhøj-Larsen 2016<br><br>*Development*	**Grønhøj-Larsen 2016 OS nomogram**<br><br><u>Treatment</u>: CT, RT, palliative, no treatment<br><br><u>Age</u>: continuous scale<br><br><u>HPV/p16 status</u>: HPV+/p16+, HPV+/p16-, HPV-/p16+, HPV-/p16-<br><br><u>T-stage</u>:T1, T2, T3, T4	*Model discrimination (c-index)*: 5yr OS c-index 0.79 (no CI, internally validated)<br><br>*Model calibration*: Observed and predicted 2- and 5-year survival were reasonably similar, with slight overprediction of 5-year overall survival for patients with intermediate predicted probability of survival.<br><br>*Other model performance measures*: NR
Beesley 2019, US<br><br>*External validation*		*Model discrimination (c-index)*: 5yr OS c-index 0.78 (no CI), AUC 0.80 (no CI)<br><br>*Model calibration*: Observed and predicted 5-year survival were similar.<br><br>*Other model performance measures*: NR

Study<br><br>(DEV or EV)	Model parameters and cut-offs; algorithm, formula, nomogram or online risk calculator available?	Model performance measures
Beesley 2021, US<br><br>*External validation*	<u>N-stage</u>: N0, N1, N2, N3<br><br><u>Pack years</u>: continuous scale<br><br><u>Performance status</u>: 0, 1,2,3,4<br><br>*NB A time to progression nomogram also developed based on HPV/p16 status, N-stage and pack years, but this has not been externally validated.*	*Model discrimination (c-index)*: 5yr OS c-index 0.77 (no CI), 5yr OS AUC 0.78 (no CI)<br><br>*Model calibration*: NR<br><br>*Other model performance measures*: NR
Grønhøj 2018, Denmark<br><br>*Development*	**OroGrams nomogram**<br><br><u>Sex</u>: female (reference) vs male<br><br><u>Age</u>: HR of event with increasing age <u>Smoking</u>: current smoker (reference) vs former smoker vs never smoked(0PY)<br><br><u>HPV status</u>: HPV-/P16- (reference) vs HPV-/P16+ vs HPV+/P16- vs HPV-/P16-<br><br><u>T-stage</u>: T1 (reference) vs T2 vs T3 vs T4	*Model discrimination (c-index)*: OS: c-statistic 0.787 (95% CI 0.753–0.817), 0.772 (95% CI 0.747–0.817) and 0.766 (95% CI 0.746–0.788) for 1, 3 and 5-year OS respectively. PFS: c-statistic 0.733 (95% CI 0.703–0.760), 0.728 (95% CI 0.704–0.750) and 0.725 (95% CI 0.703–0.747) for 1, 3 and 5-year PFS respectively.<br><br>*Model calibration*: Observed and predicted survival were reasonably similar at 1, 3 and 5 years, with slightly lower observed survival (compared with predicted) for patients with intermediate predicted probability of survival.<br><br>*Other model performance measures*: Across the four cohorts, Brier scores were roughly (estimated from graph) between 0.07 and 0.15 at 1 year, between 0.13 and 0.2 at 3 years and between 0.16 and 0.2 at 5 years. Model performance decreases over time. Model performance is slightly worse in the German cohort (EV1) for both OS and PFS (compared with the DEV cohort) and slightly better in the UK and Swedish cohorts (EV2 and EV3). The difference in model performance between cohorts is not as pronounced at later follow-up times.

**Study**<br><br>**(DEV or EV)**	**Model parameters and cut-offs; algorithm, formula, nomogram or online risk calculator available?**	**Model performance measures**
Grønhøj 2018, Denmark<br><br>*External validation 1 (Germany)*	N-stage: N0 ref vs N1 Vs N2 vs N3<br><br>M-stage: M0 ref vs M1<br><br>UICC8 stage: ***only for OS model*** (Stage I ref vs stage II vs stage III vs stage IV)<br><br>*Nomogram presented and online tool* *www.orograms.org*	*Model discrimination (c-index)*: OS: c-statistic 0.712 (95% CI 0.655–0.764), 0.722 (95% CI 0.683–0.759) and 0.707 (95% CI 0.671–0.741) for 1, 3 and 5-year OS respectively.<br><br>PFS Model: c-statistic 0.714 (95% CI 0.663–0.761), 0.711 (95% CI 0.671–0.748) and 0.704 (95% CI 0.667–0.738) for 1, 3 and 5-years PFS respectively.<br><br>*Model calibration*: Observed survival was frequently higher than predicted survival across all time points apart from where 3-year predicted survival was roughly between 20% and 35%; here observed survival was slightly lower than predicted survival at 3 years.<br><br>*Other model performance measures*: See DEV cohort
Grønhøj 2018, Denmark<br><br>*External validation 2 (Sweden)*		*Model discrimination (c-index)*: OS: c-statistic 0.836 (95% CI 0.775–0.881), 0.793 (95% CI 0.749–0.833) and 0.780 (95% CI 0.743–0.815); for 1, 3 and 5-year OS respectively.<br><br>PFS Model: c-statistic 0.805 (95% CI 0.745–0.852), 0.763 (95% CI 0.722–0.802) and 0.764 (95% CI 0.724–0.801) for 1, 3 and 5-year PFS respectively.<br><br>*Model calibration*: Observed survival was frequently higher than predicted survival across all time points apart from where 1-year predicted survival was roughly between 60% and 75%; here observed survival was lower than predicted survival.<br><br>*Other model performance measures*: See DEV cohort
Grønhøj 2018, Denmark<br><br>*External validation 3 (UK)*		*Model discrimination (c-index)*: OS: c-statistic 0.815 (95% CI 0.775–0.864), 0.797 (95% CI 0.755–0.832) and 0.791 (95% CI 0.751–0.822). for 1, 3 and 5-year OS respectively.<br><br>PFS: c-statistic 0.797 (95% CI 0.739–0.842), 0.778 (95% CI 0.735–0.812) and 0.771 (95% CI 0.731–0.805) for 1, 3 and 5-year PFS respectively.

Study<br><br>(DEV or EV)	Model parameters and cut-offs; algorithm, formula, nomogram or online risk calculator available?	Model performance measures
		Model calibration: Observed and predicted survival were reasonably similar at 1 year. Three year predicted survival was lower than observed survival across all predicted survivals apart from where predicted survival was roughly between 60% and 75%. Here, 3-year predicted survival was slightly higher than observed survival. Higher or lower observed versus predicted survival at 5-years depending varied dependent on predicted survival risk.<br><br>*Other model performance measures*: See DEV cohort
Mentel 2021, UK<br><br>*External validation*		*Model discrimination (c-index)*: NR<br><br>*Model calibration*: NR<br><br>*Other model performance measures*: Brier scores below 0.20 at 1, 3 and 5 years suggest good predictive ability for both OS and PFS, with slightly better predictive ability for OS. Model performance decreased over time. Lower Brier scores in an HPV+ sub-group indicate better module performance in this group.
Rios-Velazquez 2014, The Netherlands<br><br>*Development*	**Rios-Velazquez 2014 nomogram**<br><br><u>HPV status</u>: HPV DNA positive or negative<br><br><u>Smoking</u>: none, moderate (1–30 PYs) and heavy (>30 PYs)	*Model discrimination (c-index)*: OS: c-statistic 0.82 (95% CI 0.76, 0.88); PFS: c-statistic 0.80 (95% CI 0.76, 0.88)<br><br>*Model calibration*: NR<br><br>*Other model performance measures*: NR
Rios-Velazquez 2014, The Netherlands		*Model discrimination (c-index)*: OS: c-statistic 0.73 (95% CI 0.66, 0.79); PFS: c-statistic 0.67 (95% CI 0.59, 0.74)

**Study**<br><br>**(DEV or EV)**	**Model parameters and cut-offs; algorithm, formula, nomogram or online risk calculator available?**	**Model performance measures**
External validation	<u>Patient comorbidity</u>: ACE27 0-1 vs 2-3<br><br>Pre-treatment<br><br><u>Haemoglobin levels</u>: continuous scale<br><br><u>Sex</u>: male vs female<br><br><u>T stage</u>: T1, T2, T3, T4<br><br><u>N stage</u>: N0-N2a Vs N2b-N3<br><br>Nomogram and online tool www.predictcancer.org	*Model calibration*: NR<br><br>*Other model performance measures*: NR
Beesley 2019<br><br>*External validation*		*Model discrimination (c-index)*: 5yr OS: c-index 0.71, AUC: 0.74<br><br>*Model calibration*: Observed 5-year survival was higher compared with predicted 5-year survival, especially in patients with lower predicted probability of survival.<br><br>*Other model performance measures*: NR
Beesley 2021, US/The Netherlands<br><br>*Development*	**Beesley 2021 clinical model**<br><br><u>Age at diagnosis</u>: cut-off unclear<br><br><u>Sex</u>: male vs female<br><br><u>ACE 27 score</u>: none vs mild vs moderate vs severe<br><br><u>Smoking</u>: never vs former vs current in last 12 months	*Model discrimination (c-index)*: 5yr OS: c-index 0.76 (no CI), AUC: 0.78 (no CI); 5yr event-free-survival: c-index 0.73 (no CI), AUC 0.76 (no CI)<br><br>*Model calibration*: The observed and predicted probabilities of EFS look reasonably similar over time for all strata (strata defined by cT classification and p16 status).<br><br>The probability of EFS predicted from the model is slightly lower than observed for later time points (>= 50 months) in A,E,F strata (cT1, p16:missing, p16:negative). The predicted probability of EFS for early time points (<10 months) is higher than observed in the E stratum (p16:missing). The predicted probability of PFS for early time points (<10 months) is lower than observed in the F stratum (p16: negative).

Study<br><br>(DEV or EV)	Model parameters and cut-offs; algorithm, formula, nomogram or online risk calculator available?	Model performance measures
	Anaemia: haemoglobin level less than 12 g/dL for women and 13 g/dL for men<br><br>p-16 status: positive or negative<br><br>T-stage (8th ed): T1 vs T2 vs T3 vs T4<br><br>N-stage (8th ed): N0 vs N1 vs N2abc vs N3<br><br>*Web tool (calculator) http://*<br><br>*shiny.sph.umich.edu/Oropharynx_Calc/.*	*Other model performance measures*: NR
Beesley 2021, US/The Netherlands<br><br>*External validation*		*Model discrimination (c-index)*: 5yr OS AUC 0.75 (no CI) and C-index 0.70 (no CI), 5yr event-free-survival AUC 0.72 and c-Index 0.69.<br><br>*Model calibration*: Observed and predicted 5-year overall and event-free survival were reasonably similar, with slightly lower observed than predicted overall survival in patients with lower predicted probability of OS and higher predicted EFS. The observed OS and EFS was lower than predicted for patients with high (above around 0.75) predicted probability of OS.<br><br>*Other model performance measures*: NR<br><br>*Also developed a model with additional parameters (MTV and radiologic extracapsular extension) but this was not externally validated.*
Cheng 2021, Taiwan<br><br>*Development*	**Cheng 2021 models**<br><br>Clinical + radiomics model (with HPV, 'Integrated model')<br><br>Age: continuous scale<br><br>Sex: male vs female<br><br>cT stage: T1 vs T2 vs T3 vs T4a vs T4b)	*Model discrimination (c-index)*: 5-year OS: AUCs 0.793 (95% CI, 0.749–0.834); c-Index: 0.757 (95% CI 0·714-0·800); 2yr-OS: AUC 0.804 (95% CI 0.758-0.848).<br><br>*Model calibration*: The observed event rate for overall survival was similar to that predicted in the model for 2-year and 5-year OS, with predictions for 2-year OS performing slightly better than 5-year OS predictions.<br><br>*Other model performance measures*: NR
Cheng 2021, Taiwan<br><br>*External validation 1 (six centres*		*Model discrimination (c-index)*: 5-year OS: AUC 0.801 (95% CI, 0.727–0.874); c-Index 0.792 (95% CI 0·720-0·865); 2yr-OS: AUC 0.867 (95% CI 0.797-0.931)

**Study**<br><br>**(DEV or EV)**	**Model parameters and cut-offs; algorithm, formula, nomogram or online risk calculator available?**	**Model performance measures**
(Canada, US, Netherlands) from TCIA database)	cN stage: N0 vs N1 vs N2 vs N3<br><br>Clinical TNM stage: I vs II vs III vs Iva vs IVb)<br><br>HPV: positive vs negative<br><br>DeepPET-OPSCC score: continuous scale<br><br>*Nomogram presented (in supplementary material) and software package that can be installed in radiology suites. Links provided to repositories, which show major components of the tool and illustrative examples.*	*Model calibration*: The observed event rate for OS in the external validation set was slightly lower than predicted in the model for predicted probabilities less than about 0.90 for both 2-year OS and 5-year OS, and slightly higher than predicted for the highest predicted probabilities. Low predicted or observed event rates of OS did not occur in the external validation set, so no calibration slope is shown for lower values.<br><br>*Other model performance measures*: NR
Cheng 2021, Taiwan<br><br>*External validation 2 (two hospitals, China)*		*Model discrimination (c-index)*: c-index 0.787 (95% CI, 0.675–0.899)<br><br>*Model calibration*: NR<br><br>*Other model performance measures*: NR
Cheng 2021, Taiwan<br><br>*Development*	**Cheng 2021 models**<br><br>Clinical model (with HPV)<br><br>Age: continuous scale<br><br>Sex: male vs female<br><br>cT stage: T1 vs T2 vs T3 vs T4a vs T4b)<br><br>cN stage: N0 vs N1 vs N2 vs N3	*Model discrimination (c-index)*: 5yr OS: AUC 0.768 (0.722-0.813); c-index: 0.726 (0.683-0.769); 2yr OS: AUC 0.778 (0.727-0.823)<br><br>*Model calibration*: NR<br><br>*Other model performance measures*: NR
Cheng 2021, Taiwan<br><br>*External validation 1 (six centres (Canada, US,*		*Model discrimination (c-index)*: 5yr OS: 0.749 (0.649-0.842); c-index: 0.768 (0.694-0.842); 2yr OS: AUC 0.846 (0.775-0.912)<br><br>*Model calibration*: NR

**Study**<br><br>**(DEV or EV)**	**Model parameters and cut-offs; algorithm, formula, nomogram or online risk calculator available?**	**Model performance measures**
Netherlands) from TCIA database)	<u>Clinical TNM stage</u>: I vs II vs III vs Iva vs IVb)<br><br><u>HPV</u>: positive vs negative	*Other model performance measures*: NR
Cheng 2021, Taiwan<br><br>*Development*	**Cheng 2021 models**<br><br>Clinical + radiomics model (without HPV)<br><br><u>Age</u>: continuous scale<br><br><u>Sex</u>: male vs female<br><br><u>cT stage</u>: T1 vs T2 vs T3 vs T4a vs T4b)<br><br><u>cN stage</u>: N0 vs N1 vs N2 vs N3<br><br><u>Clinical TNM stage</u>: I vs II vs III vs Iva vs IVb)<br><br><u>DeepPET-OPSCC score</u>: continuous scale	*Model discrimination (c-index)*: 5 yr OS: AUC 0.755 (0.706-0.801); c-index 0.731 (0.684-0.779); 2yr AUC 0.772 (0.718-0.823)<br><br>*Model calibration*: The observed event rate for OS was slightly lower than predicted in the model for 5-year OS (at predicted probabilities less than about 45%) and slightly higher than predicted at predicted probabilities of above 55%. Observed and predicted event rates for 2-year survival were similar.<br><br>*Other model performance measures*: NR
Cheng 2021, Taiwan<br><br>*External validation 1 (six centres (Canada, US, Netherlands) from TCIA database)*		*Model discrimination (c-index)*: 5-year OS: AUC 0.727 (95% CI 0.671-0.780); c-index 0·712 (95% CI 0·646-0·777); 2yr OS AUC 0.754 (0.659-0.841)<br><br>*Model calibration*: The observed event rate for OS in the external validation set was slightly lower than predicted in the model for predicted probabilities less than about 0.85 for both 2-year OS and 5-year OS, and slightly higher than predicted for the highest predicted probabilities. Low predicted or observed event rates of OS did not occur in the external validation set, so no calibration slope is shown for lower values.<br><br>*Other model performance measures*: NR
Cheng 2021, Taiwan	**Cheng 2021 models**	*Model discrimination (c-index)*: 5yr OS: AUC 0.713 (0.662-0.767); c-index: 0.684 (0.640-0.729); 2yr OS: AUC 0.734 (0.678-0.788)

**Study**<br><br>**(DEV or EV)**	**Model parameters and cut-offs; algorithm, formula, nomogram or online risk calculator available?**	**Model performance measures**
Development	Clinical model (without HPV)<br><br><u>Age</u>: continuous scale<br><br><u>Sex</u>: male vs female<br><br><u>cT stage</u>: T1 vs T2 vs T3 vs T4a vs T4b)<br><br><u>cN stage</u>: N0 vs N1 vs N2 vs N3<br><br><u>Clinical TNM stage</u>: I vs II vs III vs Iva vs IVb)	*Model calibration*: NR<br><br>*Other model performance measures*: NR
Cheng 2021, Taiwan<br><br>*External validation 1 (six centres (Canada, US, Netherlands) from TCIA database)*		*Model discrimination (c-index)*: 5yr OS: AUC 0.659 (0.597-0.723); c-index: 0.664 (0.596-0.731); 2yr OS: AUC *(Data not presented in correct row*?)<br><br>*Model calibration*: NR<br><br>*Other model performance measures*: NR
Cheng 2021, Taiwan<br><br>*Development*	**Cheng 2021 models**<br><br>Clinical model (with metabolic tumour volume, MTV)<br><br><u>Age</u>: continuous scale<br><br><u>Sex</u>: male vs female<br><br><u>cT stage</u>: T1 vs T2 vs T3 vs T4a vs T4b)<br><br><u>cN stage</u>: N0 vs N1 vs N2 vs N3<br><br><u>Clinical TNM stage</u>: I vs II vs III vs Iva vs IVb)	*Model discrimination (c-index)*: 5 yr OS: AUC 0.765 (0.717-0.810); c-index: 0.726 (0.682-0.770); 2 yr OS: AUC 0.775 (0.724-0.820)<br><br>*Model calibration*: NR<br><br>*Other model performance measures*: NR
Cheng 2021, Taiwan<br><br>*External validation 1 (six centres (Canada, US, Netherlands) from TCIA database)*		*Model discrimination (c-index)*: 5 yr OS: AUC 0.754 (0.659-0.843); c-index: 0.771 (0.697-0.845); 2 yr OS: AUC (*Data not presented in correct row?*)<br><br>*Model calibration*: NR<br><br>*Other model performance measures*: NR

Study<br><br>(DEV or EV)	Model parameters and cut-offs; algorithm, formula, nomogram or online risk calculator available?	Model performance measures
	HPV: positive vs negative<br><br>MTV: <22.66 cm^3 vs ≥22.66 cm^3	
Cheng 2021, Taiwan<br><br>*Development*	**Cheng 2021 models**<br><br>Clinical model (with MTV, without HPV)<br><br>Age: continuous scale<br><br>Sex: male vs female<br><br>cT stage: T1 vs T2 vs T3 vs T4a vs T4b)<br><br>cN stage: N0 vs N1 vs N2 vs N3<br><br>Clinical TNM stage: I vs II vs III vs Iva vs IVb)<br><br>MTV: <22.66 cm^3 vs ≥22.66 cm^3	*Model discrimination (c-index)*: 5 yr OS: AUC 0.715 (0.663-0.770); c-index: 0.690 (0.645-0.735); 2yr OS AUC 0.738 (0.683-0.793)<br><br>*Model calibration*: NR<br><br>*Other model performance measures*: NR
Cheng 2021, Taiwan<br><br>*External validation 1 (six centres (Canada, US, Netherlands) from TCIA database)*		*Model discrimination (c-index)*: 5 yr OS: AUC 0.657 (0.594-0.721); c-index: 0.664 (0.595-0.733); 2yr OS AUC (*Data not presented in correct row?*)<br><br>*Model calibration*: NR<br><br>*Other model performance measures*: NR
Choi 2020, Republic of Korea<br><br>*Development*	**Choi 2020 nomogram**<br><br>**Model 1**<br><br>T stage: T1-2 vs T3-4	*Model discrimination (c-index)*: Model 1 OS: c-index 0.733 (no CI presented).<br><br>*Model calibration*: NR<br><br>*Other model performance measures*: Integrated Brier score: in both cohorts (DEV and EV), the model performance decreases over time, with a steep decrease up until around 600 days, after which the decrease in model performance flattens off somewhat. After 1500 days, the prediction error rate of the DEV cohort is between 0.2 and 0.25 indicating very poor model performance. The model performance is similar in both cohorts until

**Study**<br><br>**(DEV or EV)**	**Model parameters and cut-offs; algorithm, formula, nomogram or online risk calculator available?**	**Model performance measures**
	<u>N stage</u>: N0-1 vs N2-3<br><br><u>HPV status</u>: positive vs negative<br><br><u>rad-score</u>: high risk vs low risk (median rad-score as cut-off)<br><br>*Nomogram presented for model 1 (2- and 5-yr survival).*	around 600 days, after which the model performs better in the EV cohort compared with the DEV cohort.
Choi 2020, Republic of Korea<br><br>*Internal validation*		*Model discrimination (c-index)*: Model 1 OS: c-index 0.866 (no CI presented).<br><br>*Model calibration*: NR<br><br>*Other model performance measures*: NR
Choi 2020, Republic of Korea<br><br>*External validation*		*Model discrimination (c-index)*: OS: c-index 0.720 (no CI presented).<br><br>*Model calibration*: NR<br><br>*Other model performance measures*: See DEV cohort
Mes 2020, The Netherlands<br><br>*Development*	**Mes 2020 clinical model**<br><br><u>N-stage:</u> no further details<br><br><u>Age at diagnosis:</u> no further details<br><br><u>Sex</u>	*Model discrimination (c-index)*: OS: integrated (i) AUC (95% CI) 0.57 (0.46–0.61); RFS: 0.56 (0.42–0.61)<br><br>*Model calibration*: NR<br><br>*Other model performance measures*: NR
Mes 2020, The Netherlands		*Model discrimination (c-index)*: OS: iAUC (95% CI) 0.74 (0.64–0.83); RFS: 0.71 (0.58–0.82)

Study<br><br>(DEV or EV)	Model parameters and cut-offs; algorithm, formula, nomogram or online risk calculator available?	Model performance measures
External validation		*Model calibration*: NR<br><br>*Other model performance measures*: NR
Mes 2020, The Netherlands<br><br>*Development*	**Mes 2020 clinical + radiomics model**<br><br>N-stage: no further details<br><br>Age at diagnosis: no further details<br><br>Sex<br><br>Radiomic features: 7 latent factors describing tumour intensity (“graylevel-mix” and “meta-firstorder”), shape (“3D geometrics” and “geometrics”) and texture (“meta-graylevelco-occurrence,” “meta-graylevelrunlength,” and “entropy”)	*Model discrimination (c-index)*: OS: iAUC (95% CI) 0.73 (0.62–0.76); RFS: 0.70 (0.56–0.75)<br><br>*Model calibration*: NR<br><br>*Other model performance measures*: NR
Mes 2020, The Netherlands<br><br>*External validation*		*Model discrimination (c-index)*: OS: iAUC (95% CI) 0.81 (0.68–0.91); RFS: 0.78 (0.62–0.83)<br><br>*Model calibration*: NR<br><br>*Other model performance measures*: NR
Rasmussen 2019, Denmark<br><br>*Development*	**P16- model**<br><br>Sex: female (reference) vs male<br><br>Age: HR of event with increasing age	*Model discrimination (c-index)*: T-site recurrence: AUC of 0.752; N-site recurrence: AUC of 0.629; M-site recurrence: AUC of 0.718; Death (NED): AUC of 0.734<br><br>*Model calibration*: NR

**Study**<br><br>**(DEV or EV)**	**Model parameters and cut-offs; algorithm, formula, nomogram or online risk calculator available?**	**Model performance measures**
	<u>PS</u>: 0 (reference) vs 1-3;<br><br><u>Smoking:</u> 0PY (reference) vs 0-10 vs 10-20 vs >20	*Other model performance measures*: No statistical evidence of a difference in model performance (Brier score) between the p16 model and the HPV/p16 model for PFS (NB limited information on Brier scores).
Rasmussen 2019, Denmark<br><br>*External validation*	<u>P16 status</u>: negative (reference) vs positive<br><br><u>T-stage</u>: HR of event with increasing T-stage<br><br><u>N-stage</u>: HR of event with increasing N-stage	*Model discrimination (c-index)*: Not reported.<br><br>*Model calibration*: NR<br><br>*Other model performance measures*: NR
Rasmussen 2019, Denmark<br><br>*Development*	**HPV/P16- model**<br><br><u>Sex</u>: female (reference) vs male<br><br><u>Age</u>: HR of event with increasing age<br><br><u>PS</u>: 0 (reference) vs 1-3	*Model discrimination (c-index)*: T-site recurrence: AUC of 0.756; N-site recurrence: AUC of 0.650; M-site recurrence: AUC of 0.724; Death (NED): AUC of 0.736<br><br>*Model calibration*: NR<br><br>*Other model performance measures*: No statistical evidence of a difference in model performance (Brier score) between the p16 model and the HPV/p16 model for PFS (NB limited information on Brier scores).
Rasmussen 2019, Denmark<br><br>*External validation*	<u>Smoking</u>: 0PY (reference) vs 0-10 vs 10-20 vs >20<br><br><u>HPV_DNA/P16 combination</u>: +/+ reference vs +/- vs -/+ vs -/-	*Model discrimination (c-index)*: NR<br><br>*Model calibration*: NR<br><br>*Other model performance measures*: NR

Study<br><br>(DEV or EV)	Model parameters and cut-offs; algorithm, formula, nomogram or online risk calculator available?	Model performance measures
	T-stage: HR of event with increasing T-stage<br><br>N-stage: HR of event with increasing N-stage<br><br>https://rasmussen.shinyapps.<br><br>io/OPSCCmodelHPV_p16/	
Ward 2014, UK<br><br>*Development*	**Ward 2014 model**<br><br>Tumour-infiltrating lymphocytes (TIL) levels: low vs moderate or high<br><br>T stage: T1/2 vs T3/4<br><br>Smoking status: heavy vs light-, ex-, or non-smoker	*Model discrimination (c-index)*: 3-yr DSS: AUC 0.87<br><br>*Model calibration*: NR<br><br>*Other model performance measures*: Detection rate (sensitivity): 72.7%; false positive rate (1-specificity): 10.2%. Cut-off: -0.945
Ward 2014, UK<br><br>*External validation*		*Model discrimination (c-index)*: 3-yr DSS: AUC 0.82<br><br>*Model calibration*: NR<br><br>*Other model performance measures*: Detection rate (sensitivity): 67%; false positive rate (1-specificity): 11%. Cut-off: -0.945. Likelihood ratio: 11.9
Ma 2023, The Netherlands<br><br>*Internal validation*	**Ma 2023 models**<br><br>***Clinical model***<br><br>HPV status: unknown vs positive vs negative	*Model discrimination (c-index):* 2-yr OS: AUC 0.67 (95% CI 0.55, 0.78); 2-yr LC: AUC 0.67 (95% CI 0.54 0.8); 2-yr RC: AUC 0.6 (95% CI 0.38, 0.83); 2-yr LRC: AUC 0.64 (95% CI 0.5, 0.78); 2-yr DMFS: AUC 0.69 (95% CI 0.56, 0.82); 2-yr DSS: AUC 0.71 (95% CI 0.58, 0.83);2-yr DFS: AUC 0.71 (95% CI 0.62, 0.79)<br><br>*Model calibration: NR*

Study<br><br>(DEV or EV)	Model parameters and cut-offs; algorithm, formula, nomogram or online risk calculator available?	Model performance measures
	T-stage: T4 vs T1-3<br><br>N-stage: N3 vs N2 vs N0-1<br><br>Smoking: non-smoker vs ex-smoker vs current<br><br>Age: continuous<br><br>Sex: female vs male<br><br>WHO Performance status: 1-3 vs 0	*Other model performance measures: NR*
Ma 2023, The Netherlands<br><br>*External validation of Ma 2023*		*Model discrimination (c-index):* 2-yr OS: AUC 0.58 (95% CI 0.51, 0.65); 2-yr LC: AUC 0.68 (95% CI 0.59, 0.75); 2-yr RC: AUC 0.56 (95% CI 0.47, 0.65); 2-yr LRC: AUC 0.6 (95% CI 0.52, 0.67); 2-yr DMFS: AUC 0.56 (95% CI 0.44, 0.67); 2-yr DSS: AUC 0.59 (95% CI 0.51, 0.66); 2-yr DFS: AUC 0.61 (95% CI 0.54, 0.68)<br><br>*Model calibration: NR*<br><br>*Other model performance measures: NR*
Ma 2023, The Netherlands<br><br>*Internal validation*	**Ma 2023 models**<br><br>***Single-label Learning (SLL) based model***<br><br>HPV status: unknown vs positive vs negative<br><br>T-stage: T4 vs T1-3<br><br>N-stage: N3 vs N2 vs N0-1<br><br>Smoking: non-smoker vs ex-smoker vs current<br><br>Age: continuous	*Model discrimination (c-index):* 2-yr OS: AUC 0.69 (95% CI 0.58, 0.8); 2-yr LC: AUC 0.72 (95% CI 0.58, 0.87); 2-yr RC: AUC 0.61 (95% CI 0.41, 0.82) ; 2-yr LRC: AUC 0.71 (95% CI 0.58, 0.84); 2-yr DMFS: AUC 0.69 (95% CI 0.54, 0.83); 2-yr DSS: AUC 0.66 (95% CI 0.53, 0.79; 2-yr DFS: AUC 0.63 (95% CI 0.53, 0.74)<br><br>*Model calibration: NR*<br><br>*Other model performance measures: NR*
Ma 2023, The Netherlands<br><br>*External validation of Ma 2023*		*Model discrimination (c-index):* 2-yr OS: AUC 0.62 (95% CI 0.54, 0.68); 2-yr LC: AUC 0.66 (95% CI 0.58, 0.74); 2-yr RC: AUC 0.56 (95% CI 0.47, 0.64); 2-yr LRC: AUC 0.63 (95% CI 0.56, 0.7); 2-yr DMFS: AUC 0.62 (95% CI 0.5, 0.71); 2-yr DSS: AUC 0.599 (95% CI 0.49, 0.66); 2-yr DFS: AUC 0.59 (95% CI 0.52, 0.65)<br><br>*Model calibration: NR*

Study<br><br>(DEV or EV)	Model parameters and cut-offs; algorithm, formula, nomogram or online risk calculator available?	Model performance measures
	Sex: female vs male<br><br>WHO Performance status: 1-3 vs 0<br><br>CT-based image features	*Other model performance measures: NR*
Ma 2023, The Netherlands<br><br>*Internal validation*	**Ma 2023 models**<br><br>***Multi-label learning (MLL1) based model*** *(ß value in the loss function set to 5)*<br><br>HPV status: unknown vs positive vs negative<br><br>T-stage: T4 vs T1-3<br><br>N-stage: N3 vs N2 vs N0-1<br><br>Smoking: non-smoker vs ex-smoker vs current<br><br>Age: continuous<br><br>Sex: female vs male<br><br>WHO Performance status: 1-3 vs 0<br><br>CT-based image features	*Model discrimination (c-index):* 2-yr OS: AUC 0.71 (95% CI 0.6, 0.81); 2-yr LC: AUC 0.75 (95% CI 0.63, 0.85); 2-yr RC: AUC 0.66 (95% CI 0.4, 0.87); 2-yr LRC: AUC 0.68 (95% CI 0.55, 0.8); 2-yr DMFS: AUC 0.76 (95% CI 0.64, 0.87); 2-yr DSS: AUC 0.65 (95% CI 0.48, 0.8); 2-yr DFS: AUC 0.72 (95% CI 0.63, 0.81)<br><br>*Model calibration: NR*<br><br>*Other model performance measures: NR*
Ma 2023, The Netherlands<br><br>*External validation of Ma 2023*		*Model discrimination (c-index):* 2-yr OS: AUC 0.6 (95% CI 0.53, 0.66); 2-yr LC: AUC 0.64 (95% CI 0.54, 0.72); 2-yr RC: AUC 0.57 (95% CI 0.49, 0.66); 2-yr LRC: AUC 0.59 (95% CI 0.51, 0.66); 2-yr DMFS: AUC 0.66 (95% CI 0.58, 0.76); 2-yr DSS: AUC 0.57 (95% CI 0.48, 0.65); 2-yr DFS: AUC 0.6 (95% CI 0.53, 0.66)<br><br>*Model calibration: NR*<br><br>*Other model performance measures: NR*

<table>
<tr><th>Study

(DEV or EV)</th><th>Model parameters and cut-offs; algorithm, formula, nomogram or online risk calculator available?</th><th>Model performance measures</th></tr>
<tr><td>Ma 2023, The Netherlands

Internal validation</td><td rowspan="2">Ma 2023 models

Multi-label learning (MLL2) based model (ß value in the loss function set to 17)

<u>HPV status:</u> unknown vs positive vs negative

<u>T-stage:</u> T4 vs T1-3

<u>N-stage</u>: N3 vs N2 vs N0-1

<u>Smoking:</u> non-smoker vs ex-smoker vs current

<u>Age</u>: continuous

<u>Sex</u>: female vs male

<u>WHO Performance status:</u> 1-3 vs 0

CT-based image features</td><td>Model discrimination (c-index): 2-yr OS: AUC 0.81 (95% CI 0.73, 0.89); 2-yr LC: AUC 0.79 (95% CI 0.68, 0.88); 2-yr RC: AUC 0.82 (95% CI 0.64, 0.96); 2-yr LRC: AUC 0.76 (95% CI 0.64, 0.85); 2-yr DMFS: AUC 0.81 (95% CI 0.68, 0.92); 2-yr DSS: AUC 0.8 (95% CI 0.7, 0.89); 2-yr DFS: AUC 0.81 (95% CI 0.74, 0.88)

Model calibration: Reasonably good calibration for all endpoints.

Other model performance measures: Sensitivity, specificity, balanced accuracy, F1 score and PR AUC (Precision-Recall Area Under the Curve) presented. Balanced sensitivity and specificity values and balanced accuracies ≥0.70 obtained for all endpoints except DSS. High F-1 score (0.62) and PR-AUC (0.65) observed for DFS only.</td></tr>
<tr><td>Ma 2023, The Netherlands

External validation of Ma 2023</td><td>Model discrimination (c-index): 2-yr OS: AUC 0.72 (95% CI 0.65, 0.79); 2-yr LC: AUC 0.73 (95% CI 0.65, 0.81); 2-yr RC: AUC 0.65 (95% CI 0.56, 0.74); 2-yr LRC: AUC 0.64 (95% CI 0.56, 0.72); 2-yr DMFS: AUC 0.69 (95% CI 0.59, 0.79); 2-yr DSS: AUC 0.78 (95% CI 0.71, 0.85); 2-yr DFS: AUC 0.65 (95% CI 0.58, 0.71)

Model calibration: Reasonably good calibration for distant metastasis, death and any recurrence and death; less good calibration for disease-specific death, local recurrence, loco-regional recurrence and regional recurrence.

Other model performance measures: Sensitivity, specificity, balanced accuracy, F1 score and PR AUC (Precision-Recall Area Under the Curve) presented. Balanced sensitivity and specificity values and balanced accuracies ≥0.70 obtained for DSS, and either high sensitivity or specificity values for other endpoints. No high F1 or PR-AUC scores.</td></tr>
<tr><td>Ma 2023, The Netherlands</td><td>Ma 2023 models

MLL2 + oversampling model</td><td>Model discrimination (c-index): 2-yr OS: AUC 0.8 (95% CI 0.72, 0.87); 2-yr LC: AUC 0.75 (95% CI 0.63, 0.86); 2-yr RC: AUC 0.83 (95% CI 0.67, 0.95); 2-yr LRC: AUC 0.76 (95% CI 0.63, 0.87); 2-yr DMFS: AUC 0.8 (95% CI 0.68, 0.89); 2-yr DSS: AUC 0.82 (95% CI 0.71, 0.91); 2-yr DFS: AUC 0.76 (95% CI 0.68, 0.85)</td></tr>
</table>

**Study**<br><br>**(DEV or EV)**	**Model parameters and cut-offs; algorithm, formula, nomogram or online risk calculator available?**	**Model performance measures**
Internal validation	<u>HPV status:</u> unknown vs positive vs negative<br><br><u>T-stage:</u> T4 vs T1-3<br><br><u>N-stage</u>: N3 vs N2 vs N0-1<br><br><u>Smoking:</u> non-smoker vs ex-smoker vs current<br><br><u>Age</u>: continuous<br><br><u>Sex</u>: female vs male<br><br><u>WHO Performance status:</u> 1-3 vs 0<br><br>CT-based image features	*Model calibration: NR*<br><br>*Other model performance measures: NR*
Ma 2023, The Netherlands<br><br>*External validation of Ma 2023*		*Model discrimination (c-index):* 2-yr OS: AUC 0.73 (95% CI 0.65, 0.8); 2-yr LC: AUC 0.63 (95% CI 0.54, 0.71); 2-yr RC: AUC 0.7 (95% CI 0.61, 0.79); 2-yr LRC: AUC 0.68 (95% CI 0.61, 0.75); 2-yr DMFS: AUC 0.55 (95% CI 0.45, 0.66); 2-yr DSS: AUC 0.78 (95% CI 0.71, 0.85); 2-yr DFS: AUC 0.72 (95% CI 0.66, 0.78)<br><br>*Model calibration: NR*<br><br>*Other model performance measures: NR*
Ma 2023, The Netherlands<br><br>*Internal validation*	**Ma 2023 models**<br><br>***MLL2 + radiomics model***<br><br><u>HPV status:</u> unknown vs positive vs negative<br><br><u>T-stage:</u> T4 vs T1-3<br><br><u>N-stage</u>: N3 vs N2 vs N0-1	*Model discrimination (c-index):* 2-yr OS: AUC 0.82 (95%CI 0.75, 0.89); 2-yr LC: AUC 0.79 (95% CI 0.64, 0.9); 2-yr RC: AUC 0.72 (95% CI 0.48, 0.93); 2-yr LRC: AUC 0.73 (95% 0.61, 0.85); 2-yr DMFS: AUC 0.77 (95% CI 0.64, 0.88); 2-yr DSS: AUC 0.85 (95% CI 0.75, 0.92); 2-yr DFS: AUC 0.81 (95% CI 0.73, 0.88)<br><br>*Model calibration: NR*<br><br>*Other model performance measures: NR*
Ma 2023, The Netherlands		*Model discrimination (c-index):* 2-yr OS: AUC 0.7 (95% CI 0.63, 0.78); 2-yr LC: AUC 0.65 (95% CI 0.58, 0.74); 2-yr RC: AUC 0.59 (95% CI 0.5, 0.69); 2-yr LRC: AUC 0.66 (95% CI

**Study**<br><br>**(DEV or EV)**	**Model parameters and cut-offs; algorithm, formula, nomogram or online risk calculator available?**	**Model performance measures**
External validation of Ma 2023	Smoking: non-smoker vs ex-smoker vs current<br><br>Age: continuous<br><br>Sex: female vs male<br><br>WHO Performance status: 1-3 vs 0<br><br>Radiomics score based on CT image features	0.59, 0.73); 2-yr DMFS: AUC 0.69 (95% CI 0.57, 0.8); 2-yr DSS: AUC 0.76 (95% CI 0.68, 0.84); 2-yr DFS: AUC 0.66 (95% CI 0.6, 0.73)<br><br>*Model calibration: NR*<br><br>*Other model performance measures: NR*

Notes: Where OS only stated, years were not specified
