# Supplementary material for: Systematic review of prognostic models for predicting recurrence and survival in patients with treated oropharyngeal cancer
Source: BMJ Open. 2024 Dec 5;14(12):e090393. doi: 10.1136/bmjopen-2024-090393 (PMC11624838; doi:10.1136/bmjopen-2024-090393)
Supplement: online supplemental file 6 [file bmjopen-14-12-s006.pdf]

Supplemental material 6

Excluded studies

Study	*Reason for exclusion*
Alidoost, M.; Nickens, D.; Alhadab, A. Machine learning (ML) methods for progression-free survival (PFS) and overall survival (OS) prediction: Re-analysis of javelin head and neck 100 data. Clinical Pharmacology and Therapeutics - Volume 111, Issue 0, pp. S76-S77 - published 2022-01-01	Conference abstract. No sub-group analysis.
Alfieri S, Romanò R, Bologna M, Calareso G, Corino V, Mirabile A, Ferri A, Bellanti L, Poli T, Marcantoni A, Grosso E, Tarsitano A, Battaglia S, Blengio F, De Martino I, Valerini S, Vecchio S, Richetti A, Deantonio L, Martucci F, Grammatica A, Ravanelli M, Ibrahim T, Caruso D, Locati LD, Orlandi E, Bossi P, Mainardi L, Licitra LF. Prognostic role of pre-treatment magnetic resonance imaging (MRI)-based radiomic analysis in effectively cured head and neck squamous cell carcinoma (HNSCC) patients. Acta Oncol. 2021 Sep;60(9):1192-1200. doi: 10.1080/0284186X.2021.1924401. Epub 2021 May 26. PMID: 34038324.	No sub-group analysis.
Altazi BA, Zhang GG, Naghavi AO, Moros EG, Caudell JJ. Radiomic Features Prognostic for Recurrence in Human Papilloma virus Positive Oropharyngeal Cancer. Int J Radiation Oncology Biology Physics 2016;96(2):S70-S71	No external validation.
Bos P, van den Brekel MWM, Gouw ZAR, Al-Mamgani A, Taghavi M, Waktola S, Aerts HJWL, Castelijns JA, Beets-Tan RGH, Jasperse B. Improved outcome prediction of oropharyngeal cancer by combining clinical and MRI features in machine learning models. Eur J Radiol. 2021 Jun;139:109701. doi: 10.1016/j.ejrad.2021.109701. Epub 2021 Apr 8. PMID: 33865064.	No external validation
Boscolo-Rizzo P, D'Alessandro A, Polesel J, Borsetto D, Tofanelli M, Deganello A, Tomasoni M, Nicolai P, Bossi P, Spinato G, Menegaldo A, Ciorba A, Pelucchi S, Bianchini C, Cazzador D, Ramaciotti G, Lupato V, Giacomarra V, Molteni G, Marchioni D, Fabbris C, Occhini A, Bertino G, Fussey J, Tirelli G. Different inflammatory blood markers correlate with specific outcomes in incident HPV-negative head and neck squamous cell carcinoma: a retrospective cohort study. BMC Cancer. 2022 Mar 5;22(1):243. doi: 10.1186/s12885-022-09327-4. PMID: 35248020; PMCID: PMC8897882.	Individual prognostic factors.
Brody RM, Shimunov D, Cohen RB, Lin A, Lukens JN, Hartner L, Aggarwal C, Duvvuri U, Montone KT, Jalaly JB, LiVolsi VA, Carey RM, Shanti RM, Rajasekaran K, Chalian AA, Rassekh CH, Cannady SB, Newman JG, O'Malley BW, Weinstein GS, Gimotty PA, Basu D. A benchmark for oncologic outcomes and model for lethal recurrence risk after transoral robotic resection of HPV-related oropharyngeal cancers.	No external validation.

Oral Oncol. 2022 Apr;127:105798. doi: 10.1016/j.oraloncology.2022.105798. Epub 2022 Mar 1. PMID: 35245888; PMCID: PMC9288202.	
Castelli J, Depeursinge A, Ndoh V, Prior JO, Ozsahin M, Devillers A et al. 4PO-0604: A PET-based nomogram to predict survival in oropharyngeal cancers radiotherapy. Radiotherapy and Oncology 2017;123:S315	Conference abstract of included study
Chen L, Zeng H, Zhang M, Luo Y, Ma X. Histopathological image and gene expression pattern analysis for predicting molecular features and prognosis of head and neck squamous cell carcinoma. Cancer Med. 2021 Jul;10(13):4615-4628. doi: 10.1002/cam4.3965. Epub 2021 May 13. PMID: 33987946; PMCID: PMC8267162.	Tumour site as model parameter.
De Felice F, Lei M. PO-0971 Locally advanced oropharyngeal cancer: a dynamic nomogram. Radiotherapy and Oncology 2021;161:S804-S805<br><br>DOI:10.1016/S0167-8140(21)07422-3	No external validation. Conference abstract.
Fakhry C, Tewari SR, Zhang L, Windon MJ, Bigelow EO, Drake VE, Rooper LM, Troy T, Ha P, Miles BA, Mydlarz WK, Eisele DW, D'Souza G. RTOG-0129 risk groups are reproducible in a prospective multicenter heterogeneously treated cohort. Cancer. 2021 Oct 1;127(19):3523-3530. doi: 10.1002/cncr.33682. Epub 2021 Jun 18. PMID: 34143891.	No assessment of model performance
Folkert MR, Oh JH, Setton J, Apte AP, Thorstad WL, Schoder H et al. Predictive Modeling of Outcomes Following Definitive Chemoradiation Therapy for Oropharyngeal Cancer Based on FDGPET Image Characteristics<br><br>International Journal of Radiation Oncology, Biology, Physics 2013; 87(2):S3	Conference abstract of included study
Fujima, N., Andreu-Arasa, V.C., Meibom, S.K. et al. Prediction of the local treatment outcome in patients with oropharyngeal squamous cell carcinoma using deep learning analysis of pretreatment FDG-PET images. BMC Cancer 21, 900 (2021). https://doi.org/10.1186/s12885-021-08599-6	No external validation. Radiomics parameters only.
Granata R, Perrone F, Miceli R, Franceschini M, Orli E, Bossi P et al. Tumor stage humanpapilloma virus (HPV) and smoking status affect the survival of patients with oropharyngeal cancer: An Italian validation study. Annals of Oncology 2010; Volume 21, Issue 0, pp. viii317	Conference abstract of included study (Granata 2012).
He S, Yi Z, Ruan S, Anastasio M, Mutic S, Thorstad W, Gay H, Wang X, Li H. MicroRNA-Based Survival and Relapse Prognosis for Oropharyngeal Cancer Treatment by Use of Cox Regression and Belief Function Theory. Medical Physics 2019; Volume 46, Issue 0, pp. e458.	Unclear if external validation. Conference abstract only, no numerical results.
Hoebers F, Rios Velazquez E, Aerts HJWL, Rietbergen MM, Brakenhoff RH, Speel EJ et al. OC-0373: An HPV-based predictive model for	Conference abstract of included study

oropharynx cancer yields more accurate predictions than TNM staging system. ESTRO 2014; 133: Suppl 144.	(Rios-Velazquez 2014).
Hutz M, McCalister BL, Martin JR, Malliaris ME, Bier-Laning C. Validation of a Survival Prediction Model for Oropharyngeal Squamous Cell Carcinoma<br><br>Otolaryngology–Head and Neck Surgery 2017; 157(1S)	Conference abstract only. External validation of a previous model (not referenced, unclear which model).
Kharouta MZ, Patil N, Torres-Saavedra P, Stokes WA, Henson C, Bhatt A.<br><br>ABSTRACT ONLY|, SEPTEMBER 01, 2021<br><br>863MO Nomogram for predicting survival for patients receiving definitive chemoradiation in locally advanced squamous cell carcinoma of the head and neck: A secondary analysis of NRG/RTOG 0129, 0522, and 1016. Annals of Oncology 2021; VOL 32, SUPPLEMENT 5, S788.	Patients with HNSCC, no sub-group analysis for oropharyngeal cancer. Conference abstract only.
Laliotis GI, Issa M, Klamer B, Karivedu V, Bhateja P, Dibs K et al. Expansion cohort validation of a clinical predictive model for head and neck cancer survival in patients treated with immune checkpoint inhibitors. Journal of Clinical Oncology 2021; Vol 39 (15), suppl 6030.	No external validation. Conference abstract only.
Le WT, Vorontsov E, Romero FP, Seddik L, Elsharief MM, Nguyen-Tan PF, Roberge D, Bahig H, Kadoury S. Cross-institutional outcome prediction for head and neck cancer patients using self-attention neural networks. Sci Rep. 2022 Feb 24;12(1):3183. doi: 10.1038/s41598-022-07034-5. PMID: 35210482; PMCID: PMC8873259.	No sub-group analysis. Unclear of appropriate external validation.
W. Lv, H. Feng, D. Du, J. Ma and L. Lu, "Complementary Value of Intra- and Peri-Tumoral PET/CT Radiomics for Outcome Prediction in Head and Neck Cancer," in IEEE Access, vol. 9, pp. 81818-81827, 2021, doi: 10.1109/ACCESS.2021.3085601.	No sub-group analysis.
Lv W, Ashrafinia S, Ma J, Lu L, Rahmim A. Multi-Level Multi-Modality Fusion Radiomics: Application to PET and CT Imaging for Prognostication of Head and Neck Cancer. IEEE J Biomed Health Inform. 2020 Aug;24(8):2268-2277. doi: 10.1109/JBHI.2019.2956354. Epub 2019 Dec 4. PMID: 31804945.	No sub-group analysis.
Joo L, Bae YJ, Choi YJ, Lee YS, Chung SR, Hyun Suh CH et al. Prediction model for cervical lymph node metastasis in human papillomavirus-related oropharyngeal squamous cell carcinomas. European Radiology (2021) 31:7429–7439	Model to be used to determine lymph node metastasis pre-treatment.
Miccichè F, Chiloiro G, Longo S. PO-0842: PRO.M.E.THE.O.: the development of an italian multicentric OS predictive model in oropharynx cancer. Radiotherapy and Oncology 2020: 52:S454. DOI:10.1016/S0167-8140(21)00859-8	No external validation. Conference abstract only.

Min Park Y, Yol Lim J, Woo Koh Y, Kim SH, Chang Choi E. Prediction of treatment outcome using MRI radiomics and machine learning in oropharyngeal cancer patients after surgical treatment. Oral Oncol. 2021 Nov;122:105559. doi: 10.1016/j.oraloncology.2021.105559. Epub 2021 Oct 11. PMID: 34649039.	No external validation
Patil S, Linge A, Grosser M, Lohaus F, Gudziol V, Kemper M, Nowak A, Haim D, Tinhofer I, Budach V, Guberina M, Stuschke M, Balermpas P, Rödel C, Schäfer H, Grosu AL, Abdollahi A, Debus J, Ganswindt U, Belka C, Pigorsch S, Combs SE, Boeke S, Zips D, Baretton GB, Baumann M, Krause M, Löck S; DKTK-ROG. Development and validation of a 6-gene signature for the prognosis of loco-regional control in patients with HPV-negative locally advanced HNSCC treated by postoperative radio(chemo)therapy. Radiother Oncol. 2022 Jun;171:91-100. doi: 10.1016/j.radonc.2022.04.006. Epub 2022 Apr 13. PMID: 35429503.	No sub-group analysis for OPSCC
Price JM, Mistry HB, Betts G, Cheadle EJ, Dixon L, Garcez K, Illidge T, Iyizoba-Ebozue Z, Lee LW, McPartlin A, Prestwich RJD, Papageorgiou S, Pritchard DJ, Sykes A, West CM, Thomson DJ. Pretreatment Lymphocyte Count Predicts Benefit From Concurrent Chemotherapy With Radiotherapy in Oropharyngeal Cancer. J Clin Oncol. 2022 Jul 10;40(20):2203-2212. doi: 10.1200/JCO.21.01991. Epub 2022 Apr 6. PMID: 35385334; PMCID: PMC9273368.	Single prognostic factor
Qiang W, Dai Y, Xing X, Sun X. Identification and validation of a prognostic signature and combination drug therapy for immunotherapy of head and neck squamous cell carcinoma. Computational and Structural Biotechnology Journal 2021; Volume 19: Pages 1263-1276. ISSN 2001-0370, https://doi.org/10.1016/j.csbj.2021.01.046.	No sub-group analysis for OPSCC
Rabasco Meneghetti A, Zwanenburg A, Leger S, Leger K, Troost EGC, Linge A et al. Definition and validation of a radiomics signature for loco-regional tumour control in patients with locally advanced head and neck squamous cell carcinoma. Clinical and Translational Radiation Oncology 2021; Volume 26:Pages 62-70. https://doi.org/10.1016/j.ctro.2020.11.011.	No sub-group analysis for OPSCC
Shen Y, Li L, Lu Y, Zhang M, Huang X and Tang X. Establishment and Validation of a Comprehensive Prognostic Model for Patients With HNSCC Metastasis. Front. Genet. 2021; 12:685104. doi: 10.3389/fgene.2021.685104	No sub-group analysis for OPSCC
Song B, Yang K, Garneau J, Lu C, Li L, Lee J, Stock S, Braman NM, Koyuncu CF, Toro P, Fu P, Koyfman SA, Lewis JS Jr, Madabhushi A. Radiomic Features Associated With HPV Status on Pretreatment Computed Tomography in Oropharyngeal Squamous Cell Carcinoma Inform Clinical Prognosis. Front Oncol. 2021 Sep 7;11:744250. doi: 10.3389/fonc.2021.744250. PMID: 34557418; PMCID: PMC8454409.	External validation of models not in independent cohort (combined an external cohort with part of the cohort used for development of model).

Wenyan W, Zhen W, Zengtong Z, Jiang L. A prognostic scoring model based on the HPV status of oropharyngeal carcinoma patients treated with postoperative radiotherapy in China. Int J Clin Exp Pathol 2019;12(5):1868-1876	No external validation
Wu Q, Wang M, Liu Y, Wang X, Li Y, Hu X, Qiu Y, Liang W, Wei Y, Zhong Y. HPV Positive Status Is a Favorable Prognostic Factor in Non-Nasopharyngeal Head and Neck Squamous Cell Carcinoma Patients: A Retrospective Study From the Surveillance, Epidemiology, and End Results Database. Front Oncol. 2021 Sep 24;11:688615. doi: 10.3389/fonc.2021.688615. PMID: 34631523; PMCID: PMC8497986.	No external validation
Zhang Y, Chen P, Zhou Q, Wang H, Hua Q, Wang J, Zhong H. A Novel Immune-Related Prognostic Signature in Head and Neck Squamous Cell Carcinoma. Front Genet. 2021 Jun 18;12:570336. doi: 10.3389/fgene.2021.570336. PMID: 34220923; PMCID: PMC8249947.	No sub-group analysis.

Full text studies excluded as no standard clinical parameters included in model or conference abstract only

**Study**	**Model parameters**
Aerts HJ, Velazquez ER, Leijenaar RT, Parmar C, Grossmann P, Carvalho S, Bussink J, Monshouwer R, Haibe-Kains B, Rietveld D, Hoebers F, Rietbergen MM, Leemans CR, Dekker A, Quackenbush J, Gillies RJ, Lambin P. Decoding tumour phenotype by noninvasive imaging using a quantitative radiomics approach. Nat Commun. 2014 Jun 3;5:4006. doi: 10.1038/ncomms5006. Erratum in: Nat Commun. 2014;5:4644. Cavalho, Sara [corrected to Carvalho, Sara]. PMID: 24892406; PMCID: PMC4059926.	Radiomics signature developed in non-small lung cancer patients (then externally validated in oropharyngeal patients -Leijenaar 2015)
Castelli J, Depeursinge A, Ndoh V, Prior JO, Ozsahin M, Devillers A, Bouchaab H, Chajon E, de Crevoisier R, Scher N, Jegoux F, Laguerre B, De Bari B, Bourhis J. A PET-based nomogram for oropharyngeal cancers. Eur J Cancer. 2017 Apr;75:222-230. doi: 10.1016/j.ejca.2017.01.018. Epub 2017 Feb 24. PMID: 28237868.	PET-FDG parameters
Cavalieri S, Serafini MS, Carenzo A, Canevari S, Brakenhoff RH, Leemans R et al. Clinical Validity of a Prognostic Gene Expression Cluster-Based Model in Human Papillomavirus–Positive Oropharyngeal Carcinoma. JCO Precision Oncology no. 5 (2021) 1666-1676. DOI: 10.1200/PO.21.00094	Genetic signature. Conference abstract only.
Corredor G, Lu C, Koyuncu C, Bera K, Toro P, Fu P, Koyfman SA, Chute D, Adelstein DJ, Thorstad W, Bishop JA, Faraji F, Lewis J Jr., Madabhushi A. Computerized features of spatial interplay of tumor-infiltrating lymphocytes predict disease recurrence in p16+ oropharyngeal squamous cell carcinoma: A multisite validation study. Journal of Clinical Oncology 2020; 38 (15 suppl): A6559<br>AND<br><br>Corredor G, Lewis J, Lu C, Toro P, Fu P, Thorstad W, Bishop J, Faraji F, Madabhushi A. The spatial patterns of tumor-infiltrating lymphocytes (tils) are more prognostic than til density in p16+ oropharyngeal squamous cell carcinoma patients. Journal: Modern Pathology; 33:1187-1188<br><br>AND<br>Koyuncu C, Corredor G, Lu C, Toro P, Bera K, Fu P, Koyfman SA, Chute D, Adelstein DJ, Thorstad W, Bishop JA, Faraji F, Lewis JS, Madabhushi A. Combination of tumor multinucleation and spatial arrangement of tumor-infiltrating lymphocytes to predict overall survival in oropharyngeal squamous cell carcinoma: A multisite study DOI: 10.1200/JCO.2020.38.15_suppl.6566 Journal of Clinical Oncology 2020	Model based on *"computerized features of spatial interplay of tumour-infiltrating lymphocytes"*
Deschuymer S, Sørensen BS, Dok R, Laenen A, Hauben E, Overgaard J, Nuyts S. Prognostic value of a 15-gene hypoxia classifier in oropharyngeal cancer treated with accelerated	15-gene hypoxia classifier

chemoradiotherapy. Strahlenther Onkol. 2020 Jun;196(6):552-560. doi: 10.1007/s00066-020-01595-y. Epub 2020 Feb 20. PMID: 32080773	
Egelmeer A, Jorog J, Oberlje C, Kremer B, Horner J, Slevin N, West C, Lambin GP. Development and external validation of a nomogram predicting survival an d local control in oropharyngeal carcinoma patients. Radiotherapy and Oncology 2010; Volume 96, Issue 0, pp. S313 (poster 907)	Conference abstract only.
Folkert MR, Setton J, Apte AP, Grkovski M, Young RJ, Schöder H, Thorstad WL, Lee NY, Deasy JO, Oh JH. Predictive modeling of outcomes following definitive chemoradiotherapy for oropharyngeal cancer based on FDG-PET image characteristics. Phys Med Biol. 2017 Jul 7;62(13):5327-5343. doi: 10.1088/1361-6560/aa73cc. Epub 2017 Jun 12. PMID: 28604368; PMCID: PMC5729737.	FDG-PET intensity and shape features
Gao G, Gay HA, Chernock RD, Zhang TR, Luo J, Thorstad WL, Lewis JS Jr, Wang X. A microRNA expression signature for the prognosis of oropharyngeal squamous cell carcinoma. Cancer. 2013 Jan 1;119(1):72-80. doi: 10.1002/cncr.27696. Epub 2012 Jun 26. PMID: 22736309; PMCID: PMC3461127.	6 miRNA signature<br><br>Development study
Giraud P, Giraud P, Nicolas E, Boisselier P, Alfonsi M, Rives M, Bardet E, Calugaru V, Noel G, Chajon E, Pommier P, Morelle M, Perrier L, Liem X, Burgun A, Bibault JE. Interpretable Machine Learning Model for Locoregional Relapse Prediction in Oropharyngeal Cancers. Cancers (Basel). 2020 Dec 28;13(1):57. doi: 10.3390/cancers13010057. PMID: 33379188; PMCID: PMC7795920.	Radiomics features (vVxel Volume, grey level size zone matrix Small Area Emphasis (glszmSAE), gldm Dependence Non Uniformity Normalized (gldmDNUN), Sex and Age
Gleber-Netto FO, Rao X, Erikson K, Akagi K, Johnson FM, Wang J, Califano J, Gillison ML, Myers JN, Pickering CR. Abstract 4621: Risk stratification and biomarker discovery in HPV-positive oropharynx squamous cell carcinoma determined by HPV and human gene expression profile associations Proceedings: AACR Annual Meeting 2018; April 14-18, 2018; Chicago, IL	41 gene expression signature
Hess AK, Jöhrens K, Zakarneh A, Balermpas P, Von Der Grün J, Rödel C, Weichert W, Hummel M, Keilholz U, Budach V, Tinhofer I. Characterization of the tumor immune micromilieu and its interference with outcome after concurrent chemoradiation in patients with oropharyngeal carcinomas. Oncoimmunology. 2019 May 25;8(8):1614858. doi: 10.1080/2162402X.2019.1614858. PMID: 31413922; PMCID: PMC6682352.	Genetic signature (ISRS: immune signature risk score)

Kanwar A, Mohamed ASR, Court LE, Zhang L, Marai GE, Canahuate G et al.<br><br>Fuller Development of a Predictive Quantitative Contrast Computed Tomography-Based Feature (Radiomics) Profile for Local Recurrence in Oropharyngeal Cancers Int J Radiation Oncology Biology Physics volume 96, issue 2, Supplement s191, October 01, 2016.	4-feature radiomic signature from pretherapy imaging
Leijenaar RT, Carvalho S, Hoebers FJ, Aerts HJ, van Elmpt WJ, Huang SH, Chan B, Waldron JN, O'sullivan B, Lambin P. External validation of a prognostic CT-based radiomic signature in oropharyngeal squamous cell carcinoma. Acta Oncol. 2015;54(9):1423-9. doi: 10.3109/0284186X.2015.1061214. Epub 2015 Aug 12. PMID: 26264429.	4-feature radiomics signature1) “First order statistics: Energy”,2) “Shape: Compactness”, 3) “Gray level run length: Gray level non-uniformity”, 4) Wavelet (HLH) “Gray level run length: Gray level non-uniformity”
Liu X, Liu P, Chernock RD, Kuhs KAL, Lewis JS Jr, Luo J, Gay HA, Thorstad WL, Wang X. A prognostic gene expression signature for oropharyngeal squamous cell carcinoma. EBioMedicine. 2020 Nov;61:102805. doi: 10.1016/j.ebiom.2020.102805. Epub 2020 Oct 7. PMID: 33038770; PMCID: PMC7648117.	60-gene signature
Liu X, Liu P, Chernock RD, Yang Z, Lang Kuhs KA, Lewis JS, Luo J, Li H, Gay HA, Thorstad WL, Wang X. A MicroRNA Expression Signature as Prognostic Marker for Oropharyngeal Squamous Cell Carcinoma. J Natl Cancer Inst. 2021 Jun 1;113(6):752-759. doi: 10.1093/jnci/djaa161. PMID: 33057626; PMCID: PMC8168274.	26-miRNA signature
Lu C, Luo J, Bishop J, Thorstad W, Madabhushi A, Lewis JS. A Quantitative Histomorphometric Classifier (QuHbIC) for Risk Stratification in p16-Positive<br><br>Oropharyngeal Squamous Cell Carcinoma USCAP 2018 107TH ANNUAL MEETING. A1345.	Histomorphometric-based image classifier (QuHbIC): computer-extracted nuclear<br><br>morphologic features from digitally-scanned tumour H&E slides.
Lv W, Xu H, Han X, Zhang H, Ma J, Rahmim A, Lu L. Context-Aware Saliency Guided Radiomics: Application to Prediction of Outcome and HPV-Status from Multi-Center PET/CT Images of Head and Neck Cancer. Cancers (Basel). 2022 Mar 25;14(7):1674. doi: 10.3390/cancers14071674. PMID: 35406449; PMCID: PMC8996849.	Prognostic value of context-aware saliency-guided radiomics in 18F-FDG PET/CT images

Mes SW, van Velden FHP, Peltenburg B, Peeters CFW, Te Beest DE, van de Wiel MA, Mekke J, Mulder DC, Martens RM, Castelijns JA, Pameijer FA, de Bree R, Boellaard R, Leemans CR, Brakenhoff RH, de Graaf P. Outcome prediction of head and neck squamous cell carcinoma by MRI radiomic signatures. Eur Radiol. 2020 Nov;30(11):6311-6321. doi: 10.1007/s00330-020-06962-y. Epub 2020 Jun 4. PMID: 32500196; PMCID: PMC7554007.	Factors representing 3D geometrics, meta-gray level co-occurrence, meta-first order, gray level mix, meta-gray level run length, geometrics, and entropy<br><br>***NB other models from this study are included***
Oh J, Folkert M, Setton J, Apte A, Grkovski M, Young R, Schoder H, Thorstad W, Lee N, Deasy J. TH-AB-201-03: PET-Based Radiomics to Predict Outcomes Following Definitive Chemoradiotherapy for Oropharyngeal Cancer Medical Physics, 44 (6), June 2017.	Conference abstract only. Model based on PET-based radiomics.
Shen S, Bai J, Wei Y, Wang G, Li Q, Zhang R, Duan W, Yang S, Du M, Zhao Y, Christiani DC, Chen F. A seven-gene prognostic signature for rapid determination of head and neck squamous cell carcinoma survival. Oncol Rep. 2017 Dec;38(6):3403-3411. doi: 10.3892/or.2017.6057. Epub 2017 Oct 24. PMID: 29130107; PMCID: PMC5783586.	7 gene signature
Wong N, Khwaja SS, Baker CM, Gay HA, Thorstad WL, Daly MD, Lewis JS Jr, Wang X. Prognostic microRNA signatures derived from The Cancer Genome Atlas for head and neck squamous cell carcinomas. Cancer Med. 2016 Jul;5(7):1619-28. doi: 10.1002/cam4.718. Epub 2016 Apr 25. PMID: 27109697; PMCID: PMC4944889.	4 miRNA signature
Wong N, Khwaja SS, Baker CM, Gay HA, Thorstad WL, Daly MD, Lewis JS Jr, Wang X. Prognostic microRNA signatures derived from The Cancer Genome Atlas for head and neck squamous cell carcinomas. Cancer Med. 2016 Jul;5(7):1619-28. doi: 10.1002/cam4.718. Epub 2016 Apr 25. PMID: 27109697; PMCID: PMC4944889.	4-miRNA signature
