# Supplementary material for: Systematic review of prognostic models for predicting recurrence and survival in patients with treated oropharyngeal cancer
Source: BMJ Open. 2024 Dec 5;14(12):e090393. doi: 10.1136/bmjopen-2024-090393 (PMC11624838; doi:10.1136/bmjopen-2024-090393)
Supplement: online supplemental file 7 [file bmjopen-14-12-s007.pdf]

Supplementary material 7

Risk stratification models

Main study characteristics

Study<br>(D or EV)	Model and cancer location	Source of data (n, and n included in analysis)	Age (mean y (SD)), sex	Cancer stage	TNM	HPV status and measurement method	Smoking, alcohol, co-morbidities	Treatment	Inclusion criteria/ comments on cohort representa tiveness.	Outcomes, length of follow-up
**RTOG-0129 RPA risk stratification model**										
Ang 2010, US<br>*Developme nt*<br><br>*NB patient characteristi cs reported for n=323 with known HPV status*	RTOG-0129 RPA model<br><br>Carcinoma of the oral cavity, oropharynx, hypophary nx, or larynx	Patients recruited July 2002 - May 2005 as part of randomised controlled trial; retrospective analysis; n=433; **n=266** (61%) in analysis	HPV+: median 53.5 (range 31-78); HPV-: 57 (387-82).<br>84% male.	TNM vr NR<br><br>III: 14%<br>IV: 86%	TNM vr NR<br><br>T2: 31%<br>T3: 39%<br>T4: 30%<br>N0: 7.4%<br>N1: 14.2%,<br>N2a:11.5%<br>N2b: 33.1%<br>N2c: 24.1%<br>N3: 9.6%	HPV+: 64%<br>HPV-: 36%<br><br>Tumour p16 protein expression (immunohistoc hemical analysis); Tumour HPV DNA detection (in situ hybridization)	HPV+: Median n of pack years 12.2 (range 0-152); HPV-: 36.5 (0-96). Never smoker (23%), former (51%), current (17%) and unknown (9%).<br>No details on alcohol.<br>Zubrod score 0-1: 71.9%, 2-3: 24.2%, unknown 3.8%.	Fractionati on radiothera py and cisplatin: 51%<br><br>Accelerate d-fractionatio n radiothera py and cisplatin: 49%	US trial population (RTOG-129 study). Eligibility criteria: presence of untreated, pathological ly confirmed, stage III or IV squamous-cell carcinoma of the oral cavity, oropharynx, hypopharyn x, or larynx without distant	OS: defined as time from randomisation to death.<br><br>PFS: defined as time from date of randomisation to death or first documented relapse categorised as local–regional recurrence or distant metastases.<br>Median follow up 4.8 y (range, 0.3 to 6.5y)

Study (D or EV)	Model and cancer location	Source of data (n, and n included in analysis)	Age (mean y (SD)), sex	Cancer stage	TNM	HPV status and measurement method	Smoking, alcohol, co-morbidities	Treatment	Inclusion criteria/ comments on cohort representativeness.	Outcomes, length of follow-up
									metastases (M0); Zubrod's performance status score of 0 or 1 (asymptomatic or symptomatic but ambulatory, respectively); age of 18 years or older; and adequate bone marrow, hepatic, and renal function.	
Granata 2012, Italy<br><br>*External validation*	RTOG-0129 RPA model<br><br>OPSCC (no further details)	Consecutive patients treated at one Italian institution 2003 - 2009; retrospective analysis; n=140; n= **120** (86%) in analysis	Median (range) 61.0 (37-88)<br>80% male	TNM vr NR<br><br>I: 0.8%<br>II: 5.8%<br>III: 8.3%<br>IVa:75.8% IVb: 9.2%	TNM vr NR<br><br>T1: 14.2%<br>T2: 30.8%<br>T3: 12.5%<br>T4: 41.7%	p16+: 54.6%<br>p16-:45.4%<br><br>Tumour p16 protein expression (immunohistochemical analysis)	Median (range) packs/year: 30 (0-120). No smoking: 20.8%, smoking: 79.2%<br>No details on alcohol.	Induction CT +CRT: 32.5%<br>CRT: 53.3%<br>RT only: 14.2%	Single Italian institution (consecutive patients). Not explicitly stated whether patients treated with	OS: defined as time from the date of starting therapy to the date of death due to any cause, censoring at the date of last available follow-up assessment for living patients.<br>Median follow-up of 23 months (interquartile range: 13–39 months)

Study<br>(D or EV)	Model and cancer location	Source of data (n, and n included in analysis)	Age (mean y (SD)), sex	Cancer stage	TNM	HPV status and measurement method	Smoking, alcohol, co-morbidities	Treatment	Inclusion criteria/ comments on cohort representa tiveness.	Outcomes, length of follow-up
					Tis: 0.8%<br>N0: 12.5%<br>N1: 10%<br>N2a: 2.5%<br>N2b: 40.8%<br>N2c: 25%<br>N3: 9.2%		ECOG status- 0: 73.3%, 1: 23.3%, 2: 3.4%		curative intent.	
Rietbergen 2013, The Netherlands<br><br>*External validation*	RTOG-0129 RPA model<br><br>OPSCC HPV+: tonsil 58.9%, base of tongue 31.3%, soft palate 5.5%, oropharynx nos 4.3%; HPV-: tonsil 38.4%, base of	All patients with an OPSCC diagnosed at two Dutch University hospitals Jan 2000 - Dec 2006; retrospective analysis; n=906; **n=721** in analysis (those treated with curative intent and with model	HPV+: mean age at diagnosis 60.53 (median 58.14), HPV-: mean 60.94 (median 59.67).<br>67% male<br><br>*NB based on n=809*	TNM vr NR<br><br>I-VI	TNM vr NR<br><br>HPV+: T1-2: 54%<br>T3-4: 46%<br>Tx: 0<br>N0: 14.7%<br>N1-3: 85.3%<br>Nx: 0<br><br>HPV-: T1-2: 39.1%<br>T3-4:	HPV+: 19.4%<br>HPV-: 76.8%<br><br>Tumour p16 protein expression (immunohistochemical analysis); Tumour HPV DNA detection (PCR)<br><br>*NB based on n=809*	HPV+: 0-10 pack years 40.5%, 11-24 PY 16.0%, >24 PY 42.9%, unknown 0.6%. HPV-: 0-10 PY 6.3%, 11-24 PY 9.3%, >24 PY 83.1%, unknown 1.2%.<br>HPV+: 0-100 unit yrs 68.7%, 111-149 UY 9.2%, >149 UY 21.5%, unknown 0.6%. HPV-: 0-100	HPV+: Curative 93.3%, palliative 6.7%, Surg +/- RT 27.7% RT 18.4%, CRT 27.6%, RT+LND+ RT (brachytherapy) 26.3%;<br><br>HPV- curative 84.4%,	Unselected Dutch cohort of patients with OPSCC treated with curative intent.	OS: defined as the time from the date of incidence (defined as the date on which the squamous cell carcinoma was histologically confirmed) to death (any cause).<br><br>PFS: defined as the time period from date of incidence to death or the first documented relapse, which was categorized as local-regional recurrence or distant metastases.<br>Median follow-up of patients who received treatment and remained alive was 4.33 years (range 0.1–12.1)

Study (D or EV)	Model and cancer location	Source of data (n, and n included in analysis)	Age (mean y (SD)), sex	Cancer stage	TNM	HPV status and measurement method	Smoking, alcohol, co-morbidities	Treatment	Inclusion criteria/ comments on cohort representa tiveness.	Outcomes, length of follow-up
	tongue 24.9%, soft palate 17.8%, oropharynx nos 18.9%.	parameter data).			60.9% Tx: 4.0% N0: 39.3% N1-3: 60.4% Nx: 0.3%		UY 25.9%, 111-149 UY 7.7%, >149 UY 64.2%, unknown 2.2%<br>HPV+: ACE-27 score 0: 56.4%, 1:24.5%, 3:17.2%, unknown 0.6%. HPV-: 0:32.4%, 1:30.3%, 2:28.8%, 3:8.4%, unknown: 0.2%	palliative 15.5%, surg +/-RT 30.7%, RT 31%, CRT 28.8%, RT +LND+RT (brachythe rapy): 9.5%		
Rietbergen 2015, The Netherlands<br><br>*External validation*	RTOG-0129 RPA model<br><br>OPSCC (no further details)	Patients curatively treated 2000–2011 at Maastricht University Medical Center, The Netherlands; retrospective analysis; **n=235**	Mean 60.24, median 58.96<br>73.6% male	TNM vr NR<br><br>I-II: 20.4% III-IV: 79.6%	TNM vr NR<br><br>T1-2: 51.5% T3-4: 48.5% Tx=0 N0-N2a: 48.1% N2b-N3: 51.9% Nx:0	HPV+: 30.2% HPV-: 69.8%<br><br>Tumour p16 protein expression (immunohistoc hemical analysis); Tumour HPV DNA detection (PCR)	0-10 PY: 18.7%, >10 PY: 81.3%) unknown: 0<br>Details collected but not reported.<br>ACE score 0-1: 71.9%, 2-3: 24.2%, unknown 3.8%.	Surgery +/- RT: 30.2% RT: 46% CRT: 19.1% Other: 4.7%	Single Dutch institution. Patients treated with curative intent.	OS: defined as time from date of incidence (defined as the date on which the squamous cell carcinoma was histologically confirmed) to death (any cause).<br><br>PFS: defined as time period from date of incidence to death or the first documented relapse that was categorised as local–regional recurrence or distant metastases.<br>Mean/median follow-up time not stated. Up to 5 years.

Study (D or EV)	Model and cancer location	Source of data (n, and n included in analysis)	Age (mean y (SD)), sex	Cancer stage	TNM	HPV status and measurement method	Smoking, alcohol, co-morbidities	Treatment	Inclusion criteria/ comments on cohort representa tiveness.	Outcomes, length of follow-up
Wang 2016, Taiwan<br><br>*External validation*	RTOG-0129 RPA model<br><br>OPSCC stage III-IV (57.5% tonsil, 23.9% tongue base, 10.6% pharyngeal wall, 8.0% soft palate)	Patients with non-metastatic stage III–IV OPSCC who had completed definite therapy June 2006 -Jan 2013 at one hospital; retrospective analysis; n=130; **n=113** (87%) in analysis.	Range 28-86, 52.2% >50 years<br>90.3% male	TNM 7th ed<br><br>III: 10.6%<br>IVa: 64.6%<br>IVb: 24.8%	TNM 7th ed<br><br>T1-T3: 44.2%<br>T4:55.8 %<br>N0-N2a: 23.0%,<br>N2b-N3: 77.0%	P16+: 24.8%<br>P16-: 75.2%<br><br>Tumour p16 protein expression (immunohistoc hemical analysis)	72.6% smokers<br>No details on alcohol.<br>No details on co-morbidity.	Cisplatin-based CCRT: 75.2%, induction chemother apy and CCRT: 18.6%, cetuximab bio-radiothera py: 4.4%, radiothera py alone: 1.8%.	Single Taiwanese hospital. Patients who had cancer and achieved complete remission for more than three years without CT or RT were eligible for the current study. Patients were excluded if they had been previously treated for OPSCC at other institutions or had a positive history of malignancie s for which they had	OS: not defined.<br><br>DSS: not defined.<br><br>At least 24 months or until death. Median follow-up time 27.6 months (range: 5.2–98.0 months).

**Study (D or EV)**	**Model and cancer location**	**Source of data (n, and n included in analysis)**	**Age (mean y (SD)), sex**	**Cancer stage**	**TNM**	**HPV status and measurement method**	**Smoking, alcohol, co-morbidities**	**Treatment**	**Inclusion criteria/ comments on cohort representa tiveness.**	**Outcomes, length of follow-up**
									been treated with CT or RT.	
Fakhry 2017, US<br><br>*Developme nt cohort (for Fakhry model), used for external validation of RTOG-0129 model*	RTOG-0129 RPA model<br><br>OPSCC (no further details)	Patients from RTOG 0129 and 0522 clinical trials (2002-2005 and 2005-2009), not consecutive, **n=493**	Mean age NR; 27.6% ≤50 yrs, 72.4% >50 yrs<br><br>87.4% male	All Stage III-IV as per the original RTOG studies.	TNM 7th and 8th eds<br><br>T2-3: 72.0%<br>T4: 28.0%<br>N0-2b (TNM7) or N0-N1 (TNM8): 66.5%<br>N2c-3 (TNM7) or N2-N3 (TNM8): 33.5%	P16 +: 73.6%<br>P16-: 26.4%<br><br>Tumour p16 protein expression (immunohistoc hemical analysis)	≤10 PYs: 47.3%, >10 PYs: 52.7%<br><br>Not reported<br><br>Zubrod/ECOG/ WHO PS: PS0: 69.0%; PS1: 31.0%	All patients curative RT+CT.	Patients from two US trials (NRG Oncology RTOG 0129 and 0522). Eligible patients had untreated, pathological ly confirmed, AJCC 5th edition (RTOG 0129) or 6th edition (RTOG 0522) stage III to IV7 head and neck squamous cell carcinoma, Zubrod performanc	OS: defined as time from date of randomisation to death from any cause.<br><br>PFS: defined as from date of randomization to local, regional, or distant progression or death from any cause.<br><br>Median: 5.7 years (95% CI, 5.2 to 6.0 years)

Study (D or EV)	Model and cancer location	Source of data (n, and n included in analysis)	Age (mean y (SD)), sex	Cancer stage	TNM	HPV status and measurement method	Smoking, alcohol, co-morbidities	Treatment	Inclusion criteria/ comments on cohort representa tiveness.	Outcomes, length of follow-up
									e status of 0 to 1, age ≥ 18 years, and adequate bone marrow, hepatic, and renal function.	
Deschuymer 2018, Belgium<br><br>*External validation*	RTOG-0129 RPA model<br><br>OPSCC (no further details)	All non-metastatic OPSCC patients treated with primary RT in a single institution Jan 2004 -July 2017; retrospective analysis; n=333; **n=258** (77%) in analysis.	HPV+ mean (IQR) 63.6 (54.9-70.1); HPV- 59.7 (54.0-65.8).<br>79.2% male.	TNM 7th ed<br><br>HPV+:<br>I: 0.%<br>II:10.0%<br>III: 11.0%<br>IVa: 68.0%<br>IVb: 11.0%<br>HPV-:<br>I: 0.63%<br>II:11.88%<br>III:16.25 %<br>IVa: 57.5%<br>IVb: 13.75%	TNM 7th ed<br><br>HPV+:<br>T1: 12.0%<br>T2: 41.0%<br>T3: 19.0%<br>T4a: 23.0%<br>T4b: 5.0%<br>N0: 17.0%<br>N1: 10.0%<br>N2a: 5.0%<br>N2b: 42.0%,	HPV+: 38.5%<br>HPV-: 61.5%<br><br>Tumour p16 protein expression (immunohistochemical analysis)	HPV+: mean PY 22.6 (21.65), never smoker 27.0%, ≤10 PY 14.0%, >10 PY 57.0%, unknown 3.0%. HPV-: mean PY 38.8 (22.12), never smoker 1.88%, ≤10 PY 5.0%, >10 PY 91.87%, unknown 1.25%.<br>No details on alcohol.<br>HPV+: ACE27 0 : 31.0%, 1: 37.0%, 2: 24.0%, 3:8.0%.	All primary radiotherapy. HPV+ no systematic treatment 31.0%, cisplatin 66.0%, EGFR inhibitor 3.0%. HPV- no systematic treatment 28.75%, cisplatin 60.63%, EGFR inhibitor 17.0%.	Single Belgian institution. Included only patients treated with curative primary RT, excluding stage IV (8th Ed) and excluding patients treated with primary surgery.	OS: calculated from the date of histological diagnosis to the date of death from any cause.<br><br>LRC: calculated from the date of histological diagnosis to the date of locoregional relapse (tumour at the primary site or regional nodes).<br><br>DMC: calculated from the date of histological diagnosis to the date of distant metastases.<br>Median follow-up 63.7 months (IQR 30.0; 99.9).

Study (D or EV)	Model and cancer location	Source of data (n, and n included in analysis)	Age (mean y (SD)), sex	Cancer stage	TNM	HPV status and measurement method	Smoking, alcohol, co-morbidities	Treatment	Inclusion criteria/ comments on cohort representa tiveness.	Outcomes, length of follow-up
					N2c:20.0%<br>N3: 6.0%.<br><br>HPV-:<br>T1: 0%<br>T2: 33.13%<br>T3: 22.5%<br>T4a: 26.25%<br>T4b:11.88%<br>N0: 23.13%<br>N1: 17.5%<br><br>N2a: 2.5%<br>N2b: 26.88%<br>N2c: 28.13%<br>N3: 1.88%		HPV-: ACE27 0: 16.25%, 1:40.0%, 2:23.75%, 3:20.0%.			

Study (D or EV)	Model and cancer location	Source of data (n, and n included in analysis)	Age (mean y (SD)), sex	Cancer stage	TNM	HPV status and measurement method	Smoking, alcohol, co-morbidities	Treatment	Inclusion criteria/ comments on cohort representa tiveness.	Outcomes, length of follow-up
Rios-Velaquez 2014, The Netherlands<br><br>*External validation Cohort 1*	RTOG-0129 RPA model<br><br>OPSCC (Tonsillar fossa (36.9%), base of tongue (29.8%), oropharynx overlap (25.6%), soft palate 7.7%))	Consecutively treated patients (Maastro Clinic) Jan 2000-Oct 2011; retrospective analysis; **n=168**	Median 59 (range 43-83)<br><br>74.4% male	TNM vr NR<br><br>I–IVb (no further details)	TNM vr NR<br><br>T1: 14.9%<br>T2: 27.4%<br>T3: 22.6%<br>T4: 35.1%<br>N0: 34.5%<br>N1: 17.3%<br>N2: 44.1%<br>N3: 3.6%<br>Nx: 0.6%	P16+: 34.5%<br>P16- : 64.3%<br>P16 unknown: 1.25%<br><br>HPV_DNA+:30.4%<br>HPV_DNA-: 69.6%<br><br>Tumour p16 protein expression (immunohistochemical analysis); Tumour HPV-16 DNA detection (PCR)	Median 30PY (range 0-100)<br><br>Median 134 units/years (range 0-660)<br><br>ACE 27<br>None: 33.3%<br>Mild: 41.1%<br>Moderate: 19%<br>Severe: 6.5%	RT only: 67.9%<br>CRT: 32.1%	Single Dutch institution. Patients treated with curative intent.	OS: defined as the time from starting radiotherapy to death from any cause.<br><br>PFS: defined as the time from starting radiotherapy to time of first documented recurrence at any site (locoregional or metastasis) or death from any cause.<br><br>Median 26 months (range 2.5–127.2) overall.<br><br>Median 37.5 months (range 6.4–127.2) for patients alive at last follow-up.
Rios-Velaquez 2014, The Netherlands<br><br>*External validation Cohort 2*	RTOG-0129 RPA model<br><br>OPSCC (Tonsillar fossa (38.1%), base of tongue (29.1%), oropharynx	Consecutively treated patients (VU University Medical Center) Jan 2000-Dec 2006; retrospective analysis; **n=189**	Median 60 (range 43– 93)<br>64.6% male	TNM vr NR<br><br>I–IVb (no further details)	TNM vr NR<br><br>T1: 13.8%<br>T2: 28%<br>T3: 33.3%<br>T4: 24.9%<br>N0:	P16+: 16.9%<br>P16- : 82%<br>P16 unknown: 1.1%<br><br>HPV DNA+: 18%<br>HPV DNA-: 82%<br><br>Tumour p16 protein	Median 32 PY (range 0-100)<br><br>Median 170 units/years (range 0-350)<br><br>ACE 27<br>None: 35.4%<br>Mild: 29.1%	RT only: 60.8%<br>CRT: 39.2%	Single Dutch institution. Patients treated with curative intent.	OS: defined as the time from starting radiotherapy to death from any cause.<br><br>PFS: defined as the time from starting radiotherapy to time of first documented recurrence at any site (locoregional or metastasis) or death from any cause.

Study (D or EV)	Model and cancer location	Source of data (n, and n included in analysis)	Age (mean y (SD)), sex	Cancer stage	TNM	HPV status and measurement method	Smoking, alcohol, co-morbidities	Treatment	Inclusion criteria/ comments on cohort representa tiveness.	Outcomes, length of follow-up
	overlap (20.1%), soft palate 12.7%))				43.9% N1: 12.2% N2: 41.8% N3: 2.1% Nx: 0%	expression (immunohistoc hemical analysis); Tumour HPV-16 DNA detection (PCR)	Moderate: 29.6% Severe: 5.8%			Not reported.
**Rietbergen 2013 risk stratification model**										
Rietbergen 2013, The Netherlands<br><br>*Developme nt*	Rietbergen 2013 model<br><br>OPSCC HPV+: tonsil 58.9%, base of tongue 31.3%, soft palate 5.5%, oropharynx nos 4.3%; HPV-: tonsil	All patients with an OPSCC diagnosed at two Dutch University hospitals Jan 2000 - Dec 2006; retrospective analysis; n=906; **n=721** in analysis (those treated with curative	HPV+: mean age at diagnosis 60.53 (median 58.14), HPV-: mean 60.94 (median 59.67).<br>67% male	TNM vr NR<br><br>I-IV	TNM vr NR<br><br>HPV+: T1-2: 54% T3-4: 46% Tx: 0 N0: 14.7% N1-3: 85.3% Nx: 0<br><br>HPV-:	HPV+: 19.4% HPV-: 76.8%<br><br>*NB based on n=809*<br><br>Tumour p16 protein expression (immunohistoc hemical analysis); Tumour HPV DNA detection (PCR)	HPV+: 0-10 pack years 40.5%, 11-24 PY 16.0%, >24 PY 42.9%, unknown 0.6%. HPV-: 0-10 PY 6.3%, 11-24 PY 9.3%, >24 PY 83.1%, unknown 1.2%.<br>HPV+: 0-100 unit yrs 68.7%, 111-149 UY 9.2%, >149 UY 21.5%,	HPV+: Curative 93.3%, palliative 6.7%, Surg +/- RT 27.7%, RT 18.4%, CRT 27.6%, RT+LND+ RT (brachythe rapy) 26.3%;	Unselected Dutch cohort of patients with OPSCC treated with curative intent.	OS: defined as the time from the date of incidence (defined as the date on which the squamous cell carcinoma was histologically confirmed) to death (any cause).<br><br>PFS: defined as the time period from date of incidence to death or the first documented relapse, which was categorized as local-regional recurrence or distant metastases.<br>Median follow-up of patients who received treatment and remained alive was 4.33 years (range 0.1–12.1)

Study (D or EV)	Model and cancer location	Source of data (n, and n included in analysis)	Age (mean y (SD)), sex	Cancer stage	TNM	HPV status and measurement method	Smoking, alcohol, co-morbidities	Treatment	Inclusion criteria/ comments on cohort representa tiveness.	Outcomes, length of follow-up
	38.4%, base of tongue 24.9%, soft palate 17.8%, oropharynx nos 18.9%.	intent and with model parameter data).	*NB based on n=809*		T1-2: 39.1% T3-4: 60.9% Tx: 4.0% N0: 39.3% N1-3: 60.4% Nx: 0.3%		unknown 0.6%. HPV-: 0-100 UY 25.9%, 111-149 UY 7.7%, >149 UY 64.2%, unknown 2.2%<br>HPV+: ACE-27 score 0: 56.4%, 1:24.5%, 3:17.2%, unknown 0.6%. HPV-: 0:32.4%, 1:30.3%, 2:28.8%, 3:8.4%, unknown: 0.2%	HPV- curative 84.4%, palliative 15.5%, surg +/-RT 30.7%, RT 31%, CRT 28.8%, RT +LND+RT (brachythe rapy): 9.5%		
Rietbergen 2015, The Netherlands<br><br>*External validation*	Rietbergen 2013 model<br><br>OPSCC (no further details)	Patients curatively treated 2000–2011 at Maastricht University Medical Center; retrospective analysis; **n=235**	Mean 60.24, median 58.96<br>73.6% male	TNM vr NR<br><br>I-II: 20.4% III-IV: 79.6%	TNM vr NR<br><br>T1-2: 51.5% T3-4: 48.5% Tx=0 N0-N2a: 48.1%	HPV+: 30.2% HPV-: 69.8%<br><br>Tumour p16 protein expression (immunohistoc hemical analysis); Tumour HPV DNA detection (PCR)	0-10 PY: 18.7%, >10 PY: 81.3%) unknown: 0<br>Details collected but not reported.<br>ACE score 0-1: 71.9%, 2-3: 24.2%, unknown 3.8%.	Surgery + RT: 30.2%; RT: 46%; CRT: 19.1%; other: 4.7%	Single Dutch institution. Patients treated with curative intent.	OS: defined as time from date of incidence (defined as the date on which the squamous cell carcinoma was histologically confirmed) to death (any cause).<br><br>PFS: defined as time period from date of incidence to death or the first documented relapse that was categorised as local–regional recurrence or distant metastases.

Study (D or EV)	Model and cancer location	Source of data (n, and n included in analysis)	Age (mean y (SD)), sex	Cancer stage	TNM	HPV status and measurement method	Smoking, alcohol, co-morbidities	Treatment	Inclusion criteria/ comments on cohort representativeness.	Outcomes, length of follow-up
					N2b-N3: 51.9% Nx:0					Mean/median follow-up time not stated. Up to 5 years.
Deschuymer 2018, Belgium<br><br>*External validation*	RTOG-0129 RPA model<br><br>OPSCC (no further details)	All non-metastatic OPSCC patients treated with primary RT in a single institution Jan 2004 -July 2017; retrospective analysis; n=333; **n=258** (77%) in analysis.	HPV+ mean (IQR) 63.6 (54.9-70.1); HPV- 59.7 (54.0-65.8).<br>79.2% male.	TNM 7th ed<br><br>HPV+:<br>I: 0.%<br>II:10.0%<br>III: 11.0%<br>IVa: 68.0%<br>IVb: 11.0%<br>HPV-:<br>I: 0.63%<br>II:11.88%<br>III:16.25 % IVa: 57.5%<br>IVb: 13.75%	TNM 7th ed<br><br>HPV+:<br>T1: 12.0%<br>T2: 41.0%<br>T3: 19.0%<br>T4a: 23.0%<br>T4b: 5.0%<br>N0: 17.0%<br>N1: 10.0%<br>N2a: 5.0%<br>N2b: 42.0%,<br>N2c:20.0%<br>N3: 6.0%.	HPV+: 38.5%<br>HPV-: 61.5%<br><br>Tumour p16 protein expression (immunohistochemical analysis)	HPV+: mean PY 22.6 (21.65), never smoker 27.0%, ≤10 PY 14.0%, >10 PY 57.0%, unknown 3.0%. HPV-: mean PY 38.8 (22.12), never smoker 1.88%, ≤10 PY 5.0%, >10 PY 91.87%, unknown 1.25%.<br>No details on alcohol.<br>HPV+: ACE27 0 : 31.0%, 1: 37.0%, 2: 24.0%, 3:8.0%. HPV-: ACE27 0: 16.25%, 1:40.0%, 2:23.75%, 3:20.0%.	All primary RT. HPV+ no systematic treatment 31.0%, cisplatin 66.0%, EGFR inhibitor 3.0%. HPV- no systematic treatment 28.75%, cisplatin 60.63%, EGFR inhibitor 17.0%.	Single Belgian institution. Included only patients treated with curative primary RT, excluding stage IV (8th Ed) and excluding patients treated with primary surgery.	OS: calculated from the date of histological diagnosis to the date of death from any cause.<br><br>LRC: calculated from the date of histological diagnosis to the date of locoregional relapse (tumour at the primary site or regional nodes).<br><br>DMC: calculated from the date of histological diagnosis to the date of distant metastases.<br>Median follow-up 63.7 months (IQR 30.0; 99.9).

Study<br>(D or EV)	Model and cancer location	Source of data (n, and n included in analysis)	Age (mean y (SD)), sex	Cancer stage	TNM	HPV status and measurement method	Smoking, alcohol, co-morbidities	Treatment	Inclusion criteria/ comments on cohort representativeness.	Outcomes, length of follow-up
					HPV-:<br>T1: 0%<br>T2: 33.13%<br>T3: 22.5%<br>T4a: 26.25%<br>T4b:11.88%<br>N0: 23.13%<br>N1: 17.5%<br>N2a: 2.5%<br>N2b: 26.88%<br>N2c: 28.13%<br>N3: 1.88%					
**Huang 2015 RPA and AHR risk stratification models**										
Huang 2015, Canada<br><br>*Development*	Huang 2015 RPA model and AHR model	Consecutive patients treated 2000-2010 at one institution; retrospective analysis; **n=573**	Median 57.8<br><br>79% male	TNM 7th ed<br><br>I: 1.4%<br>II: 4.4%<br>III: 13.8%<br>IV: 80.5%	TNM 7th ed<br><br>T1: 20.4%<br>T2: 34.6%	100% HPV + (p16 +/- staining)<br><br>Tumour p16 protein expression (immunohistoc	Median 40 PYs<br><br>Not reported<br><br>Not reported	All radiotherapy<br>CRT: 50%	Unselected patient population with HPV-related OPC from a single Canadian	OS: defined as the duration between “random assignment” and death.<br><br>Median 5·1 years

Study (D or EV)	Model and cancer location	Source of data (n, and n included in analysis)	Age (mean y (SD)), sex	Cancer stage	TNM	HPV status and measurement method	Smoking, alcohol, co-morbidities	Treatment	Inclusion criteria/ comments on cohort representativeness.	Outcomes, length of follow-up
	OPSSC (HPV+ only)	NB based on 1,108 patients, 573HPV+, 237 HPV- and 298 (27%) with unknown status			T3: 26.9 %<br>T4: 18.2%<br>N0: 12%<br>N1: 10.5%<br>N2a: 7.9%<br>N2b: 36.3%<br>N2c: 24.1%<br>N3: 9.3%	hemical analysis)			institution treating almost all patients in its region. Patients with nonmetastatic (M0) OPC treated with definitive RT or CRT.	
Keane 2016, US<br><br>*External validation*		Patients from SEER database, diagnosed 2004-2008; retrospective analysis; **n=8427**	Median 58 (21–96)<br><br>82.8% male.	TNM 6th ed<br><br>I: 5.9%<br>II: 8.9%<br>III: 22.6%<br>IVA: 57.5%<br>IVB: 5.2%	TNM 6th ed<br><br>T1: 25.3%<br>T2: 39.2%<br>T3: 12.5%<br>T4: 23.0%<br>N0: 21.9%<br>N1: 24.8%<br>N2a: 10.6%	HPV+ (no further information on method of HPV status determination).<br><br>Method not reported.	Not reported<br><br>Not reported<br><br>Not reported	Radiotherapy No: 14.4%<br>Yes: 85.6%<br>Radical surgery No: 72.1<br>Yes: 27.7%<br>Unknown: 0.02%.<br><br>No information on CT.	Patients with non-metastatic cancer from US SEER database. No information on CT, smoking, alcohol or co-morbidities. Unclear if cohort different to DEV one.	OS: defined as time from date of diagnosis to date of death.<br><br>HNC-specific mortality: defined as time from date of diagnosis to date of head and neck cancer related death.<br><br>Median follow-up 50 months

Study (D or EV)	Model and cancer location	Source of data (n, and n included in analysis)	Age (mean y (SD)), sex	Cancer stage	TNM	HPV status and measurement method	Smoking, alcohol, co-morbidities	Treatment	Inclusion criteria/ comments on cohort representa tiveness.	Outcomes, length of follow-up
					N2b: 24.7%<br>N2c: 12.9%<br>N3: 5.2%<br>All M0				Not possible to determine whether patients received definitive-intent surgery.	
O'Sullivan 2016, Canada<br><br>*External validation*		Consecutive patients treated 2000-2011 at six institutions; retrospective analysis; **n=1246**<br>NB based on patients with known HPV+ status	Median 56 (IQR 51–62)<br><br>86% male	TNM 7th ed<br><br>I: 1%<br>I: 3%<br>III: 13%<br>IVA: 76%<br>IVB: 7%	TNM 7th ed<br><br>T1: 30%<br>T2: 39%<br>T3: 19%<br>T4a: 11%<br>T4b: 1%<br>N0: 7%<br>N1: 12%<br>N2a: 12%<br>N2b: 41%<br>N2c: 22%	100% HPV + (p16 +/- staining)<br><br>Tumour p16 protein expression (immunohistoc hemical analysis); Tumour HPV DNA detection (in situ hybridization)	Median PYs 6 (IQR 0-25)<br><br>Not reported<br><br>Not reported	Surgery: 2%<br>RT: 98%<br>CT yes: 75%<br>CT no: 25%	Patients with non-metastatic oropharyng eal cancer from six institutions, one from Denmark, one from the Netherland s and four from the US. Patients with newly diagnosed non-metastatic (M0)	Risk of death: defined as risk of death from any cause from the date of diagnosis.<br><br>Median 4·6 years (IQR 3·1–5·5)

Study (D or EV)	Model and cancer location	Source of data (n, and n included in analysis)	Age (mean y (SD)), sex	Cancer stage	TNM	HPV status and measurement method	Smoking, alcohol, co-morbidities	Treatment	Inclusion criteria/ comments on cohort representa tiveness.	Outcomes, length of follow-up
					N3: 6%<br>All M0				oropharyngeal cancer undergoing either primary surgery or primary radiotherapy with or without chemotherapy. No explicit statement on treatment with curative intent.	
**O'Sullivan 2016 AHR and RPA risk stratification model (updated from Huang 2015)**										
O'Sullivan 2016, Canada<br><br>*Development*	O'Sullivan 2016 AHR model<br><br>OPSSC (HPV+ only)	Consecutive patients treated 2000-2011 at one institution; retrospective analysis, **n=661**<br>NB based on patients with known HPV+ status	Median 57 (IQR 51–65)<br><br>80% male	TNM 7th ed<br><br>I: 1%<br>II: 4%<br>III: 13%<br>IVA: 68%<br>IVB: 13%	TNM 7th ed<br><br>T1: 20%<br>T2: 35%<br>T3: 26%<br>T4a: 13%	100% HPV + (p16 +/- staining)<br><br>Tumour p16 protein expression (immunohistochemical analysis); Tumour HPV DNA detection	Median PYs 15 (IQR 0-30)<br><br>Not reported<br><br>Not reported	Surgery: 1%<br>RT: 99%<br>CT yes: 49%<br>CT no: 51%	Patients with non-metastatic oropharyngeal cancer from one Canadian institution. Patients with newly diagnosed non-	Risk of death: defined as risk of death from any cause from the date of diagnosis.<br><br>Median 5·5 years (IQR 3·2–6·6)

Study (D or EV)	Model and cancer location	Source of data (n, and n included in analysis)	Age (mean y (SD)), sex	Cancer stage	TNM	HPV status and measurement method	Smoking, alcohol, co-morbidities	Treatment	Inclusion criteria/ comments on cohort representa tiveness.	Outcomes, length of follow-up
					T4b: 5%<br>N0: 12%<br>N1: 10%<br>N2a: 8%<br>N2b: 36%<br>N2c: 25%<br>N3: 8%<br>All M0	(in situ hybridisation).			metastatic (M0) oropharyngeal cancer undergoing either primary surgery or primary RT with or without CT.	
O'Sullivan 2016, Canada<br><br>*External validation*		Consecutive patients treated 2000-2011 at six institutions; retrospective analysis; **n=1246**<br>NB based on patients with known HPV+ status	Median 56 (IQR 51–62)<br><br>86% male	TNM 7th ed<br><br>I: 1%<br>I: 3%<br>III: 13%<br>IVA: 76%<br>IVB: 7%	TNM 7th ed<br><br>T1: 30%<br>T2: 39%<br>T3: 19%<br>T4a: 11%<br>T4b: 1%<br>N0: 7%<br>N1: 12%<br>N2a: 12%	100% HPV + (p16 +/- staining)<br><br>Tumour p16 protein expression (immunohistochemical analysis); Tumour HPV DNA detection (in situ hybridisation).	Median PYs 6 (IQR 0-25)<br><br>Not reported<br><br>Not reported	Surgery: 2%<br>RT: 98%<br>CT yes: 75%<br>CT no: 25%	Patients with non-metastatic oropharyngeal cancer from six institutions, one from Denmark, one from the Netherlands and four from the US. Patients with newly diagnosed non-	Risk of death: defined as risk of death from any cause from the date of diagnosis.<br><br>Median 4·6 years (IQR 3·1–5·5)

Study (D or EV)	Model and cancer location	Source of data (n, and n included in analysis)	Age (mean y (SD)), sex	Cancer stage	TNM	HPV status and measurement method	Smoking, alcohol, co-morbidities	Treatment	Inclusion criteria/ comments on cohort representativeness.	Outcomes, length of follow-up
					N2b: 41%<br>N2c: 22%<br>N3: 6%<br>All M0				metastatic (M0) oropharyngeal cancer undergoing either primary surgery or primary RT with or without CT.	
**Alabi 2022 Risk stratification model**										
Alabi 2022<br><br>*Development*	ProgTOOL<br><br>OPSCC (Tonsil 55.1%), base of tongue (37.2%), oropharynx (6.9%), vallecular 0.8%))	Patients from SEER database 2010-2015; retrospective analysis; **n=3164** Patients with known clinical and pathological characteristics.	Median 61 (SD 10.4, range 20-85; mean 61.4)<br><br>79.8% male	Not reported	TNM 7th ed<br><br>T1: 27.6%<br>T2: 40.4%<br>T3: 18.3%<br>T4: 13.6%<br>N0: 44.8%<br>N1: 45%<br>N2: 0%	HPV+: 63.9%<br>HPV-: 36.1%<br><br>No further information on method of HPV status determination.	Not reported<br><br>Not reported<br><br>Not reported	Surgery: 18.3%<br>Surgery + RT: 40.1%<br>Surgery + CRT: 18.3%<br>CRT: 13.1%<br>No treatment: 12.1%	Included all cases with OPSCC with known clinical and pathologic characteristics.	OS: defined as the time period from the beginning (or end) of treatment until the patients die of any cause.<br><br>Median 49, mean 49.4, SD 27.2, range 0 to 107 months.

Study (D or EV)	Model and cancer location	Source of data (n, and n included in analysis)	Age (mean y (SD)), sex	Cancer stage	TNM	HPV status and measurement method	Smoking, alcohol, co-morbidities	Treatment	Inclusion criteria/ comments on cohort representa tiveness.	Outcomes, length of follow-up
					N3: 10.2%<br>M0: 97.5%<br>M1:2.5 %					
Alabi 2023<br><br>*External validation*	ProgTOOL<br><br>OPSCC (Tonsil 58.9%), base of tongue (28.2%), oropharynx (12.9%).	Case of OPSCC from electronic patient records at Helsinki University Hospital; retrospective analysis; **n=163** Patients with known clinical and pathological characteristic s.	Median 62 (SD 9.1, range 37-85, mean 61.4)<br><br>75.5% male	Not reported	TNM 7th ed<br><br>T1: 32.5%<br>T2: 32.5%<br>T3: 10.4%<br>T4: 24.5%<br>N0: 15.3%<br>N1: 9.8%<br>N2: 73.6%<br>N3: 1.2%<br>M0: 98.8%<br>M1:1.2 %	HPV+: 74.2%<br>HPV-: 25.8%<br><br>No further information on method of HPV status determination.	Not reported<br><br>Not reported<br><br>Not reported	Surgery: 4.9%<br>Surgery + RT: 14.1%<br>Surgery + CRT: 21.5%<br>CRT: 51.5%<br>No treatment: 8.0%	Included cases with OPSCC with known clinical and pathologic characterist ics.	OS: not defined<br><br>Median 50, mean 45.8, SD 19.9, range 0 to 88 months.

Model characteristics and performance

Ang 2010, US<br>*Development*	**RTOG-0129 RPA model**<br><br>HPV status: positive vs negative<br>Number of pack-years of tobacco smoking: ≤10 vs >10<br>Nodal/tumour stage: N0 to N2a vs N2b to N3 for HPV-positive tumours<br>OR: tumour stage (T2 or T3 vs T4), for HPV-negative tumours<br><br>*Flowchart presented on how to assign low, intermediate and high risk.*	*Model discrimination (c-index)*: NR<br>*Model calibration*: NR<br>*Other model performance measures*: patients classified as having low, intermediate or high risk of 3-yr OS using RPA.
Granata 2012, Italy<br>*External validation*		*Model discrimination (c-index)*: 2yr OS: c-index (adjusted for optimism) 0.70 (no CI stated)<br>*Model calibration*: Observed event rate for OS in external validation set was slightly higher than the observed event rate for OS in the DEV cohort for all three predicted risk groups.<br>*Other model performance measures*: NR
Rietbergen 2013, The Netherlands<br>*External validation*		*Model discrimination (c-index)*: OS: c-index 0.58 (95% CI 0.56-0.61)<br>*Model calibration*: NR<br>*Other model performance measures*: NR
Rietbergen 2015, The Netherlands<br>*External validation*		*Model discrimination (c-index)*: 5 yr OS: c-index 0.65 (95% CI 0.59, 0.70); 5 yr PFS: c-index 0.61 (95% CI 0.54, 0.68)<br>*Model calibration*: NR<br>*Other model performance measures*: NR
Wang 2016, Taiwan<br>*External validation*		*Model discrimination (c-index)*: OS: c-index 0.67 (CI NR); DSS: c-index 0.68 (CI NR)<br>*Model calibration*: NR<br>*Other model performance measures*: NR
Fakhry 2017, US<br>*External validation*		*Model discrimination (c-index)*: OS: c-index 0.71 (95% CI 0.66, 0.76)<br>*Model calibration*: NR<br>*Other model performance measures*: NR
Deschuymer 2018, Belgium<br>*External validation*		*Model discrimination (c-index)*: OS: c-index 0.57 (95% CI 0.52, 0.62); OS HPV+ group only: c-index 0.62 (95% CI 0.51, 0.73); c-index not reported for LRC or DMD<br>*Model calibration*: NR<br>*Other model performance measures*: NR
Rios-Velazquez 2014<br>*External validation (Maastro cohort)*		*Model discrimination (c-index)*: OS c-index 0.76 (95% CI 0.65, 0.80); PFS: c-index 0.74 (95% CI 0.70, 0.82)<br>*Model calibration*: NR<br>*Other model performance measures*: NR
Rios-Velazquez 2014<br>*External validation (VUMC cohort)*		*Model discrimination (c-index)*: OS: c-index 0.72 (95% CI 0.64, 0.78); PFS: c-index 0.64 (95% CI 0.59, 0.72)<br>*Model calibration*: NR<br>*Other model performance measures*: NR
Rietbergen 2013, the Netherlands	**Rietbergen 2013 model**<br>HPV status: positive vs negative	*Model discrimination (c-index)*: OS: c-index 0.68 (95% CI 0.65-0.71)<br>*Model calibration*: NR

Development	<u>Comorbidity</u>: ACE 0 vs ACE 1-2 vs ACE 3 (HPV-); ACE 0-1 vs ACE 2-3 (HPV+)<br><u>Nodal stage (in HPV-):</u> N0-N2a vs N2b-N3<br><u>Tumour stage (in HPV+ with ACE 0)</u>: T1-2 vs T3-4<br><br>*Flowchart presented on how to assign low, intermediate and high risk.*	*Other model performance measures*: NR
Rietbergen 2015<br><br>*External validation*		*Model discrimination (c-index)*: 5 yr OS: c-index 0.69 (95% CI 0.63, 0.75); 5 yr PFS: c-index 0.66 (95% CI 0.59-0.74)<br>*Model calibration*: NR<br>*Other model performance measures*: NR
Deschuymer 2018, Belgium<br><br>*External validation*		*Model discrimination (c-index)*: OS: c-index 0.58 (0.53, 0.64); OS: c-index HPV+ group only: 0.62 (0.52, 0.73); LRC, DMC: NR<br>*Model calibration*: NR<br>*Other model performance measures*: NR
Huang 2015, Canada<br><br>*Development*	**Huang 2015 RPA model**<br><br>Recursive partitioning analysis model<br><u>RPA-I</u>: T1-3N0-2b<br><u>RPA-II</u>: T1-3N2c<br><u>RPA-III</u>: T4 or N3	*Model discrimination (c-index)*: NR<br>*Model calibration*: NR<br>*Other model performance measures*: Based on an overall score derived from the hazard consistency score, the hazard discrimination score, explained variance score, hazard difference score and sample size balance score, the RPA risk prediction model performed best, followed by the AHR model and then the AJCC/UICC 7th edition.<br>*(NB Further model developed based on RPA stage, age and smoking developed in Huang 2015 but not validated)*
Keane 2016, US<br><br>*External validation*		*Model discrimination (c-index)*: OS c-index 0.60 (95% CI 0.59, 0.61); HNC-specific mortality: c-index 0.62 (95% CI 0.61, 0.63); AJCC staging system: OS: c-index 0.54 (95% CI 0.53, 0.55); HNC-specific mortality: c-index 0.55 (95% CI 0.54, 0.57)<br>*Model calibration*: NR<br>*Other model performance measures*: NR
Huang 2015, Canada<br><br>*Development*	**Huang 2015 AHR model**<br><br>Adjusted hazard ratio derived model<br><u>AHR-I</u>: T1N0-N2b or T2N0-N2a<br><u>AHR-II</u>: T1N2c, T2N2b-N2c, or T3N0-N2b<br><u>AHR-III</u>: T1-2N3, T3N2c, or T4N0-N2a<br><u>AHR-IVA</u>: T3N3 or T4N2b-N3<br><u>AHR-IVB</u>: M1	*Model discrimination (c-index)*: NR<br>*Model calibration*: NR<br>*Other model performance measures*: Based on an overall score derived from the hazard consistency score, the hazard discrimination score, explained variance score, hazard difference score and sample size balance score, the RPA risk prediction model performed best, followed by the AHR model and then the AJCC/UICC 7th edition.<br>*(NB Further model based on RPA stage, age and smoking developed in Huang 2015 but not validated)*
O'Sullivan 2016, Canada<br><br>*External validation (in both training cohort and validation cohort)*		*Model discrimination (c-index)*: NR<br>*Model calibration*: NR<br>*Other model performance measures*: Based on an overall score derived from the hazard consistency score, the hazard discrimination score, explained variance score, hazard difference score and sample size balance score, the AHR risk classification performed best, followed by the RPA model and then the AJCC/UICC 7th edition.

O’Sullivan 2016, Canada<br><br>*Development*	**O’Sullivan 2016 AHR model**<br><br>Adjusted hazard ratio derived model<br>AHR-New I: T1–T2N0–N2b<br>AHR-New II: T1–T2N2c or T3N0–N2c<br>AHR-New III: T4 or N3<br><br>**O’Sullivan 2016 RPA model**<br>RPA stage I: T1–T3N0–N2b<br>RPA stage II: T1–T3N2c<br>RPA stage III: T4 or N3	*Model discrimination (c-index)*: NR<br>*Model calibration*: NR<br>*Other model performance measures*: Based on an overall score derived from the hazard consistency score, the hazard discrimination score, explained variance score, hazard difference score and sample size balance score, the AHR (NEW) risk classification performed best (and as well as the AHR Original), followed by the RPA model and then the AJCC/UICC 7th edition.
O’Sullivan 2016, Canada<br><br>*External validation*		*Model discrimination (c-index)*: NR<br>*Model calibration*: NR<br>*Other model performance measures*: Based on an overall score derived from the hazard consistency score, the hazard discrimination score, explained variance score, hazard difference score and sample size balance score, the AHR (NEW) risk classification performed best, followed by AHR Original, the RPA model and then the AJCC/UICC 7th edition.
Alabi 2022<br><br>*Development*	**ProgTOOL**<br><br>Age<br>Sex: 0=male, 1=female<br>Ethnicity: 0=white, 1=black, 2=other<br>Marital status: 1=married, 0=single<br>Tumour grade: I-IV<br>HPV status: 0=negative, 1=positive<br>Site: 1=base of tongue, 2=oropharynx, 3=tonsils 4=n=vallecular<br>T-stage (AJCC 7th ed): 1-4<br>N=stage (AJCC 7th ed): 1-3<br>M-stage (AJCC 7th ed): 0-1<br>Surgery: 0=no, 1=yes<br>Surgery +radiotherapy: 0=no, 1=yes<br>Chemotherapy: 0=no, 1=yes<br>Disease free survival (months): 0-500<br><br>http://oncotelligence.com/	*Model discrimination (c-index)*: NR<br>*Model calibration*: NR<br>*Other model performance measures:* PPV: 0.97, NPV: 0.76, sensitivity: 0.89, specificity: 0.92, F1 score: 0.93, Accuracy: 89.9%, balanced accuracy: 86.3%, weighted accuracy: 92.5%, Matthews’ correlation coefficient: 0.77, weighted AUC: 0.929
Alabi 2023<br><br>*External validation*		*Model discrimination (c-index)*: NR<br>*Model calibration*: Slope not fitted so difficult to interpret. Accuracy of prediction appears to vary depending on lower/higher predicted probabilities.<br>*Other model performance measures:* PPV: 0.93, NPV: 0.89, sensitivity: 0.76, specificity: 0.97, F1 score: 0.84, Accuracy: 90.2%, balanced accuracy: 86.5%, Matthews’ correlation coefficient: 0.78, weighted AUC: 0.94, Brier score 0.06, Net Benefit value of model approximately 0.7 at 10% - 50% probability threshold.

Risk of bias summary

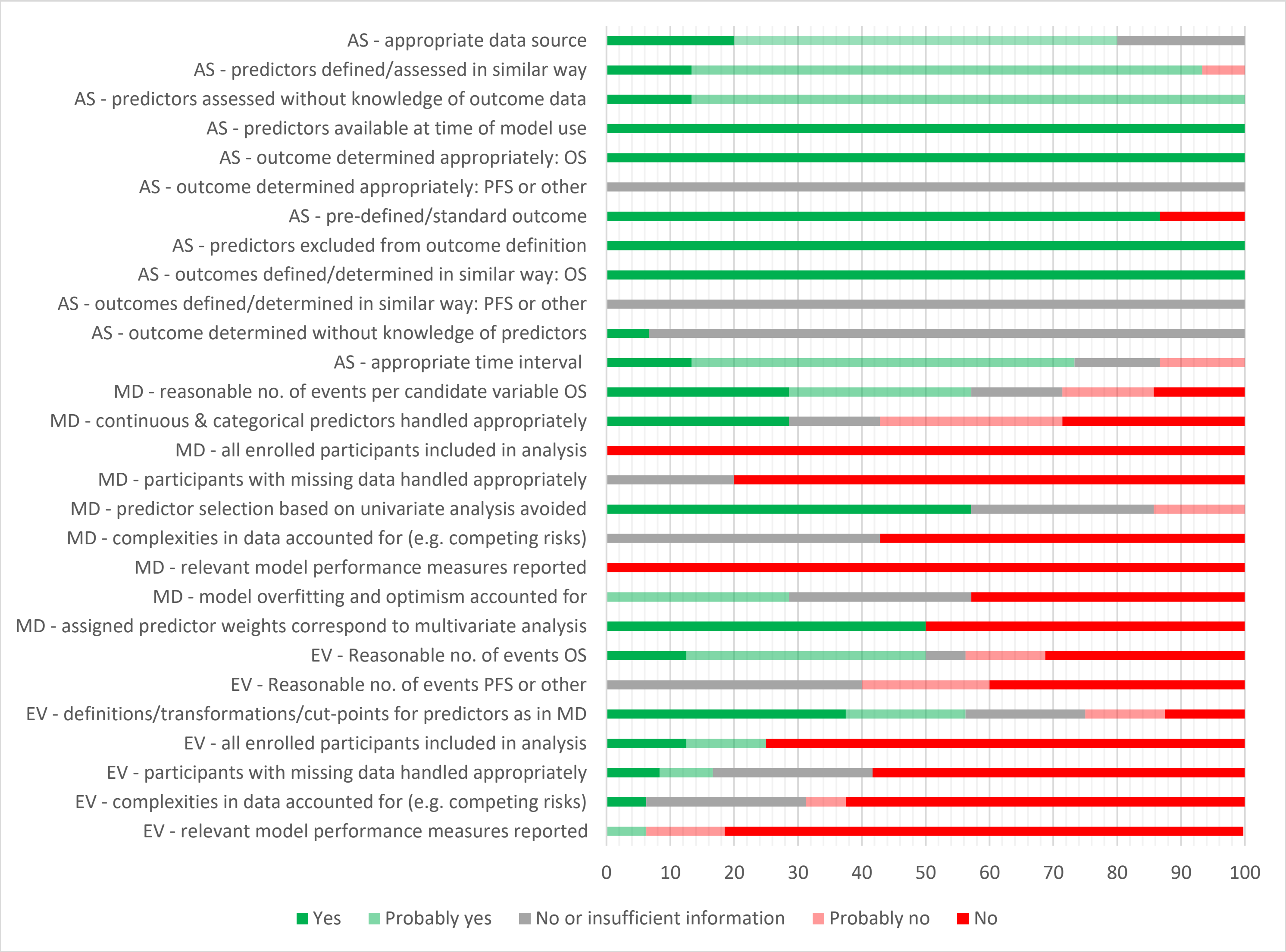

Figure: PROBAST summary Chart shows percentage of study cohorts meeting/not meeting criteria: Y=yes; PY=probably yes; NI=no or insufficient information; PN=probably no; N=no. AS=all study cohorts; MD=model development cohorts; EV=external validation cohorts. Numbers of cohorts contributing to the different criteria varies (e.g. as not all evaluations reporting both OS and PFS; the criterion *'participants with missing data handled appropriately'* is only applicable where there was missing data). Every evaluation counted for the analysis domain; some cohorts were used for evaluating more than one model. The criterion *'all enrolled participants included in analysis'* was answered with 'no' if participants were excluded on the basis of missing variable data.
