# Supplementary material for: Systematic review of prognostic models for predicting recurrence and survival in patients with treated oropharyngeal cancer
Source: BMJ Open. 2024 Dec 5;14(12):e090393. doi: 10.1136/bmjopen-2024-090393 (PMC11624838; doi:10.1136/bmjopen-2024-090393)
Supplement: online supplemental file 8 [file bmjopen-14-12-s008.pdf]

Supplementary material 8

Main population and study characteristics IPMs

Study (cohort)	Source of data (n, and n included in analysis)	Age (mean y (SD)), sex	Cancer stage	TNM	HPV status and measurement method	a. Smoking<br>b. Alcohol<br>c. Co-morbidities	Treatment	Inclusion criteria/ comments on cohort representativeness	Outcomes, length of follow-up
Beesley 2021, US, The Netherlands<br><br>***Development cohort***<br><br>***External validation of Fakhry 2017 nomogram and Grønhøj-Larsen 2016 OS nomogram***	Patients from the University of Michigan Jan 2003-Aug 2016; retrospective analysis; n=840	Median 58 (range 52-64.4); 85.1% male	Not reported	TNM 8th ed<br><br>T1: 22.5%, T2: 33.2%, T3: 16.2%, T4: 27.7% unknown: 0.4%<br>N0: 11.9%, N1: 45.2%, N2: 21%, N3:9.2%, Unknown: 12.7%	p16+: 50.6%<br>p16-: 10.5%<br>Unknown: 38.9%<br><br>HPV DNA+: 58.3%<br>HPV DNA-10.9%<br>Unknown: 30.7%<br><br>Method not reported.	a. Never: 33%, former: 34.5%, current: 31.9%, unknown: 0.6%<br><br>b. Not reported.<br><br>c. ACE27 none: 24.4%, mild: 30.6%, moderate: 13.2%, severe: 5.2%, unknown: 26.5%.<br>ECOG performance status: 0: 48.3%<br>1-2: 6.2% Unknown: 45.5%	CRT: 77.1%<br>CT alone: 1.7%<br>RT alone: 4.8%<br>Surgery (+/- adjuvant therapy): 9.9%<br>Unknown: 6.5%	Single US institution.<br>Patients with a second concurrent cancer excluded.<br>Different to EV cohort in terms of treatment, smoking habits and p16 status.<br>No explicit statement on treatment with curative intent.	Locoregional recurrence: defined as biopsy-proven or clinically overt imaging identification of cancer recurrence at the primary tumour site or cervical lymph nodes.<br><br>Distant metastasis: defined as the identification of metastasis outside the head and neck by biopsy or clinically overt imaging.<br><br>Survival: defined as the minimum time from diagnosis to the date of death, loss to follow-up, or March 18, 2019.<br><br>Event free survival: not defined.<br><br>Up to 72 months. No details on mean/median length of follow-up.
Beesley 2021, US, The Netherlands<br><br>***External validation of Beesley 2021 model***	Patients from the Erasmus Medical Centre (The Netherlands) Jan 2000 - Dec 2006; retrospective analysis; n=447	Median 59.9 (range 53.7-67.8); 67.1% male	Not reported.	TNM 8th ed<br><br>T1: 10.5% T2: 31.1% T3: 34.9% T4: 23.3% Unknown: 0.2%<br>N0:32.4%<br>N1:23.9%<br>N2:36.2% N3: 6.7%	p16+: 18.8%<br>p16-: 81.2%<br>Unknown: 0%<br><br>Method not reported.	a. Never: 7.8%, former: 12.1%, current: 78.3%, unknown: 1.8%.<br><br>b. Not reported.<br><br>c. ACE27 none: 38.8%, mild: 30.6%, moderate: 23.7%, severe: 6.9%, unknown: 0.5%.	CRT: 27.3%<br>CT alone: 0%<br>RT alone 27.3%<br>Surgery (+/- adjuvant therapy): 36.9%<br>Unknown: 8.5%	Single Dutch institution.<br>Different to DEV cohort in terms of treatment, smoking habits and p16 status.<br>No explicit statement on treatment with curative intent.	

Study (cohort)	Source of data (n, and n included in analysis)	Age (mean y (SD)), sex	Cancer stage	TNM	HPV status and measurement method	a. Smoking<br>b. Alcohol<br>c. Co-morbidities	Treatment	Inclusion criteria/ comments on cohort representative ness	Outcomes, length of follow-up
Beesley 2019, US<br><br>***External validation of Fakhry 2017, Grønhøj-Larsen 2016 and Rios-Velazquez 2014 nomogram***	Patients from two overlapping datasets, University of Michigan; retrospective analysis; n=856	58.4 (9.65); 84.6% male	TNM 7th ed.<br>I: 2.1%<br>II: 4.4%<br>III: 11.4%<br>IV: 81.8%<br>Unknown: 0.1%<br>TNM 8th ed.<br>I: 32.1%<br>II: 13.2%<br>III: 18.4%<br>IV: 7.5%<br>Unknown: 28.6%	TNM 7th ed.: T1: 22.7%, T2: 33.1% T3: 16.1%, T4: 27.5%, Unknown: 0.3%<br>N0: 12.8%, N1: 10.5%, N2: 5.2%, N2a: 7.5%, N2b: 38.2%, N2c: 16.2%, N3: 9.3%	P16+: 50.2%<br>P16-: 10.1%<br>P16 unknown: 39.7%<br><br>HPV DNA+: 57.8%<br>HPV DNA: 11.2%<br>HPV DNA unknown: 30.1%<br><br>Method not reported.	a. Never: 33.0%, former: 34.6%, current: 31.6%, unknown: 0.5%<br><br>b. Not reported<br><br>c. ACE-27: None: 24.0%, mild: 30.2%, moderate: 13.0%, severe: 5.1%, unknown: 27.4%.	CRT: 76.0%<br>RT alone: 4.9%<br>Surgery + adjuvant CRT: 3.8%<br>Surgery + adjuvant RT: 4.0%<br>Surgery alone: 2.6%<br>Chemotherapy alone: 1.6%<br>Palliative, unknown: 6.7%	Two datasets from one academic US institution. No explicit statement on treatment with curative intent.	OS not defined.<br><br>Median 61 months (74% and 61% of patients had at least 3 and 5 years of follow-up, respectively, or died before 3 years and 5 years).
Bossi 2018, Italy<br><br>***External validation of Fakhry 2017 nomogram***	Consecutive patients from one institution (Milan, Italy) 2004-2016; retrospective analysis; n=184, n=174 (95%) in analysis	Mean age not reported. ≤50yrs: 11%; >50yrs: 89%; 79.9% male	Not reported.	TNM 7th and 8th eds.<br>T2-3: 51.7%,<br>T4: 48.3%<br>N0-2b (TNM7) or N0-N1 (TNM8): 50.6%<br>N2c-3 (TNM7) or N2-N3 (TNM8): 49.4%	P16 +: 69.5%<br>P16-: 30.5%<br><br>Method not reported.	a. ≤10 PYs: 33.3%, >10 PYs: 66.7%<br><br>b. Not reported<br><br>c. Zubrod/ECOG/WHO PS: PS0: 77%<br>PS1: 23%	RT: 2.3%<br>RT+CT: 90.2%<br>RT + Cetuximab: 7.5%	Single Italian institution. Patients with locally advanced T2-4 OPSCC. All treated with curative intent.	OS and PFS not defined.<br><br>Median 51 months (interquartile range, 29-65 months).
Cheng 2021, Taiwan<br><br>***Development cohort***	Patients treated June 2006 - Dec 2017 (Linkou Chang Gung Memorial Hospital (CGMH)); retrospective	Median 53 (47-60); 92% male	TNM 7th ed<br><br>I: 2%<br>II: 9%<br>III:12%<br>IVA: 61%<br>IVB: 17%<br>IV (missing	TNM 7th ed<br><br>T1: 5%, T2: 32%, T3: 21%, T4a; 30% T4b: 13%, T4 (substage missing): 0%, N0: 21%, N1: 9%, N2: 63%, N3: 7%	HPV status defined by P16 IHC: positive: 21% negative: 79%<br>Missing:0%<br><br>Method not reported.	a. PY>10: 68%, ≤10: 24%, missing: 7%<br><br>b. Yes: 72%, No: 28%<br><br>c. Not reported	Primary treatment surgery: 4%<br>Primary treatment RT: 96%<br>CT yes: 94%, no: 6%	Patients from one Taiwanese institution. Younger patients than other cohorts, and more HPV- (though large amount of missing data	OS: defined as the time from cancer diagnosis to the last follow-up or death from any cause.<br><br>Median 2.8 years (IQR 1.5–5.6)

Study (cohort)	Source of data (n, and n included in analysis)	Age (mean y (SD)), sex	Cancer stage	TNM	HPV status and measurement method	a. Smoking<br>b. Alcohol<br>c. Co-morbidities	Treatment	Inclusion criteria/ comments on cohort representative ness	Outcomes, length of follow-up
	analysis; n=268		substage): 0%					from other cohorts). Higher mortality rate compared with EV cohort I (53% vs 20%). Inclusion criteria were: (i) nonmetastatic (M0) OPSCC and absence of other concomitant malignancies, (ii) availability of baseline pre-treatment PET images covering the head and neck region, (iii) treatment with curative intent, and (iv) follow-up continued for at least 18 months or until death. Patients without identifiable tumours on PET/CT scans were excluded.	
Cheng 2021, Taiwan<br><br>***External validation 1***	Patients treated at six centres (Canada, US, Netherlands,	Median 61 (54-67); 79% male	TNM 7th ed<br><br>I: 2%<br>II: 7%	TNM 7th ed<br><br>T1: 15%, T2: 42%, T3: 25%; T4a: 14%, T4b:	HPV status defined by P16 IHC: positive: 31%<br>negative: 13%	a. Not reported<br><br>b. Not reported<br><br>c. Not reported	Primary treatment surgery: 3%	Patients from six centres (Canada, the US and the Netherlands).	OS: defined as the time from cancer diagnosis to the last follow-up or death from any cause.

Study (cohort)	Source of data (n, and n included in analysis)	Age (mean y (SD)), sex	Cancer stage	TNM	HPV status and measurement method	a. Smoking<br>b. Alcohol<br>c. Co-morbidities	Treatment	Inclusion criteria/ comments on cohort representative ness	Outcomes, length of follow-up
of Cheng 2021 models	data from TCIA database) Oct 2033—Nov 2014; retrospective analysis; n=353		III: 14%<br>IVA: 69%<br>IVB: 8%<br>IV (missing substage): 1%	2%, T4 (substage missing): 1%, N0: 14%, N1: 10%, N2: 70%, N3: 6%	Missing: 56%<br><br>Method not reported.		Primary treatment RT: 97%<br>CT yes: 72%, no: 28%	Inclusion and exclusion criteria as above.	Median 4.3 years (IQR 2.9–6.6)
	Patients treated at six centres (Canada, US, Netherlands, data from TCIA database) Oct 2033—Nov 2014; retrospective analysis; n=353; ***n=151 with known HPV status***	Median 61 (55–65); 75% male	TNM 7th ed<br><br>I: 3%<br>II: 7%<br>III: 13%<br>IVA: 70%<br>IVB: 6%<br>IV (missing substage): 2%	TNM 7th ed<br><br>T1: 17%, T2: 41%, T3: 25%, T4a: 14%, T4b: 2%, T4 (substage missing): 3%, N0: 15%, N1: 11%, N2: 70%, N3: 5%	HPV status defined by P16 IHC: positive: 70% negative: 30%<br>Missing: 0%<br><br>Method not reported.	a. Not reported<br><br>b. Not reported<br><br>c. Not reported	Primary treatment surgery: 3%<br>Primary treatment RT: 97%<br>CT yes: 67%, no: 33%	Patients from six centres (Canada, the US and the Netherlands). Inclusion and exclusion criteria as above.	OS: defined as the time from cancer diagnosis to the last follow-up or death from any cause.<br><br>Median 3.9 years (IQR 2.8–5.5)
Cheng 2021, Taiwan<br><br>***External validation 2 of Cheng 2021 models***	Patients treated Apr 2011-Mar 2019 at two hospitals in China; retrospective analysis; n=31	Median 59 (55-65); 74% male	TNM 7th ed<br><br>I: 10%<br>II: 6%<br>III: 10%<br>IVA: 61%<br>IVB: 13%<br>Stage IV (missing substage): 0%	TNM 7th ed<br><br>T1: 16%, T2: 29%, T3: 3%, T4a: 39%, T4b: 6%,<br>T4 (substage missing: 6%, N0: 32%, N1: 19%, N2: 39%, N3: 10%	HPV status defined by P16 IHC: positive: 0% negative:3% missing: 97%<br><br>Method not reported.	a. Not reported<br><br>b. Not reported<br><br>c. Not reported	Primary treatment surgery: 77%<br>Primary treatment RT: 23%<br>CT yes: 81%, no: 19%	Patients from two Chinese hospitals. Inclusion and exclusion criteria as above. Larger proportion treated with surgery including patients with advanced	OS: defined as the time from cancer diagnosis to the last follow-up or death from any cause.<br><br>Median 2.3 years (IQR 1.3–2.8)

Study (cohort)	Source of data (n, and n included in analysis)	Age (mean y (SD)), sex	Cancer stage	TNM	HPV status and measurement method	a. Smoking<br>b. Alcohol<br>c. Co-morbidities	Treatment	Inclusion criteria/ comments on cohort representativeness	Outcomes, length of follow-up
								disease who would normally be treated with CRT.	
Choi 2020, Republic of Korea<br><br>***Development cohort***	Patients from a single centre Jan 2009-Sep 2019; retrospective analysis; n=61 (NB from split sample)	Mean 60.8 (8.7); 90.2% male	TNM 8th ed<br><br>I: 32.8%<br>II: 31.1%<br>III: 21.3%<br>IVA: 13.1%<br>IVB: 1.6%	TNM 8th ed<br><br>T1:23%, T2: 54.1%, T3: 18%, T4: 4.9%, N0: 21.3%, N1: 39.3%, N2: 36.1%, N3: 3.3%	HPV (DNA) positive: 62.3%<br>Negative: 37.7%<br><br>Tumour HPV DNA detection (in situ hybridisation).	a. Smoker yes: 62.3%; No: 37.7%<br><br>b. Not reported<br><br>c. Not reported	Surgery: 70.5%, CT: 59%, RT: 65.6%	Inclusion criteria: 1) pathologically confirmed OPSCC, 2) known HPV status, and 3) available pre-treatment contrast-enhanced neck CT images. Exclusion criteria: 1) primary tumour not visible on CT, 2) beam-hardening artifacts hampering image analysis, and 3) other underlying malignancy or distant metastases at the time of OPSCC diagnosis. No explicit statement on treatment with curative intent.	OS: defined as the interval from the date of initial diagnosis to the date of death or last documented clinical visit.<br><br>Not reported.
Choi 2020, Republic of Korea<br><br>***Internal validation cohort***	Patients from a single centre Jan 2009-Sep 2019; retrospective analysis; n=25 (NB from split sample)	60.9 (8.2); 92% male	TNM 8th ed<br><br>I: 20%<br>II: 24%<br>III: 40%<br>IVA: 12%<br>IVB: 4%	TNM 8th ed<br><br>T1: 20%, T2: 32%, T3: 36%, T4: 12 %, N0: 20%, N1: 32%, N2: 44%, N3: 4%	HPV (DNA) positive: 60%<br>Negative: 40%<br><br>Tumour HPV DNA detection (in situ hybridisation).	a. Smoker yes: 48%; No: 52%<br><br>b. Not reported<br><br>c. Not reported	Surgery: 68%, CT: 80%, RT: 80%		OS: defined as the interval from the date of initial diagnosis to the date of death or last documented clinical visit.<br><br>Not reported.

Study (cohort)	Source of data (n, and n included in analysis)	Age (mean y (SD)), sex	Cancer stage	TNM	HPV status and measurement method	a. Smoking<br>b. Alcohol<br>c. Co-morbidities	Treatment	Inclusion criteria/ comments on cohort representative ness	Outcomes, length of follow-up
Choi 2020, Republic of Korea<br><br>***External validation of Choi 2020***	Patients from one dataset within the Cancer Imaging Archive (TCIA): The MAASTRO Head-Neck-Radiomics-HN1 dataset; retrospective analysis; n=78	60.2 (7.6); 78.2% male	TNM 7th ed<br><br>I: 6.4%<br>II: 9%<br>III: 12.8%<br>IVA: 62.8%<br>IVB: 9%	TNM 7th ed<br><br>T1: 16.7%, T2: 30.8%, T3: 12.8%, T4: 39.7%, N0: 25.6%, N1: 14.1%, N2: 57.7%, N3: 2.6%	HPV P16 staining. Positive: 29.5%<br>Negative: 70.5%<br><br>Tumour p16 protein expression (immunohistochemical analysis); Tumour HPV DNA detection (in situ hybridisation).	a. Not reported<br><br>b. Not reported<br><br>c. Not reported	Surgery: 3.8%, CT: Not reported, RT: 100%	Dutch patients from one dataset (MAASTRO Head-Neck-Radiomics-HN1 dataset). Inclusion criteria as above. Fewer HPV+ compared with DEV cohort. TNM7 used rather than TNM8.	OS: defined as the interval from the date of initial diagnosis to the date of death or last documented clinical visit.<br><br>Not reported.
Fakhry 2017, US<br><br>***Development cohort***	Patients from RTOG 0129 and 0522 clinical trials (2002-2005 and 2005-2009), not consecutive, n=493	Mean age NR; 27.6% ≤50 yrs, 72.4% >50 yrs; 87.4% male	All Stage III-IV as per the original RTOG studies.	TNM 7th and 8th eds<br><br>T2-3: 72.0%; T4: 28.0%<br>N0-2b (TNM7) or N0-N1 (TNM8): 66.5%;<br>N2c-3 (TNM7) or N2-N3 (TNM8): 33.5%	P16 +: 73.6%<br>P16-: 26.4%<br><br>Tumour p16 protein expression (immunohistochemical analysis)	a. ≤10 PYs: 47.3%, >10 PYs: 52.7%<br><br>b. Not reported<br><br>c. Zubrod/ECOG/WHO PS: PS0: 69.0%; PS1: 31.0%	All patients curative RT+CT.	Patients from two US trials (NRG Oncology RTOG 0129 and 0522). Eligible patients had untreated, pathologically confirmed, AJCC 5th edition (RTOG 0129) or 6th edition (RTOG 0522) stage III to IV7 head and neck squamous cell carcinoma, Zubrod performance status of 0 to 1, age ≥ 18 years, and adequate	OS: defined as time from date of randomisation to death from any cause.<br><br>PFS: defined as from date of randomization to local, regional, or distant progression or death from any cause.<br><br>Median: 5.7 years (95% CI, 5.2 to 6.0 years)

Study (cohort)	Source of data (n, and n included in analysis)	Age (mean y (SD)), sex	Cancer stage	TNM	HPV status and measurement method	a. Smoking<br>b. Alcohol<br>c. Co-morbidities	Treatment	Inclusion criteria/ comments on cohort representativeness	Outcomes, length of follow-up
								bone marrow, hepatic, and renal function.	
Fakhry 2017, US<br><br>***External validation of Fakhry 2017 nomogram***	Patients from RTOG 9003 clinical trial (1991-1997), not consecutive, n=153	Mean age NR; 25.5% ≤50 yrs, 74.5% >50 yrs; 77.8% male	All Stage III-IV as per the original RTOG studies.	TNM 7th and 8th eds<br><br>T2-3: 76.5%; T4: 23.5%<br>N0-2b (TNM7) or N0-N1 (TNM8): 70.6%<br>N2c-3 (TNM7) or N2-N3 (TNM8): 29.4%	P16 +: 41.2%<br>P16-: 58.8%<br><br>Tumour p16 protein expression (immunohistochemical analysis)	a. ≤10 PYs: 20.9%, >10 PYs: 79.1%<br><br>b. Not reported<br><br>c. Zubrod/ECOG/WHO PS: PS0: 66.0%; PS1: 34.0%	All patients curative RT+CT.	Patients from one US trial (NRG Oncology RTOG 9003). Eligible patients had untreated, pathologically confirmed, stage II to IV7 head and neck squamous cell carcinoma, Zubrod performance status of 0 to 2, and age ≥ 18 years.	OS: defined as time from date of randomization to death from any cause<br><br>PFS: defined as time from date of randomization to local, regional, or distant progression or death from any cause<br><br>Median 16.1 years (95% CI, 15.1 to 17.7 years)
Grønhøj 2018, Denmark<br><br>***Development cohort (NB high overlap with Rasmussen 2019 and Gronhøj-Larsen 2016 cohort)***	Consecutive OPSCC patients from Eastern Denmark (2000-2014); retrospective analysis; n=1313	Median [IQR] 59.81 [53.93, 66.38]; 72.1% male	TNM 8th ed<br><br>I:44.7%<br>II: 19.8%<br>III: 15.7%<br>IV: 19.8%	TNM 8th ed<br><br>T1: 21.2%, T2: 46.8%, T3: 22.5%, T4: 9.5%, N0: 21.5%, N1: 52.2%, N2: 15.7% N3: 10.6%, M1: 1.1%	P16+: 64.2%<br>P16- : 35.8%<br>HPV_DNA+: 62.3%<br>HPV_DNA- : 37.7%<br><br>Tumour p16 protein expression (immunohistochemical analysis); Tumour HPV DNA detection (PCR and hybridisation).	a. Current smoker 38.8%; Ex-smoker 40.5%; Never smoked (0 PY) 20.7%<br><br>b. Not reported<br><br>c. Not reported	RT: 53.2%, CRT: 44.6%, Surgery + RT/CT: 0.8%, Surgery: 1.5%, Unspecified curative treatment: 0%	Patients from Eastern Denmark. Treated with curative intent.	OS: defined as time from diagnosis of OPSCC to death from any cause.<br><br>PFS: defined as time from diagnosis of OPSCC to time of progression at any site or death from any cause; progression was based on a biopsy or relevant imaging.<br><br>No details on length of follow-up.

Study (cohort)	Source of data (n, and n included in analysis)	Age (mean y (SD)), sex	Cancer stage	TNM	HPV status and measurement method	a. Smoking<br>b. Alcohol<br>c. Co-morbidities	Treatment	Inclusion criteria/ comments on cohort representativeness	Outcomes, length of follow-up
Grønhøj 2018, Denmark<br><br>***External validation 1 of Grønhøj 2018***	OPSCC patients from Giessen University Hospital, Germany (2000-2009); retrospective analysis; n=344	Median [IQR] 58.89 [52.69, 64.97]; 77.0% male	TNM 8th ed<br>I:16.9%<br>II:18.3%<br>III:14.5%<br>IV:50.3%	TNM 8th ed<br><br>T1: 21.8%, T2: 28.5%, T3: 24.4%, T4: 25.3%, N0: 27.6%, N1: 13.7%; N2: 54.4% ,N3: 4.4%, M1: 9.0%	P16+:26.2%<br>P16- :73.8%<br>HPV_DNA+: 25.6%<br>HPV_DNA-: 74.4%<br><br>Tumour p16 protein expression (immunohistochemical analysis); Tumour HPV DNA detection (PCR followed by bead-based hybridisation).	a. Current smoker 77.0%; Ex-smoker 11.6%; Never smoked (0 PY) 11.3%<br><br>b. Not reported<br><br>c. Not reported	RT: 5.5%<br>CRT: 35.2%<br>Surgery + RT/CT: 40.1%<br>Surgery: 15.1%<br>Unspecified curative treatment: 4.1%	Patients from one German institution. Treated with curative intent. Fewer p16+ patients compared with other cohorts.	
Grønhøj 2018, Denmark<br><br>***External validation 2 of Grønhøj 2018***	OPSCC patients from Karolinska, Sweden (2005-2012); retrospective analysis; n=503	Median [IQR] 60.00 [53.00, 67.00]; 74.2% male	TNM 8th ed<br><br>I: 19.3%<br>II: 44.1%<br>III: 20.9%<br>IV: 15.7%	TNM 8th ed<br><br>T1: 24.7%, T2: 35.4%,<br>T3: 20.1%, T4: 19.9%, N0: 20.1%, N1: 18.7%, N2: 57.1%, N3: 4.2%, M1: 0.8%	P16+: 75.9%<br>P16- :24.1%<br>HPV_DNA+: 77.7%;<br>HPV_DNA-: 22.3%<br><br>Tumour p16 protein expression (immunohistochemical analysis); Tumour HPV DNA detection (bead-based multiplex assay).	a. Current smoker 35.4%; Ex-smoker 32.6%; Never smoked (0 PY) 32.0%<br><br>b. Not reported<br><br>c. Not reported.	RT: 58.1%<br>CRT: 40.0%<br>Surgery + RT/CT: 0%;<br>Surgery: 0%<br>Unspecified curative treatment: 2.0%	Patients from one Swedish institution. Treated with curative intent.	
Grønhøj 2018, Denmark	OPSCC patients from The Predictr consortium	Median [IQR] 56.00 [50.00,	TNM 8th ed<br><br>I: 14.3%	TNM 8th ed<br><br>T1:18.4%, T2: 39.7%, T3: 21.6%,	P16+: 65.7%<br>P16- :34.3%<br>HPV_DNA+:62.9	a. Current smoker 39.5%; Ex-smoker 33.7%; Never	RT: 10.2%<br>CRT: 33.3%<br>Surgery +	Patients from The Predictr consortium, UK.	

Study (cohort)	Source of data (n, and n included in analysis)	Age (mean y (SD)), sex	Cancer stage	TNM	HPV status and measurement method	a. Smoking<br>b. Alcohol<br>c. Co-morbidities	Treatment	Inclusion criteria/ comments on cohort representative ness	Outcomes, length of follow-up
External validation 3 of Grønhøj 2018	UK (Multi-site); retrospective analysis; n=463	63.00]; 73.4% male	II: 48.4%<br>III: 18.8%<br>IV: 18.6%	T4: 20.3%, N0: 25.1%, N1: 16.6%, N2: 54.4%, N3: 3.9%, M1: 0.6%	% HPV_DNA- : 37.1%<br><br>Tumour p16 protein expression (immunohistochemical analysis); Tumour HPV DNA detection (in situ hybridisation).	smoked (0 PY) 26.8%<br><br>b. Not reported.<br><br>c. Not reported.	RT/CT: 49.5%<br>Surgery: 7.1%<br>Unspecified curative treatment: 0%	Treated with curative intent.	
Grønhøj-Larsen 2016, Denmark<br><br>***Development cohort (NB high overlap with Grønhøj 2018 and Rasmussen 2019 cohorts)***	Consecutive, population-based, non-selected cohort of patients diagnosed 2000-2014; retrospective analysis; total cohort n=1838, final sample n=1542 (excluding those with missing data)	Median age 60 (at diagnosis);<br>---<br>72% male	TNM ed NR<br>I: 3.2%, II: 10.1%, III: 20.9%, IV: 65.8%	T1:19.9%, T2:43.9%, T3:24.2%, T4:11.9%<br>N0:20.0%, N1:18.2%, N2:53.6%, N3:8.2%,<br>M0: 97.8%, M1:2.2%	p16+: 61.2%, p16-: 38.8%; HPV DNA+: 60%, HPV DNA-: 40%<br><br>Tumour p16 protein expression (immunohistochemical analysis); Tumour HPV DNA detection (PCR).	a. 0 pk yrs: 27.2%, 1-10 pk yrs: 7.5%, 11-20 pk yrs: 9.7% ,>20 pk yrs: 55.2%. Median n pk yrs: 27 (mean 30).<br><br>b. Not reported.<br><br>c. Performance score: 0: 66.7%, 1: 24.5%, 2: 6.1%, 3: 2.3%, 4: 0.3%	RT: 51.2% , CRT: 39.9%, palliative: 6.5%, no treatment: 2.4%.<br><br>87.7% were treated with curative regimes.	Unselected, population-based Danish cohort of patients. 88% treated with curative intent.	OS: defined as time from diagnosis of OPSCC to death due to any cause.<br><br>Time to progression (TTP): defined as defined as the time from diagnosis of OPSCC to time of progression at any site<br><br>Survival after progression (SAP): defined as defined as the time from progression to death due to any cause.<br><br>Median follow-up 4.0 yrs (range: 0.8–15.8 yrs)

Study (cohort)	Source of data (n, and n included in analysis)	Age (mean y (SD)), sex	Cancer stage	TNM	HPV status and measurement method	a. Smoking<br>b. Alcohol<br>c. Co-morbidities	Treatment	Inclusion criteria/ comments on cohort representative ness	Outcomes, length of follow-up
Mentel 2021, UK<br><br>***External validation 3 of Grønhøj 2018***	OPSCC patients from Greater Glasgow and Clyde ENT and OMFS departments, UK; retrospective analysis; n=317	Median [IQR] 59 [53-65]; 76.7% male	TNM 8th ed<br><br>I:29.3%<br>II: 20.1%<br>III: 28.1%<br>IV: 21.8%	TNM 8th ed<br><br>T1: 24.9%, T2: 34.7%, T3: 11.7%, T4: 28.7%, N0: 29.3%, N1: 44.2%, N2: 24.6%, N3: 1.9%, M1: 0%	P16+: 61.8%<br>P16- :38.2%<br>HPV_DNA+: 54.3%; HPV DNA- : 45.7%<br><br>Tumour p16 protein expression (immunohistochemical analysis); Tumour HPV DNA detection (PCR).	a. Current smoker 51.7%; Ex-smoker 24.9%; Never smoked (0 PY) 23.3%<br><br>b. Not reported<br><br>c. Not reported.	RT: 19.6%, CRT: 35.6%, Surgery + RT/CT: 32.2%, Surgery: 11.0%, CT only: 1.6%	Scottish (Glaswegian) patients treated with curative intent.	OS: not defined.<br><br>PFS: not defined.<br><br>No details on length of follow-up.
Mes 2020, The Netherlands<br><br>***Development cohort***	HPV- OPSCC patients 2008 to 2012 (Amsterdam University Medical Centre); retrospective analysis; n=89	Median (mean absolute deviation) 60 (7.4); 55.1% male	TNM 7th ed<br>I: 4.5%<br>II: 19.1%<br>III: 16.9%<br>IV: 59.6%	TNM 7th ed<br><br>T1: 7.9%,,T2: 39.3%, T3: 18.0%, T4: 34.8%, N0: 44.9%, N1: 15.7%, N2: 39.3%, N3: 0%	100% HPV-<br><br>Tumour p16 protein expression (immunohistochemical analysis); Tumour HPV DNA detection (PCR).	a. Current: 60.7%, former: 29.2%, never: 10.1%, unknown: 0%.<br><br>b. Current: 74.2%, former: 13.5%, never: 12.4%, unknown: 0%.<br><br>c. ACE-27 score 0: 29.2%, 1: 37.1%, 2: 30.3%, 3: 3.4%, unknown: 0%	All patients were treated with curative intent (no further details).	Patients from one Dutch centre. HPV-positive tumours were excluded.	OS: defined as time from date of incidence to death from any cause.<br><br>RFS: defined as time from date of incidence to development of locoregional recurrence, distant metastasis, or second primary HNSCC.<br><br>Median (alive patients) yrs (mean absolute deviation): 5.9 (1.7)

Study (cohort)	Source of data (n, and n included in analysis)	Age (mean y (SD)), sex	Cancer stage	TNM	HPV status and measurement method	a. Smoking<br>b. Alcohol<br>c. Co-morbidities	Treatment	Inclusion criteria/ comments on cohort representative ness	Outcomes, length of follow-up
Mes 2020, The Netherlands<br><br>***External validation of Mes 2020***	HPV- OPSCC patients 2010 to 2013 (University Medical Centre Utrecht); retrospective analysis; n=56	Median (mean absolute deviation) 64 (11.9); 62.5% male	TNM 7th ed<br>I: 7.1%<br>II: 10.7%<br>III: 12.5%<br>IV: 69.6%	TNM 7th ed<br><br>T1: 10.7%, T2: 30.4%, T3: 23.2%, T4: 35.7%, N0: 32.1%, N1: 12.5%, N2: 53.6%, N3: 1.8%	100% HPV-<br><br>Tumour p16 protein expression (immunohistochemical analysis); Tumour HPV DNA detection (PCR).	a. Current: 60.7%, former: 23.2%, never: 10.7%, unknown: 5.4%.<br><br>b. Current: 71.4%, former: 17.9%, never: 5.4%, unknown:5.4%.<br><br>c. ACE-27 score 0: 30.4%, 1: 48.2%, 2: 12.5%, 3: 1.8%, unknown: 7.1%.	All patients were treated with curative intent (no further details).	Patients from one Dutch centre. HPV-positive tumours were excluded.	OS: defined as time from date of incidence to death from any cause.<br>RFS: defined as time from date of incidence to development of locoregional recurrence, distant metastasis, or second primary HNSCC.<br><br>Median (alive patients) yrs (mean absolute deviation): 5.0 (0.5)
Nelson 2022, US<br><br>***External validation of Fakhry 2017 nomogram***	National registry data (Veterans Health Administration - Corporate Data Warehouse (VHA-CDW), USA, 2008-2016; retrospective analysis; full cohort n=4007; n=658 with complete data.	Complete data cohort:<br>≤50 yrs: 3.0%; 51-60 yrs: 26.0%; 61-70 yrs: 51.8%; >70 yrs: 19.1%; 99% male	TNM 7th ed stage III-IV	Complete data cohort: T1: 16.4%; T2: 39.8%<br>T3: 23.4%; T4A: 17.9%<br>T4B: 2.4%<br>N0: 6.8%; N1:14.9%<br>N2: 5.8%; N2A: 7.8%<br>N2B: 40.1%; N2C: 21.6%; N3: 3.0%<br>M0: 100%	Complete data cohort:<br>P16+: 77.5%<br>P16-: 22.5%<br><br>Method not reported.	a. Complete data cohort:<br>Smoking yes: 83.4%; none: 16.6%<br><br>b. Not reported<br><br>c. Zubrod/ECOG/WHO PS: PS0: 47.6%; PS1:45.6%; PS≥2: 6.8%. Charlson Comorbidity Index 0: 46.4%; 1: 24.2%; ≥2: 29.5%	All patients had definitive RT.	US veterans treated with definitive radiation. Patients with AJCC 7th edition stage I or II disease, metastases at presentation, ECOG performance status ≥3, or who received postoperative radiotherapy were excluded. *"The VA data set, which is almost entirely comprised of*	OS: defined as the time from diagnosis until death from any cause or censoring.<br><br>PFS: defined as the time from diagnosis until cancer progression, death from any cause, or censoring.<br><br>Median follow-up time 3.20 years for all cases and 1.95 years for complete cases.

Study (cohort)	Source of data (n, and n included in analysis)	Age (mean y (SD)), sex	Cancer stage	TNM	HPV status and measurement method	a. Smoking<br>b. Alcohol<br>c. Co-morbidities	Treatment	Inclusion criteria/ comments on cohort representative ness	Outcomes, length of follow-up
								men with relatively equal access to health care, is itself a skewed representation of the general population."	
Rasmussen 2019, Denmark<br><br>**Developme nt cohort**<br><br>***NB high overlap with Grønhøj 2018 and Grønhøj-Larsen 2016 cohorts***	Consecutive patients diagnosed with OPSCC and treated with curative radiotherapy +/-<br>cisplatin in eastern Denmark from 2000 to 2014; retrospective analysis; n=1243 (all included in analysis)	Mean 60.2 (SD 9.3) ;72.6% male	TNM 8th ed<br><br>No details	TNM 8th ed<br><br>T1: 21.7%; T2: 46.4%;<br>T3: 22.8%; T4: 9.1%;<br>N0: 21.2%; N1: 53.0%; N2: 17.1%; N3: 8.7%	P16+: 66.1%, P16- :33.9%.<br><br>HPV_DNA+: 63.4%;<br>HPV_DNA- :36.6 %<br><br>Tumour p16 protein expression (immunohistoche mical analysis); Tumour HPV DNA detection (PCR).	a. Non-smoker (0 PY) 22.5% ; 0-10 PY 8.0%; 10-20 PY 9.6%; >20 PY 59.9%.<br><br>b. Not reported.<br><br>c. COG/WHO PS 0: 237 (69.9%); PS1-3: 102 (30.1%)	RT+/- CT	Patients from Eastern Denmark. Treated with curative intent. Patients with M1 disease excluded.	T-site recurrence: defined as T-site recurrence or simultaneous T- and N-site recurrence.<br>N-site recurrence: defined as N-site recurrence.<br><br>M-site recurrence: defined as M-site recurrence, T- and/or N-site and M-site recurrence.<br><br>Death with no evidence of disease (NED): defined as death with no evidence of prior recurrence.<br><br>Median 7.2 years
Rasmussen 2019, Denmark<br><br>**External validation of**	Patients with OPSCC treated at the Department of Oto-Rhino-Laryngology, Head and	Mean 59.4 (SD 9.4); 78.2% male	TNM 8th ed<br><br>No details	TNM 8th ed<br><br>T1: 22.4%; T2: 30.1%<br>T3: 23.0%; T4: 24.5%	P16+: 25.1%, P16- : 74.9%.<br><br>HPV_DNA+: 24.5%,<br>HPV_DNA- : 75.5%	a. Non-smoker (0 PY) 10.9%; 0-10 PY 4.7%; 10-20 PY 7.4%; >20 PY 77.0%.<br><br>b. Not reported.	Surgery +/- RT OR CRT	Patients from one German institution treated with curative intent (treated more heterogeneously	T-site recurrence: defined as T-site recurrence or simultaneous T- and N-site recurrence.

Study (cohort)	Source of data (n, and n included in analysis)	Age (mean y (SD)), sex	Cancer stage	TNM	HPV status and measurement method	a. Smoking<br>b. Alcohol<br>c. Co-morbidities	Treatment	Inclusion criteria/ comments on cohort representativeness	Outcomes, length of follow-up
**Rasmussen 2019 models**	Neck Surgery of the University of Giessen, Germany from 2000 to 2009; retrospective analysis; n=339 (all included in analysis)			N0: 28.9%; N1: 24.2%; N2: 30.7%; N3: 16.2%	Tumour p16 protein expression (immunohistochemical analysis); Tumour HPV DNA detection (PCR).	c. ECOG/WHO PS 0 237 (69.9%); PS1-3 102 (30.1%)		compared with development cohort, also differences in p16 status).	N-site recurrence: defined as N-site recurrence.<br><br>M-site recurrence: defined as M-site recurrence, T- and/or N-site and M-site recurrence.<br><br>Death with no evidence of disease (NED): defined as death with no evidence of prior recurrence.<br><br>Median 9.2 years
Rios-Velaquez 2014, The Netherlands<br><br>***Development cohort***	Consecutively treated patients (Maastro Clinic) Jan 2000-Oct 2011; retrospective analysis; n=168	Median 59 (range 43-83); 74.4% male	TNM vr NR<br><br>I–IVb (no further details)	TNM vr NR<br><br>T1: 14.9%, T2: 27.4%, T3: 22.6%, T4: 35.1%, N0: 34.5%, N1: 17.3%, N2: 44.1%, N3: 3.6%, Nx: 0.6%	P16+: 34.5%<br>P16- : 64.3%<br>P16 unknown: 1.25%<br><br>HPV_DNA+: 30.4%<br>HPV_DNA-: 69.6%<br><br>Tumour p16 protein expression (immunohistochemical analysis); Tumour HPV 16 DNA detection (PCR).	a. Median 30PY (range 0-100)<br><br>b. Median 134 units/years (range 0-660)<br><br>c. ACE 27<br>None: 33.3%, Mild: 41.1%<br>Moderate: 19%, Severe: 6.5%	RT only: 67.9%<br>CRT: 32.1%	Single Dutch institution. Patients treated with curative intent.	OS: defined as the time from starting radiotherapy to death from any cause.<br><br>PFS: defined as the time from starting radiotherapy to time of first documented recurrence at any site (locoregional or metastasis) or death from any cause.<br><br>Median 26 months (range 2.5–127.2) overall.<br><br>Median 37.5 months (range 6.4–127.2) for

Study (cohort)	Source of data (n, and n included in analysis)	Age (mean y (SD)), sex	Cancer stage	TNM	HPV status and measurement method	a. Smoking<br>b. Alcohol<br>c. Co-morbidities	Treatment	Inclusion criteria/ comments on cohort representativeness	Outcomes, length of follow-up
									patients alive at last follow-up.
Rios-Velaquez 2014, The Netherlands<br><br>***External validation of Rios-Velaquez 2014 nomogram***	Consecutively treated patients (VU University Medical Center) Jan 2000-Dec 2006; retrospective analysis; n=189	Median 60 (range 43–93); 64.6% male	TNM vr NR<br><br>I–IVb (no further details)	TNM vr NR<br><br>T1: 13.8%, T2: 28%, T3: 33.3%, T4: 24.9%, N0: 43.9%, N1: 12.2%, N2: 41.8%, N3: 2.1%, Nx: 0%	P16+: 16.9%<br>P16- : 82% P16 unknown: 1.1%<br><br>HPV DNA+: 18%<br>HPV DNA-: 82%<br><br>Tumour p16 protein expression (immunohistochemical analysis); Tumour HPV 16 DNA detection (PCR).	a. Median 32 PY (range 0-100)<br><br>b. Median 170 units/years (range 0-350)<br><br>c. ACE 27<br>None: 35.4%, Mild: 29.1%, Moderate: 29.6%, Severe: 5.8%	RT only: 60.8%<br>CRT: 39.2%	Single Dutch institution. Patients treated with curative intent.	OS: defined as the time from starting radiotherapy to death from any cause.<br><br>PFS: defined as the time from starting radiotherapy to time of first documented recurrence at any site (locoregional or metastasis) or death from any cause.<br><br>Length of follow-up not reported.
Ward 2014, UK<br><br>***Development cohort***	Consecutively treated patients at University Hospital Southampton 2000– 010); retrospective analysis; n=316; n=183 (58%) for analysis	Mean 58.2 (SD 11.2); 73% male<br><br>*NB NR separately for cohorts*	TNM vr NR<br><br>I/II: 19.3%<br>III/IV: 79.9%<br>Unknown: 0.7%<br><br>*NB NR separately for cohorts*	TNM vr NR<br><br>T1/2: 68.2% T3/4: 29.9%; not known: 1.8%<br><br>N0-N2a: 51.8%<br>N2b-N3: 47.4%<br>Not known: 0.7%<br><br>No nodal metastases:24.8%<br>Nodal metastases: 74.5%; not known: 0.7%	HPV+: 54.4%<br>HPV-: 44.2%<br>Unknown: 1.5%<br><br>*NB NR separately for cohorts*<br><br>Tumour p16 protein expression (immunohistochemical analysis); Tumour HPV DNA detection (in situ hybridisation).	a. Non-/ex-smoker: 39.1% Current <10 pack-years: 6.6%; Current>10 pack years: 41.6%; Not known: 12.8%<br><br>b. Non/ex-drinker: 12.4%<br>Current drinker: 60.9%<br>Not known: 26.6%<br><br>c. No details on co-morbidity	Surgery + RT: 42.7%; RT: 21.5%; CRT: 35.8%<br><br>*NB NR separately for cohorts*	Patients from one UK institution. Patients were excluded from further analysis if they had follow-up of <6 months, had received either no treatment or treatment without curative intent, or had an unknown cause of death.	3-year DDS: defined as death from OPSCC measured from the date of diagnosis until date of death from OPSCC or date last seen alive<br><br>Median (range) 58.0 (8–137) months<br><br>*NB NR separately for cohorts*

Study (cohort)	Source of data (n, and n included in analysis)	Age (mean y (SD)), sex	Cancer stage	TNM	HPV status and measurement method	a. Smoking<br>b. Alcohol<br>c. Co-morbidities	Treatment	Inclusion criteria/ comments on cohort representative ness	Outcomes, length of follow-up
Ward 2014, UK<br><br>***External validation of Ward 2014 model***	Consecutively treated patients at Poole NHS Foundation Trust and Bart's and the London NHS Trust 2000–2006; retrospective analysis; n=126; n=101 (80%) for analysis.			*NB NR separately for cohorts*		*NB NR separately for cohorts*		Patients from two UK institutions. Patients were excluded from further analysis if they had follow-up of <6 months, had received either no treatment or treatment without curative intent, or had an unknown cause of death.	
Ma 2023, The Netherlands<br><br>***Development cohort***	TCIA (The Cancer Imaging Archive) OPC-Radiomics set collected from Princess Margaret Cancer Centre, Toronto, Canada; retrospective analysis; n=524 (n=367 for development, n=157 for internal validation)	61 (10)<br><br>82% male	Not reported	TNM 7th ed<br><br>T1: 12.5%<br>T2: 32.4%<br>T3: 33.8%<br>T4: 21.2%<br>N0: 15.0%<br>N1: 10.6%<br>N2: 67.3%<br>N3: 7.1%	HPV+: 55.9%<br>HPV-: 24.3%<br>Unknown: 19.9%<br><br>Tumour p16 protein expression (immunohistochemical analysis).	a. Current smoker 32.4%, non-smoker 29.4%, ex-smoker 38.1%<br><br>b. Not reported<br><br>c. WHO PS 0: 61.9%, 1: 26.7%, 2: 9.0%, 3: 1.4%, 4: 0.3%	RT +/- CT (proportions not reported)	No inclusion or exclusion criteria reported and no details on whether treatment was with curative intent.	2-year LC or RC: defined as recurrent or residual tumour within or around the primary site or the regional nodes within 2 years after radiotherapy<br><br>2-year LRC: includes events of LC and RC.<br><br>2-year DMFS: distant metastasis as event<br><br>2-year DSS: defined as death related to the tumour
Ma 2023, The Netherlands<br><br>***Internal validation cohort***		61 (10)<br><br>77.7% male	Not reported	TNM 7th ed<br><br>T1: 11.5%<br>T2: 32.5%<br>T3: 32.5%<br>T4: 23.6%<br>N0: 22.3%<br>N1: 8.3%<br>N2: 65.0%	HPV+: 63.7%<br>HPV-: 22.9%<br>Unknown: 13.4%<br><br>Tumour p16 protein expression (immunohistochemical analysis).	a. Current smoker 31.8%, non-smoker 24.2%, ex-smoker 43.9%<br><br>b. Not reported	RT +/- CT (proportions not reported)	No inclusion or exclusion criteria reported and no details on whether treatment was with curative intent.	

Study (cohort)	Source of data (n, and n included in analysis)	Age (mean y (SD)), sex	Cancer stage	TNM	HPV status and measurement method	a. Smoking<br>b. Alcohol<br>c. Co-morbidities	Treatment	Inclusion criteria/ comments on cohort representative ness	Outcomes, length of follow-up
				N3: 4.5%		c. WHO PS 0: 66.2%, 1: 23.6%, 2: 7.0%, 3: 2.5%, 4: 0%			2-year OS: defined as death by any cause<br><br>2-year DFS: included all events above.<br><br>Mean length of follow-up not reported. Patients with follow up < 2 years and no events were censored.
Ma 2023, The Netherlands<br><br>***External validation of Ma 2023***	OPSCC patients collected from the University Medical Centre Groningen, the Netherlands (UMCG) 2007-2020; retrospective analysis; n=396	61 (9)<br><br>67.9% male	Not reported	TNM 7th ed<br><br>T1: 15.7%<br>T2: 25.3%<br>T3: 13.9%<br>T4: 45.2%<br>N0: 17.9%<br>N1: 11.6%<br>N2: 66.4%<br>N3: 3.8%	HPV+: 40.1%<br>HPV-: 49.2%<br>Unknown: 10.6%<br><br>Tumour p16 protein expression (immunohistochemical analysis).	a. Current smoker 49.2%, non-smoker 12.1%, ex-smoker 38.4%<br><br>b. Not reported<br><br>c. WHO PS 0: 70%, 1: 24%, 2: 5.3%, 3: 0.8%, 4: 0%	RT +/- CT (proportions not reported)	Patients receiving definitive radiotherapy with or without chemotherapy. Excluded patients that (1) underwent previous neck dissection (2) had multi primary tumours and (3) were without contrast-enhanced planning CT scans	
