# Supplementary material for: Systematic review of prognostic models for predicting recurrence and survival in patients with treated oropharyngeal cancer
Source: BMJ Open. 2024 Dec 5;14(12):e090393. doi: 10.1136/bmjopen-2024-090393 (PMC11624838; doi:10.1136/bmjopen-2024-090393)
Supplement: online supplemental file 9 [file bmjopen-14-12-s009.pdf]

Supplemental Material 9 Variables included in individualised prediction models

	HPV status		***TNM related variables***				***Other variables***															
	HPV status	***p16 status***	***T-stage***	***N-stage***	***M-stage***	***Overall stage***	***Smoking***	***Co-morbidity (ACE)***	***Age***	***Sex***	***Anaemia/haemoglobin***	***Performance status***	***Education***	***Age x pack years interaction***	***Marital status***	***Weight loss***	***P16 x Zubrod interaction***	***Treatment***	***CT-based Imaging features***	***Radiomics features/score***	***TIL level***	***MTV***
Individualised prediction models																						
Fakhry 2017 OS model		✓	✓	✓			✓		✓		✓	✓	✓	✓								
Fakhry 2017 PFS model		✓	✓	✓			✓		✓			✓	✓		✓	✓	✓					
Grønhøj-Larsen 2016 OS model	*Combined*		✓	✓			✓		✓									✓				
Grønhøj 2018 OS model	*Combined*		✓	✓	✓	✓		✓	✓	✓												
Grønhøj 2018 PFS model	*Combined*		✓	✓	✓			✓	✓	✓												
Rios Velazquez 2014	✓		✓	✓			✓	✓		✓	✓											
Beesley 2021 clinical model		✓	✓	✓			✓	✓	✓	✓	✓	✓										
Cheng 2021 clinical + radiomics model		✓	✓	✓		✓			✓	✓										✓		
Cheng 2021 clinical model		✓	✓	✓		✓			✓	✓												
Cheng 2021 clinical + radiomics (no HPV)			✓	✓		✓			✓	✓										✓		
Cheng 2021 clinical (no HPV)			✓	✓		✓			✓	✓												
Cheng 2021 clinical + MTV		✓	✓	✓		✓			✓	✓												✓
Cheng 2021 clinical + MTV (no HPV)			✓	✓		✓			✓	✓												✓
Choi 2020	✓		✓	✓																✓		
Mes 2020 clinical model				✓					✓	✓												
Mes 2020 clinical + radiomics model				✓					✓	✓										✓		

	HPV status		***TNM related variables***				***Other variables***															
	HPV status	***p16 status***	***T-stage***	***N-stage***	***M-stage***	***Overall stage***	***Smoking***	***Co-morbidity (ACE)***	***Age***	***Sex***	***Anaemia/haemoglobin***	***Performance status***	***Education***	***Age x pack years interaction***	***Marital status***	***Weight loss***	***P16 x Zubrod interaction***	***Treatment***	***CT-based Imaging features***	***Radiomics features/score***	***TIL level***	***MTV***
Rasmussen 2019 HPV/p-16 model	*Combined*		✓	✓				✓	✓	✓		✓										
Rasmussen 2019 p-16 model		✓	✓	✓				✓	✓	✓		✓										
Ward 2014			✓				✓														✓	
Ma 2023 clinical model		✓	✓	✓			✓		✓	✓		✓										
Ma 2023 SSL model		✓	✓	✓			✓		✓	✓		✓							✓			
Ma 2023 MLL1		✓	✓	✓			✓		✓	✓		✓							✓			
Ma 2023 MLL2		✓	✓	✓			✓		✓	✓		✓							✓			
Ma 2023 MLL + oversampling		✓	✓	✓			✓		✓	✓		✓							✓			
Ma 2023 MLL + radiomics		✓	✓	✓			✓		✓	✓		✓								✓		

OS-overall survival, PFS: progression free survival; MTV=metabolic tumour volume; TIL=tumour infiltrating lymphocytes; SLL=single-label learning based model; MLL=multi-label learning based model (MLL1 and MLL2 differ in the ß values set in the loss function).
