# Supplementary material for: Systematic review of prognostic models for predicting recurrence and survival in patients with treated oropharyngeal cancer
Source: BMJ Open. 2024 Dec 5;14(12):e090393. doi: 10.1136/bmjopen-2024-090393 (PMC11624838; doi:10.1136/bmjopen-2024-090393)
Supplement: online supplemental file 10 [file bmjopen-14-12-s010.pdf]

Supplementary material 10 Risk of bias summary by PROBAST domains

Study/model	Participants	Predictors	Outcome	Analysis	OVERALL
**Fakhry 2017 OS model DEV**	LOW	LOW	LOW	HIGH	HIGH
Fakhry 2017 EV	LOW	LOW	LOW	HIGH	HIGH
Bossi 2018 EV	LOW	LOW	HIGH	HIGH	HIGH
Nelson 2022 EV	LOW	HIGH	HIGH	HIGH	HIGH
Beesley 2019 EV	LOW	LOW	HIGH	HIGH	HIGH
Beesley 2021 EV	NEI	LOW	LOW	HIGH	HIGH
**Fakhry 2017 PFS model DEV**	LOW	LOW	NEI	HIGH	HIGH
Fakhry 2017 EV	LOW	LOW	NEI	HIGH	HIGH
Bossi 2018 EV	LOW	LOW	HIGH	HIGH	HIGH
Nelson 2022 EV	LOW	HIGH	HIGH	HIGH	HIGH
**Beesley 2021 DEV**	NEI	LOW	NEI	HIGH	HIGH
Beesley 2021 EV	NEI	LOW	NEI	NEI	NEI
**Rios-Velazquez 2014 DEV**	LOW	LOW	HIGH	HIGH	HIGH
Rios-Velazquez 2014 EV	LOW	LOW	NEI	HIGH	HIGH
Beesley 2019 EV	LOW	LOW	HIGH	HIGH	HIGH
**Gronhoj 2018 DEV OS model**	LOW	LOW	NEI	HIGH	HIGH
Gronhoj 2018 EV1	NEI	LOW	NEI	HIGH	HIGH
Gronhoj 2018 EV2	NEI	LOW	NEI	HIGH	HIGH
Gronhoj 2018 EV3	NEI	LOW	NEI	HIGH	HIGH
Mentel 2021 EV	NEI	LOW	HIGH	HIGH	HIGH
**Gronhoj 2018 DEV PFS model**	LOW	LOW	NEI	HIGH	HIGH
Gronhoj 2018 EV1	NEI	LOW	NEI	HIGH	HIGH
Gronhoj 2018 EV2	NEI	LOW	NEI	HIGH	HIGH
Gronhoj 2018 EV3	NEI	LOW	NEI	HIGH	HIGH
Mentel 2021 EV	NEI	LOW	HIGH	HIGH	HIGH
**Gronhoj-Larsen 2016 DEV**	LOW	LOW	LOW	HIGH	HIGH
Beesley 2019 EV	LOW	LOW	HIGH	HIGH	HIGH
Beesley 2021 EV	NEI	LOW	NEI	HIGH	HIGH
**Cheng 2021 DEV Integrated model**	NEI	NEI	NEI	HIGH	HIGH
Cheng 2021 EV1	NEI	NEI	NEI	HIGH	HIGH
Cheng 2021 EV2	NEI	NEI	NEI	HIGH	HIGH
**Cheng 2021 DEV Integrated model NO HPV**	NEI	NEI	NEI	HIGH	HIGH
Cheng 2021 EV	NEI	NEI	NEI	HIGH	HIGH
**Cheng 2021 DEV Clinical model**	NEI	NEI	NEI	HIGH	HIGH
Cheng 2021 EV	NEI	NEI	NEI	HIGH	HIGH
**Cheng 2021 DEV Clinical model NO HPV**	NEI	NEI	NEI	HIGH	HIGH
Cheng 2021 EV1	NEI	NEI	NEI	HIGH	HIGH
Cheng 2021 EV2	NEI	NEI	NEI	HIGH	HIGH
**Cheng 2021 DEV Clinical model + MTV**	NEI	NEI	NEI	HIGH	HIGH
Cheng 2021 EV	NEI	NEI	NEI	HIGH	HIGH

Study/model	Participants	Predictors	Outcome	Analysis	OVERALL
**Cheng 2021 DEV Clinical model + MTV NO HPV**	NEI	NEI	NEI	HIGH	HIGH
Cheng 2021 EV	NEI	NEI	NEI	HIGH	HIGH
**Choi 2020 DEV**	NEI	HIGH	NEI	HIGH	HIGH
Choi 2020 EV	NEI	HIGH	NEI	HIGH	HIGH
**Rasmussen 2019 DEV p-16 model**	LOW	LOW	NEI	HIGH	HIGH
Rasmussen 2019 EV (for TNM site recurrence)	LOW	LOW	NEI	HIGH	HIGH
Rasmussen 2019 EV (for DNED)	LOW	LOW	NEI	HIGH	HIGH
**Rasmussen 2019 DEV HPV/p-16 model**	LOW	LOW	NEI	HIGH	HIGH
Rasmussen 2019 EV (for TNM site recurrence)	LOW	LOW	NEI	HIGH	HIGH
Rasmussen 2019 EV (for DNED)	LOW	LOW	NEI	HIGH	HIGH
**Ward 2014 DEV**	LOW	LOW	NEI	HIGH	HIGH
Ward 2014 EV	LOW	LOW	NEI	HIGH	HIGH
**Mes 2020 DEV Clinical model**	NEI	HIGH	NEI	HIGH	HIGH
Mes 2020 EV	NEI	HIGH	NEI	HIGH	HIGH
**Mes DEV Clinical + radiomics model**	NEI	HIGH	NEI	HIGH	HIGH
Mes EV	NEI	HIGH	NEI	HIGH	HIGH
**Ma 2023 DEV MLL1**	NEI	LOW	NEI	HIGH	HIGH
Ma 2023 EV	LOW	LOW	NEI	HIGH	HIGH
**Ma 2023 DEV MLL2**	NEI	LOW	NEI	HIGH	HIGH
Ma 2023 EV	LOW	LOW	NEI	HIGH	HIGH
**Ma 2023 DEV MLL + oversampling**	NEI	LOW	NEI	HIGH	HIGH
Ma 2023 EV	LOW	LOW	NEI	HIGH	HIGH
**Ma 2023 DEV MLL + radiomics**	NEI	LOW	NEI	HIGH	HIGH
Ma 2023 EV	LOW	LOW	NEI	HIGH	HIGH
**Ma 2023 DEV SLL**	NEI	LOW	NEI	HIGH	HIGH
Ma 2023 EV	LOW	LOW	NEI	HIGH	HIGH
**Ma 20923 DEV Clinical model**	NEI	LOW	NEI	HIGH	HIGH
Ma 2023 EV	LOW	LOW	NEI	HIGH	HIGH

DEV=development; EV=external evaluation; OS=overall survival; PFS=progression free survival; DNED=death with no evidence of disease; MTV=metabolic tumour volume; SLL=single-label learning based model; MLL=multi-label learning based model (MLL1 and MLL2 differ in the ß values set in the loss function).
