# Supplementary material for: Systematic review of prognostic models for predicting recurrence and survival in patients with treated oropharyngeal cancer
Source: BMJ Open. 2024 Dec 5;14(12):e090393. doi: 10.1136/bmjopen-2024-090393 (PMC11624838; doi:10.1136/bmjopen-2024-090393)
Supplement: Supplementary file 11 [file bmjopen-2024-090393.reviewer_comments.pdf]

PEER REVIEW HISTORY

BMJ Open publishes all reviews undertaken for accepted manuscripts. Reviewers are asked to complete a checklist review form and are provided with free text boxes to elaborate on their assessment. These free text comments are reproduced below.

ARTICLE DETAILS

Title (Provisional)

A systematic review of prognostic models for predicting recurrence and survival in patients with treated oropharyngeal cancer

Authors

Dretzke, Janine; Abou-Foul, Ahmad; Albon, Esther; Hillier, Bethany; Scandrett, Katie; Price, Malcolm; Moore, David; Mehanna, H; Nankivell, Paul

---

VERSION 1 - REVIEW

**Reviewer**	**1**
**Name**	**Cao, Yubin**
**Affiliation**	**Sichuan University West China Hospital of Stomatology**
**Date**	**15-Jul-2024**
**COI**	**No**

I would like to raise some concerns for this overall good paper.

Abstract

1. "secondary care" may be somewhat confusing.

2. The study protocol (CRD42021248762) stated that this review will focus on head and neck cancer. However, the scope is narrower currently.

3. Too little information about the PROBAST assessment in the abstract.

4. How many is "most"?

5. The abstract almost provided no useful information for the readers.

Introduction

1. not seventh now.

2. "include" is not adequate for the study aim.

Methods

1. The difference between protocol and review is not explained with reasons.

2. eligibility criterias should be an independent part from the study selection process.

3. Many reviewers participate in the data extraction and assessment. Are they consistent?

4. Figure 3 and 4 are too small to be seen.

5. The figure title could not reflect the contents of figures acurately.

Disucussion

1. The discussion may need some re-structuring. Readers could not grasp the logic of discussion repidly.

2. Limitations and strength should be stated.

Reviewer **2**

Name **CHOI, Siu-Wai**

Affiliation **The University of Hong Kong**

Date **02-Aug-2024**

COI **Nonw**

This review is well conducted and the choice of study inclusion well documented with more than one reviewer and a starring committee

Inclusion and exclusion criteria well defined and reasonable

Detailed description of each included study is given, and a good summary is made of which variables should be included in models for better predictive abilities.

It is important to point out that multicollinearity was poorly addressed in the studies, which the current reviewer also found to be true. Many standard statistical pitfalls are not addressed.

I want to thank the authors for this updated review

Reviewer **3**

Name **Harrison, Hannah**

Affiliation **University of Cambridge, Department of Public Health and Primary Care**

Date **05-Aug-2024**

COI **I have published in this field previously - including articles that could have been (but aren't) cited in this paper.**

I enjoyed reading this paper which gives a comprehensive overview of prognostic models for oropharyngeal cancer and highlights some important issues in this area. I liked to see the

specific challenges around HPV as a key risk factor being discussed. While I am keen to recommend this paper for publication there are a couple of issues I would need to see addressed first.

1. Improved clarity on the “subject” being identified by this review. Upon careful reading, it is clear that the item/studies identified are “external validations” rather than the models themselves. However, this was not always clear to me and there are some areas in which I am not sure if I would be able to reproduce the authors approach.

• How study selection was managed. It seems to me that there are three types of study that could be discussed in the results section: model development studies, studies including both development and external validation, and external validation studies. It would be useful to have the number of each type identified stated explicitly early in the results. It would be useful to understand what happened to the model development studies that would have been excluded for not mentioning validation in the abstract, but were later reintroduced (?) if the model had external validations in other studies.

• While the forest plots clearly lay out which studies are associated with which model, it would have be useful to reference this in the text (e.g. for model X, developed by smith et al., for which 3 external validations have been carried out).

• It was unclear to me how the RoB assessment was done. It was unclear if this was done separately for each model? Or for each external validation. As the external validations are the key “subject” here – I rather expected the reporting around bias to focus on this (and not on the development of the models). A reporting by domain of the results of the RoB assessment for each study evaluated should be included, and I wasn’t able to find it. I suspect a reader who had not previously used PROBAST would have struggled to understand the distinction between the RoB and applicability assessments.

• It was not obvious to me if any of the studies identified had validated more than one model – if there were a few studies that did this, then a network meta-analysis approach may be suitable (see https://doi.org/10.1111/bju.15673)

2. A lack of specifics in the results section, in some places the reporting was vague.

• When stating that models/studies “typically” or “less often” used a certain type of cohort or risk factor, I would encourage the authors to specify exact numbers. This will require a clear distinction to be made throughout (as discussed about) between the number of models and the number of studies.

• In several places I notes statements such as “this cohort had a higher than expected number of smokers” (what was expected?) or “this cohort was deemed representative” (why?) or “event numbers were considered small” (what do you consider small? <100?) – I would advise a thorough revision of this section to avoid any ambiguity.

3. Comment on the pragmatic approach to screening. I appreciate that it is challenging to screen large numbers of papers, especially at full text stage and that the resources of

different teams may vary. However, based on carrying out similar reviews of prognostic models (for different cancers) I actually thought the number of identified studies (and hence the screening burden) was relatively low. I understand that the authors are unlikely to go back and change this approach now, but it seems a shame that the strength of the systematic review has been weakened by the overuse of abstract screening. However, I do think that it was reasonable and pragmatic to limit to models that had been externally validated – but have encouraged the authors (above) to focus more on external validations as the "unit" of this review. However, it would be worthwhile to mention in the limitations that it would not be possible to do a head-to-head validation (which to me is an obvious next step following reviews of this type) of all models developed to date without further work.

I have a few further minor comments.

• Would have been nice to see some comparisons to similar reviews in other cancers – so the reader has an idea of whether research in oropharyngeal cancer is ahead or behind – and to discuss why this is the case.

• Always give the CIs of the c-index when reporting it. If not reported because the original study didn't report (a) tell the reader this and (b) it is often possible to calculate yourself if you have the c-index and the study size.

• Suggest using TRIPOD classifications to categorise the different study types included

• Suggest reporting the overall PROBAST (ROB and applicability) for each study assessed in a main table.

VERSION 1 - AUTHOR RESPONSE

**Reviewer: 1**<br>**Dr. Yubin Cao, Sichuan University West China Hospital of Stomatology**<br>I would like to raise some concerns for this overall good paper.	
Abstract<br>1. "secondary care" may be somewhat confusing.	Thank you. We included the setting as this is one of the required structured abstract headings by BMJ Open and we note that the models in this review are relevant to patients in secondary care. We have however changed the term to 'hospital care' as this may be clearer to non-UK readers.
Abstract<br>2. The study protocol (CRD42021248762) stated that this review will focus on head and neck cancer. However, the scope is narrower currently.	We agree that the registered protocol is for a systematic review of prognostic models in all sub-sites of head and neck cancer and that we report the findings for OPC cancer here (stated under 'Methods' heading on p3, row 36). A further review on prognostic models on laryngeal cancer based on the same protocol is currently being prepared for submission.

	The search strategy encompassed terms relating to head and neck cancer generally as well as terms relating to oropharyngeal cancer specifically.<br>We have made a note in the methods section on searches (p4, line 10): **"This search strategy was performed as part of a systematic review of prognostic models in all types of head and neck cancer, and specific terms relating to oropharyngeal cancer were included."**
Abstract<br>3. Too little information about the PROBAST assessment in the abstract.	We were unsure if this relates to PROBAST methods or findings, but we have added some detail to the abstract findings: "As all models were subject to high RoB mainly due to analysis concerns, it is uncertain how trustworthy the findings are. **Concerns included a lack of accounting for potentially missing data, model overfitting or competing risks as well as small event numbers. There were fewer concerns relating to the participant, predictor and outcome domains, though reporting was not always detailed enough to make an informed decision**."
Abstract<br>4. How many is "most"?	We have added further details to the results in the abstract to clarify what this means:<br>**"The majority (77%) of c-indices for model developments and validations were ≥0.7 indicating 'good' discriminatory ability for models predicting overall survival. For disease specific measures most (73%) c-indices for model development were also ≥0.7, but fewer (40%) were ≥0.7 for external validations. Comparisons across models and outcome measures were hampered by heterogeneity. Only two studies directly compared models in the same cohort."**
Abstract<br>5. The abstract almost provided no useful information for the readers.	We have added more findings from the PROBAST assessment (see comment 3), additional details on results (see comment 4), and further conclusions:<br>Conclusions<br>**There were no models assessed as being at low risk of bias. Given that HPV status or a radiomics score appeared to improve model discriminative performance,** further external validation of existing models to assess generalisability should focus on models that include HPV status as a variable. Development and validation of future models should be considered in HPV+ or HPV- cohorts separately to ensure representativeness.
Introduction<br>6. not seventh now.	Thank you. This estimate was taken from a 2022 article and is supported by the IARC/GLOBOCAN 2022 figures (https://gco.iarc.who.int/media/globocan/factsheets/populations/900-world-fact-sheet.pdf). We are unaware of a more recent estimate indicating a different place in the ranking. If the reviewer were to supply details of a paper reporting this then we would be happy to modify the text and include the reference.
Introduction<br>7. "include" is not adequate for the study aim.	Thank you. We have expanded the aim to: "include, **appraise and summarise** all existing evidence.." (p3, line 31)
Methods	Please see response to comment 2.

8. The difference between protocol and review is not explained with reasons.	
Methods<br>9. eligibility criterias should be an independent part from the study selection process.	We have taken this to mean that there should be a separate paragraph with eligibility criteria and have inserted this (p4, row 16):<br><br>**<u>Selection criteria</u>**<br>**Models were included if they predicted any recurrence or survival related outcomes after treatment of OPC with curative intent, included at least one clinical variable and had at least one reported external validation (supplementary Table - study selection criteria).**
Methods<br>10. Many reviewers participate in the data extraction and assessment. Are they consistent?	We believe there is consistency. For both data extraction and RoB assessment, extracted data and ratings applied by the first reviewer were always checked by one of the two lead reviewers (JD or AAF) with referral to the other in case of ambiguity or disagreement with the first reviewer.<br><br>We have clarified the text on p5, row 8/9:<br>Assessment was conducted by one reviewer (JD, AAF, BH, KS, EA, MP) and **independently** checked by **one of the two lead reviewers (JD or AAF), with referral to the other in case of ambiguity or disagreement with the first reviewer.**
Methods<br>11. Figure 3 and 4 are too small to be seen.	We believe this is due to the PDF conversion during the submission process. The original figures were submitted in the recommended formats, and we assume that they will be larger in the final version than in the PDF generated for review.
Methods<br>12. The figure title could not reflect the contents of figures accurately.	We have amended the titles from 'Overall survival' (Figure 3) and 'Disease specific measures' (Figure 4) to:<br><br>P20, row 24 and 31<br>'Discriminatory ability of models for overall survival' (Figure 3) and 'Discriminatory ability of models for disease specific outcomes' (Figure 4)
Discussion<br>13. The discussion may need some re-structuring. Readers could not grasp the logic of discussion rapidly.	Thank you. We have addressed these points together as they are linked. We have now included sub-headings to structure the discussion as we agree that this would be beneficial for the reader. This has meant re-arranging some sections.<br>We now also include a sub-heading to clearly denote where we have described the strengths and weaknesses of the systematic review. Where possible we have used subheadings suggested by Docherty and Smith (https://pubmed.ncbi.nlm.nih.gov/10231230/), with some additional, context specific, sub-headings.
Discussion<br>14. Limitations and strength should be stated.	
**Reviewer: 2**<br>**Dr. Siu-Wai CHOI, The University of Hong Kong**<br>This review is well conducted and the choice of study inclusion well documented with more than one reviewer and a starring committee	

<table>
<tr><td>15. Inclusion and exclusion criteria well defined and reasonable</td><td>Thank you for your comment.</td></tr>
<tr><td>16. Detailed description of each included study is given, and a good summary is made of which variables should be included in models for better predictive abilities.</td><td>Thank you for your comment.</td></tr>
<tr><td>17. It is important to point out that multicollinearity was poorly addressed in the studies, which the current reviewer also found to be true. Many standard statistical pitfalls are not addressed.</td><td>We thank the reviewer for highlighting that we have addressed the issue of multicollinearity and other statistical issues relating to model development and evaluation.</td></tr>
<tr><td>I want to thank the authors for this updated review</td><td></td></tr>
<tr><td colspan="2">Reviewer: 3
Dr. Hannah Harrison, University of Cambridge
I enjoyed reading this paper which gives a comprehensive overview of prognostic models for oropharyngeal cancer and highlights some important issues in this area. I liked to see the specific challenges around HPV as a key risk factor being discussed. While I am keen to recommend this paper for publication there are a couple of issues I would need to see addressed first.</td></tr>
<tr><td>18. Improved clarity on the “subject” being identified by this review. Upon careful reading, it is clear that the item/studies identified are “external validations” rather than the models themselves. However, this was not always clear to me and there are some areas in which I am not sure if I would be able to reproduce the authors approach.</td><td rowspan="2">Thank you. We have addressed comment 18. and 19. together as they are linked.
We have improved the clarity of reporting to focus on the number of models (with associated studies) rather than number of studies (results p6, row 2 onwards):
From 5,936 records screened, 15 studies were included. Using the TRIPOD classification, there was one Type1b study (development and validation using resampling), 10 Type 3 studies (development and validation using separate data) and four Type 4 studies (validation only). The 15 studies reported a total of 25 models to predict individualised outcomes (Figure 1 for full details on study selection). [14-18] The 25 models were externally validated 43 times, reported across 14 studies[3 15 27] (the remaining study[14] reported model development of a model externally evaluated in other studies). Most models were externally validated once or twice; the OS model by Fakhry (2017)[21] was externally validated in five independent cohorts, and the OroGrams OS and PFS models (Grønhøj 2018)[22] were externally validated in four independent cohorts. All model development studies, and their associated external validation studies are shown in supplementary material (model performance IPMs).

We included models based on the existence of at least one external validation study. Hence the model by Gronhoj-Larsen 2016 is</td></tr>
<tr><td>19. How study selection was managed. It seems to me that there are three types of study that could be discussed in the results section: model development studies, studies including both development and external validation, and external validation studies. It would be useful to have the number of each type identified stated explicitly early in the results.

It would be useful to understand what happened to the model</td></tr>
</table>

development studies that would have been excluded for not mentioning validation in the abstract, but were later reintroduced (?) if the model had external validations in other studies.	included despite the fact that the development of the model is reported in an article that does not include an external validation. The reviewer is correct that this model would have been excluded if there had been no additional studies undertaking an external validation of the model.
20. While the forest plots clearly lay out which studies are associated with which model, it would have be useful to reference this in the text (e.g. for model X, developed by smith et al., for which 3 external validations have been carried out).	Thank you. We have included a summary of which models are included and how many external validations were undertaken, and by which studies:<br><br>Model performance: overall survival (p9, row 14)<br><br>Discriminatory ability for overall survival was assessed by 20 models **reported across nine studies** and all reported c-indices. **The model developed by Fakhry (2017)[18] was externally validated five times[18225328], the model by Gronhoj (2018) three times[19], the model by Gronhoj-Larsen (2016)[15] twice[253], the six models by Cheng (2021) twice[16], the model by Rios-Velazquez (2014)[23] twice[2325], the model by Beesley (2021) once[3], the two models by Mes (2020) once[21], the model by Choi (2020) once[17] and the six models by Ma (2023) once.[20]**<br><br>Model performance: disease specific measures (p10, row 22)<br><br>**There were three models for PFS (by Fakhry 2017[18], Gronhoj 2018[19] and Rios-Velazquez 2014[23]), two of which were externally validated three times[18 19], and one that was externally validated once.[23] Two models developed by Mes (2020) for RFS, were externally evaluated once[21], one model for EFS (Beesley 2021) was evaluated once[3], seven models for DSS (one by Ward 2024[24] and six by Ma 2023[20]) were evaluated once[20 24], six models all developed by Ma (2023) were evaluated once, for each of LC, RC, LRC, DMFS and DFS[20], and one model (Rasmussen 2019) was evaluated once for T-, N- or M-site recurrence.[22]**
21. It was unclear to me how the RoB assessment was done. It was unclear if this was done separately for each model? Or for each external validation. As the external validations are the key "subject" here – I rather expected the reporting around bias to focus on this (and not on the development of the models). A reporting by domain of the results of the RoB assessment for each study evaluated should be included, and I wasn't able to find it.	RoB assessment was done separately for each model development and external validation cohort, so there were 68 assessments in total (25 x model developments and 43 x external evaluations). This has been clarified in the paragraph on risk of bias assessment in the methods (p5, row 6):<br>**Each model development and each external validation of models was assessed separately.**<br><br>We have also added the following to the RoB results (p8, row 7):<br>**There were 68 RoB assessments undertaken in total, for 25 model developments and 43 external validations of models (PROBAST domain ratings for each assessment are in supplementary material).**<br><br>We have also included additional supplementary material showing the RoB for each of the 68 assessments made for each PROBAST domain.

I suspect a reader who had not previously used PROBAST would have struggled to understand the distinction between the RoB and applicability assessments.	We have further corrected one statement on the RoB (p8, row 9): The overall PROBAST RoB rating was high for all **but one** of the IPMs assessments, mainly due to concerns about RoB in the analysis domain (see Figure 2); **one assessment of an external validation was rated as having insufficient information to make a judgement on overall bias.**[3]<br><br>Regarding applicability, we have added some detail to the methods section to clarify what PROBAST means by this (p5, row 14): **Applicability refers to the extent to which included models match the systematic review question in terms of participants, predictors and outcomes. Formal ratings were not generated for applicability, but PROBAST guidance informed judgements.**<br><br>We have also added a sentence to the RoB/applicability results (p8, row 26): **Regarding applicability, all studies matched the review question in terms of population, predictors and outcome, although there were two studies where a minority (up to 6.7%) of patients were not treated with curative intent.**[15 25]
22. It was not obvious to me if any of the studies identified had validated more than one model – if there were a few studies that did this, then a network meta-analysis approach may be suitable (see https://doi.org/10.1111/bju.15673)	A multivariate meta-analysis was considered to be not feasible in this review. Studies that compared external validations of models fell into two groups, (i) evaluations of iterations of their own models and (ii) evaluations of models developed by others (with or without evaluating their own). There were only two studies in the latter group (comparing three models each, two of which were the same models in both studies) hence this was insufficient to consider a multivariate meta-analysis approach. Even if there had been more studies, it is unlikely that assumptions on transferability of model performance across studies would be met due to heterogeneity in the study populations.<br><br>We have added a sentence to the discussion on why we did not consider multivariate meta-analysis (p17, row 24 onwards):<br>**"We considered, but ruled out, a multivariate meta-analysis approach for comparing model performance as undertaken in the study by Usher-Smith (2022) as evaluation of different models in the same cohort was only undertaken in two studies, and transferability assumptions were unlikely to be met."**[53]
23. A lack of specifics in the results section, in some places the reporting was vague.	Thank you, we have added some more specific estimates, e.g. giving a specific percentage for evaluations resulting in a c-index over 0.7, rather than just stating 'most' or 'the majority':<br><br>Overall survival (p9, row 21)<br>The c-index **(or AUC where c-index not reported)** was ≥0.7 ('good') for the majority of development studies **(17/22 (77%)), but only a few (4/22 (18%)) had a c-index ≥0.8 ('very good'). This was similar for** external validations across all models, **with the majority (27/34 (79%)) reporting a c-index ≥0.7**, with few external validations **(4/34 (12%)**) resulting in a c-index of ≥0.8 ('very good') (Figure 3). This was also the case for those models with lower RoB for model development RoB assessment (Beesley 2021[3] and Grønhøj 2018[19]; OS not

	predicted in Rasmussen 2019[22]) though we acknowledge that they were still rated as 'high' risk of bias using PROBAST.<br><br>Disease specific measures (p10, row 31)<br>The c-index (or AUC where c-index not reported) was **≥0.7 ('good') for 73% (36/49)** of development studies and for **40% (23/58**) of external validations across all models**. Only 22% (11/49) of development and 5% (3/58) of external validation studies found a c-index of ≥0.8 ('very good')** (Figure 4).<br>**Given the variability in models and disease specific measures, comparison of model performance across studies and outcome measures is difficult.**
24. When stating that models/studies "typically" or "less often" used a certain type of cohort or risk factor, I would encourage the authors to specify exact numbers. This will require a clear distinction to be made throughout (as discussed about) between the number of models and the number of studies.	Thank you, we have amended the section describing characteristics/risk factors as suggested.<br>We have also amended two sentences where we had referred to number of models but had cited number of studies. (See amendments throughout p 7).
25. In several places I notes statements such as "this cohort had a higher than expected number of smokers" (what was expected?) or "this cohort was deemed representative" (why?) or "event numbers were considered small" (what do you consider small? <100?) – I would advise a thorough revision of this section to avoid any ambiguity.	Thank you, we have clarified these statements and added supporting references.<br>P8, row 33:<br>**However, the Grønhøj 2018 development cohort, which had a high proportion of HPV+/p16+ patients (approximately 60%), unexpectedly included a larger than usual proportion of smokers (around 80%). This is higher than what is typically seen in clinical practice and reported in the literature for this group of patients.** (Alotaibi M, Valova V, HÄnsel T, Stromberger C, Kofla G, Olze H, Piwonski I, Albers A, Ochsenreither S, Coordes A. Impact of Smoking on the Survival of Patients With High-risk HPV-positive HNSCC: A Meta-analysis. In Vivo. 2021 Mar-Apr;35(2):1017-1026. doi: 10.21873/invivo.12345. PMID: 33622897; PMCID: PMC8045080.<br><br>Elhalawani H, Mohamed ASR, Elgohari B, Lin TA, Sikora AG, Lai SY, Abusaif A, Phan J, Morrison WH, Gunn GB, Rosenthal DI, Garden AS, Fuller CD, Sandulache VC. Tobacco exposure as a major modifier of oncologic outcomes in human papillomavirus (HPV) associated oropharyngeal squamous cell carcinoma. BMC Cancer. 2020 Sep 23;20(1):912. doi: 10.1186/s12885-020-07427-7. PMID: 32967643; PMCID: PMC7513300.)<br><br>P9, row 6:<br>**The Beesley 2021 study included a development cohort from the USA that was predominantly HPV+ or p16+, while the external validation cohort from the Netherlands was primarily p16-. This variation aligns with the known geographical differences in the**

	**prevalence of HPV+ OPSCC and is still considered representative of unselected OPC patient populations.**<br>(Mehanna H, Taberna M, von Buchwald C, Tous S, Brooks J, Mena M, Morey F, Grønhøj C, Rasmussen JH, Garset-Zamani M, Bruni L, Batis N, Brakenhoff RH, Leemans CR, Baatenburg de Jong RJ, Klussmann JP, Wuerdemann N, Wagner S, Dalianis T, Marklund L, Mirghani H, Schache A, James JA, Huang SH, O'Sullivan B, Nankivell P, Broglie MA, Hoffmann M, Quabius ES, Alemany L; HNCIG-EPIC group. Prognostic implications of p16 and HPV discordance in oropharyngeal cancer (HNCIG-EPIC-OPC): a multicentre, multinational, individual patient data analysis. Lancet Oncol. 2023 Mar;24(3):239-251. doi: 10.1016/S1470-2045(23)00013-X. Epub 2023 Feb 13. PMID: 36796393.)<br><br>We based our definition of what were considered to be small numbers of events on PROBAST (≤10 events per candidate predictor for development and <100 events for validation cohorts). We have added this information where small event numbers are first mentioned (p8, row 14).
26. Comment on the pragmatic approach to screening. I appreciate that it is challenging to screen large numbers of papers, especially at full text stage and that the resources of different teams may vary. However, based on carrying out similar reviews of prognostic models (for different cancers) I actually thought the number of identified studies (and hence the screening burden) was relatively low. I understand that the authors are unlikely to go back and change this approach now, but it seems a shame that the strength of the systematic review has been weakened by the overuse of abstract screening.<br><br>However, I do think that it was reasonable and pragmatic to limit to models that had been externally validated – but have encouraged the authors (above) to focus more on external validations as the "unit" of this review.	Thank you. We agree that the number of records to be screened was not as large as in some other prognostic systematic reviews, but nonetheless resource intensive given the inconsistent, and sometimes poor, description of models and the often equally poor description of any validation. Around 80 full texts were screened, however, this number would have been much higher (several hundred) if we had included those studies with abstracts lacking any information on validation. We believe it is unlikely studies would not have mentioned external validation as this would have formed a major part of their work. We also checked reference lists to mitigate the risk of missing studies. But we agree there is still a possibility of having missed studies and acknowledge this (p16, row 6).

However, it would be worthwhile to mention in the limitations that it would not be possible to do a head-to-head validation (which to me is an obvious next step following reviews of this type) of all models developed to date without further work.	We have also added a sentence in the limitations section to address the last point (P16, row 11):<br>**However, given the pragmatic decisions made during the study selection process and a small possibility of missing relevant models, additional searches could be performed before further work such as a head-to-head validation of all candidate models is conducted.**
I have a few further minor comments.	
27. Would have been nice to see some comparisons to similar reviews in other cancers – so the reader has an idea of whether research in oropharyngeal cancer is ahead or behind – and to discuss why this is the case.	Thank you. We have added a paragraph to the discussion. P17, row 3:<br><br>**Compared to other cancers, such as breast and prostate cancer, predictive modelling for OPSCC is relatively underdeveloped. For example, breast cancer has numerous well-established predictive models that have been developed and validated in large cohorts (ref 1), including the PREDICT model (ref 2a, 2b), which is endorsed by the National Institute for Health and Care Excellence (NICE) guidelines (ref3), and prostate cancer uses the European Association of Urology (EAU) risk group classification based on the D'Amico classification system (ref4), which is endorsed by EAU guidelines (ref5). In contrast, OPSCC modelling has lagged behind due to several factors. The rising incidence of HPV-positive OPSCC over the past two to three decades has resulted in changing risk profiles and disease behaviour, making it challenging to develop comprehensive predictive models. Additionally, there are significant gaps in understanding the genomic profile of OPSCC, particularly within HPV+ cohorts, which show considerable heterogeneity in patient characteristics and outcomes. As a result, the field needs further research to develop and validate robust predictive models that can be widely implemented in clinical practice.**<br><br>Ref1: Hueting TA, van Maaren MC, Hendriks MP, Koffijberg H, Siesling S. External validation of 87 clinical prediction models supporting clinical decisions for breast cancer patients. Breast. 2023 Jun;69:382-391. doi: 10.1016/j.breast.2023.04.003. Epub 2023 Apr 17. PMID: 37087910; PMCID: PMC10149388.<br><br>Ref2a: PREDICT: a new UK prognostic model that predicts survival following surgery for invasive breast cancer. by Wishart GC, Azzato EM, Greenberg DC, Rashbass J, Kearins O, Lawrence G, Caldas C, Pharoah PDP. Breast Cancer Res. 2010; 12(1): R1. Published online 2010 January 6. doi: 10.1186/bcr2464. PMCID: PMC2880419.<br><br>Ref2b: Stabellini N, Cao L, Towe CW, Miller ME, Sousa-Santos AH, Amin AL, Montero AJ. Validation of the PREDICT Prognostication Tool in US Patients With Breast Cancer. J Natl Compr Canc Netw. 2023 Oct;21(10):1011-1019.e6. doi: 10.6004/jnccn.2023.7048. PMID: 37856198.

	Ref3: National Institute for Health and Care Excellence (NICE). (2024). Early and locally advanced breast cancer: diagnosis and management (NICE guideline [NG101]). London: National Institute for Health and Care Excellence (NICE); 2024 Jan 16. https://www.nice.org.uk/guidance/ng101 [last accessed 30/08/2024]<br><br>Ref4: Boorjian SA, Karnes RJ, Rangel LJ, Bergstralh EJ, Blute ML. Mayo Clinic validation of the D'amico risk group classification for predicting survival following radical prostatectomy. J Urol. 2008 Apr;179(4):1354-60; discussion 1360-1. doi: 10.1016/j.juro.2007.11.061. Epub 2008 Mar 4. PMID: 18289596.<br><br>Ref5: Cornford P, van den Bergh RCN, Briers E, Van den Broeck T, Brunckhorst O, Darraugh J, Eberli D, De Meerleer G, De Santis M, Farolfi A, Gandaglia G, Gillessen S, Grivas N, Henry AM, Lardas M, van Leenders GJLH, Liew M, Linares Espinos E, Oldenburg J, van Oort IM, Oprea-Lager DE, Ploussard G, Roberts MJ, Rouvière O, Schoots IG, Schouten N, Smith EJ, Stranne J, Wiegel T, Willemse PM, Tilki D. EAU-EANM-ESTRO-ESUR-ISUP-SIOG Guidelines on Prostate Cancer-2024 Update. Part I: Screening, Diagnosis, and Local Treatment with Curative Intent. Eur Urol. 2024 Aug;86(2):148-163. doi: 10.1016/j.eururo.2024.03.027. Epub 2024 Apr 13. PMID: 38614820.
28. Always give the CIs of the c-index when reporting it. If not reported because the original study didn't report (a) tell the reader this and (b) it is often possible to calculate yourself if you have the c-index and the study size.	We agree and have noted in the figure legends that not all CIs of the c-index were reported in the original studies.
29. Suggest using TRIPOD classifications to categorise the different study types included	We have added a statement where we first describe the results to show the types of studies according to the TRIPOD classification (p 6, row 2):<br><br>**Using the TRIPOD classification, there was one Type1b study (development and validation using resampling), 10 Type 3 studies (development and validation using separate data) and four Type 4 studies (validation only).**
30. Suggest reporting the overall PROBAST (ROB and applicability) for each study assessed in a main table.	We have now included supplementary material (9) showing the RoB for each PROBAST domain for each model development or external validation assessment.

VERSION 2 - REVIEW

Reviewer **3**

Name **Harrison, Hannah**

Affiliation **University of Cambridge, Department of Public Health and Primary Care**

Date **26-Sep-2024**

COI

Many thanks for such a comprehensive revision and for addressing the points I raised in my previous review so carefully. I commend you on a well written and useful review of the models in this area (that I am sure to cite in future). I have a couple of very small outstanding points I wanted to raise.

1. Thank you for adding useful context by reporting on the disparity between this area for H&N cancers and other cancers. While breast and prostate cancers are the obvious gold standards in this area - it might be nice to relate to work for cancer that has more similarities in terms of incidence. When considering the broader field, I don't necessarily think that oropharyngeal is "lagging" - but instead there is a similar picture to that seen in other less common cancers (i.e. this is a cross-cancer challenge).

2. Thanks for adding the comment on CIs to the figure caption. I note though, that c-indices are reported in the text without CIs, even though CIs are reported for that model on the forrest plot. I did struggle with this a bit as couldn't read the forrest plots easily in their current format so apologies I couldn't cross-check most instances but.. noted as one exmple the study by Mes et al. has CIs shown in the forrest plots but not in the text "The Mes (2020) clinical model (which includes N-stage, age and sex) had a markedly lower c-index for the development cohort (0.57) compared with the same model including 25 radiomics features (0.73); this study was in HPV- patients only". Please double check this throughout, it is really important to report the CIs whenever they are avaliable.

VERSION 2 - AUTHOR RESPONSE

<u>Response to peer review comments</u>

We thank the reviewer for their comments. Please find our responses below.

1. Thank you for adding useful context by reporting on the disparity between this area for H&N cancers and other cancers. While breast and prostate cancers are the obvious gold standards in this area - it might be nice to relate to work for cancer that has more similarities in terms of incidence. When considering the broader field, I don't necessarily think that oropharyngeal is "lagging" - but instead there is a similar picture to that seen in other less common cancers (i.e. this is a cross-cancer challenge).	We have added some text to highlight that OPSCC is not the only cancer where the prognostic model field is less developed compared with other cancers (p17, row 4 onwards in marked copy):<br><br>Compared to other cancers, such as breast and prostate cancer, predictive modelling **for less common cancers, including OPSCC, oral cavity cancer, laryngeal, nasopharyngeal and hypopharyngeal cancer** is relatively

	underdeveloped **and some way away from routine clinical implementation.** [55]
2. Thanks for adding the comment on CIs to the figure caption. I note though, that c-indices are reported in the text without CIs, even though CIs are reported for that model on the forrest plot. I did struggle with this a bit as couldn't read the forrest plots easily in their current format so apologies I couldn't cross-check most instances but.. noted as one exmple the study by Mes et al. has CIs shown in the forrest plots but not in the text "The Mes (2020) clinical model (which includes N-stage, age and sex) had a markedly lower c-index for the development cohort (0.57) compared with the same model including 25 radiomics features (0.73); this study was in HPV- patients only". Please double check this throughout, it is really important to report the CIs whenever they are avaliable.	Thank you. We have added the CI where we report a c-index for a single study, and also noted where the C-index is not available (p9, row 33 and 34; p11 row 4, 5 and 8 in marked copy).
